# Comparisons of exacerbations and mortality among regular inhaled therapies for patients with stable chronic obstructive pulmonary disease: Systematic review and Bayesian network meta-analysis

**DOI:** 10.1371/journal.pmed.1002958

**Published:** 2019-11-15

**Authors:** Hyun Woo Lee, Jimyung Park, Junwoo Jo, Eun Jin Jang, Chang-Hoon Lee

**Affiliations:** 1 Division of Pulmonary and Critical Care Medicine, Department of Internal Medicine, Seoul National University Hospital, Seoul, South Korea; 2 Department of Statistics, Kyungpook National University, Daegu, South Korea; 3 Department of Information Statistics, Andong National University, Andong, South Korea; University of Pittsburgh, UNITED STATES

## Abstract

**Background:**

Although exacerbation and mortality are the most important clinical outcomes of stable chronic obstructive pulmonary disease (COPD), the drug classes that are the most efficacious in reducing exacerbation and mortality among all possible inhaled drugs have not been determined.

**Methods and findings:**

We performed a systematic review (SR) and Bayesian network meta-analysis (NMA). We searched Medline, EMBASE, the Cochrane Central Register of Controlled Trials, ClinicalTrials.gov, the European Union Clinical Trials Register, and the official websites of pharmaceutical companies (from inception to July 9, 2019). The eligibility criteria were as follows: (1) parallel-design randomized controlled trials (RCTs); (2) adults with stable COPD; (3) comparisons among long-acting muscarinic antagonists (LAMAs), long-acting beta-agonists (LABAs), inhaled corticosteroids (ICSs), combined treatment (ICS/LAMA/LABA, LAMA/LABA, or ICS/LABA), or a placebo; and (4) study duration ≥ 12 weeks. This study was prospectively registered in International Prospective Register of Systematic Reviews (PROSPERO; CRD42017069087). In total, 219 trials involving 228,710 patients were included. Compared with placebo, all drug classes significantly reduced the total exacerbations and moderate to severe exacerbations. ICS/LAMA/LABA was the most efficacious treatment for reducing the exacerbation risk (odds ratio [OR] = 0.57; 95% credible interval [CrI] 0.50–0.64; posterior probability of OR > 1 [P(OR > 1)] < 0.001). In addition, in contrast to the other drug classes, ICS/LAMA/LABA and ICS/LABA were associated with a significantly higher probability of reducing mortality than placebo (OR = 0.74, 95% CrI 0.59–0.93, P[OR > 1] = 0.004; and OR = 0.86, 95% CrI 0.76–0.98, P[OR > 1] = 0.015, respectively). The results minimally changed, even in various sensitivity and covariate-adjusted meta-regression analyses. ICS/LAMA/LABA tended to lower the risk of cardiovascular mortality but did not show significant results. ICS/LAMA/LABA increased the probability of pneumonia (OR for triple therapy = 1.56; 95% CrI 1.19–2.03; P[OR > 1] = 1.000). The main limitation is that there were few RCTs including only less symptomatic patients or patients at a low risk.

**Conclusions:**

These findings suggest that triple therapy can potentially be the best option for stable COPD patients in terms of reducing exacerbation and all-cause mortality.

## Abstract 2

In this systematic review and network meta-analysis, Chang-Hoon Lee and colleagues compare efficacy and safety of drugs and drug combinations for treating COPD.

## Author summary

**Why was this study done?:**

- Pharmacologic inhaled therapies are cornerstones for treating patients with stable chronic obstructive pulmonary disease (COPD) to improve their health condition and reduce the future risk of exacerbation and mortality while minimizing significant drug-related adverse events. However, the best inhaled therapy for reducing exacerbation, mortality, and adverse events is unknown.
- As previous evidence is not enough to determine which inhaled therapy is expected to be most beneficial, a new attempt using a network meta-analysis (NMA) is needed to provide clinicians useful information in choosing one of the various inhaled therapies.
- NMAs with Bayesian statistics can compare drugs directly and indirectly, calculate the rank of the drugs in terms of efficacy and safety, and are suitable for analysing rare events, such as mortality.

**What did the researchers do and find?:**

- We conducted a systematic review (SR) and Bayesian NMA of 219 trials involving 228,710 patients to compare exacerbation, mortality, and adverse events among all regular inhaled drug classes, including inhaled corticosteroid (ICS)/long-acting muscarinic antagonist (LAMA)/long-acting beta-agonist (LABA), LAMA/LABA, ICS/LABA, LAMA, LABA, ICS, and placebo.
- In our NMA, ICS/LAMA/LABA was the most effective treatment for reducing total exacerbations and all-cause mortality. ICS/LABA also reduces all-cause mortality compared with placebo in our NMA. All drug classes reduced the exacerbation risk compared with placebo.

**What do these findings mean?:**

- All regular inhaled drug classes are beneficial for stable COPD patients in terms of decreasing the exacerbation risk.
- Compared with the other drug classes, triple therapy with ICS/LAMA/LABA could potentially be the best option for high-risk patients with stable COPD in terms of reducing both exacerbation and all-cause mortality.

## Introduction

Inhaled drugs, including long-acting muscarinic antagonists (LAMAs), long-acting beta-agonists (LABAs), inhaled corticosteroids (ICSs), and combination drugs, have been used as cornerstone therapies for patients with stable chronic obstructive pulmonary disease (COPD) for more than 10 years. Reducing the exacerbation risk is an important goal in the treatment of stable COPD patients, and many studies have revealed that single or combined inhaled drug classes achieve this goal. However, recent studies are inconsistent regarding the drug classes that are the most efficacious in reducing exacerbation [1–3]. The FLAME trial showed that LAMA/LABA was superior to ICS/LABA in reducing the exacerbation risk [1]. In contrast, ICS/LABA had better efficacy in reducing exacerbation than LAMA/LABA in the IMPACT trial [2]. The IMPACT and TRIBUTE trials also showed that ICS/LAMA/LABA significantly reduced the exacerbation risk more than ICS/LABA and LAMA/LABA [2,3].

In addition, the mortality risk has been compared among drug classes only in a limited number of studies. Prior systematic reviews (SRs) and meta-analyses rarely compared the risks of mortality among the various drug classes. A recent pairwise meta-analysis showed that ICS/LAMA/LABA was associated with a lower risk of exacerbations but failed to reveal the efficacy in reducing mortality in any drug class [4]. Two additional network meta-analyses (NMAs) have been performed, but these studies included neither recent important large randomized controlled trials (RCTs) [5] nor RCTs investigating ICS/LAMA/LABA [6,7]. Safety issues, including pneumonia with ICS [8–10] and cardiovascular events with LAMA and LABA [11,12], also cannot be ignored.

SRs and NMAs enable the evaluation of the efficacy and safety of all possible inhaled drugs for stable COPD, simultaneously generating direct and indirect evidence. We used Bayesian statistics to identify the drug that is the best option for not only reducing exacerbation and mortality but also avoiding adverse events. One advantage of Bayesian NMAs is that they compare drugs simultaneously and calculate the efficacy of drugs using posterior probability in contrast to previous frequentist pairwise meta-analyses. In addition, Bayesian methods are suitable for analysing rare events, such as mortality. This method could provide useful information to aid decision-making [13].

Further elucidating the inhaled drugs that have better efficacy in stable COPD patients is still required. We used Bayesian statistics to compare drugs and drug combinations in their ability to reduce exacerbations and mortality and minimize adverse events.

## Methods

### Protocol and registration

This SR followed the guidance of the Preferred Reporting Items for Systematic reviews and Meta-analyses (PRISMA) extension statement for reporting SRs that incorporate NMAs of healthcare interventions [14] and the BayesWatch guidelines for reporting results that apply Bayesian methods [15]. The PRISMA 2009 checklist was used for standardized reporting of this SR and NMA (S1 PRISMA checklist) [16]. We registered the final protocol with the International Prospective Register of Systematic Reviews (PROSPERO; CRD42017069087).

### Eligibility criteria

The eligibility criteria for the present study were as follows: (1) parallel-design RCTs fulfilling the criteria of the Design Algorithm for Medical Literature on Intervention (DAMI) [17]; (2) RCTs including adult patients with stable COPD; (3) RCTs comparing the outcomes of treatment with LAMAs, LABAs, ICSs, combined treatment, or placebo; (4) RCTs conducted for 12 weeks or longer; (5) RCTs reporting acute exacerbations, mortality, or adverse events; and (6) human studies written in English. The adverse events included cardiovascular-disease–related mortality, major adverse cardiovascular events (MACEs), and pneumonia.

### Information sources and search

We searched MEDLINE, EMBASE, the Cochrane Central Register of Controlled Trials, ClinicalTrials.gov, the European Union Clinical Trial Register, the GlaxoSmithKline Study Register, the AstraZeneca Clinical Trials website, the Novartis clinical trial results website, and the Boehringer Ingelheim clinical study results website from their inception (search date: July 9, 2019). We contacted the authors and representatives of the pharmaceutical companies, including GlaxoSmithKline, Boehringer Ingelheim, AstraZeneca, Novartis, and Kolon (the dealer company of Chiesi), to obtain additional data regarding unpublished trials, conference abstracts, and study protocols. The Peer Review of Electronic Search Strategies (PRESS) checklist was referred to when drafting the search strategy, which was revised by discussion as necessary [18]. The search terms were “COPD” AND inhaled drugs (“ICS” OR “LAMA” OR “LABA”) AND randomized protocol design, which were composed of controlled vocabulary and free text. LAMAs included tiotropium with a dry powder inhaler or soft mist inhaler, aclidinium, umeclidinium, and glycopyrronium. LABAs included salmeterol, formoterol, vilanterol, and indacaterol. ICSs included beclomethasone dipropionate, budesonide, fluticasone propionate, fluticasone furoate, triamcinolone, and mometasone. The final version of the search strategy is described in S1 Text and PROSPERO. Manual searches using the study identifier or references of each study were also conducted.

### Study selection

We reviewed and selected studies according to the PRISMA flow diagram [16]. Duplicated studies were removed primarily based on the title and name of the first author. After three independent reviewers (HWL, JMP, and CHL) achieved over 90% agreement in a final calibration exercise to improve the interobserver reliability by title and abstract with a sample of 200 randomly selected studies, the three reviewers individually screened the abstracts and titles of all potentially eligible studies. Two independent reviewers (HWL and JMP) performed a full-text review to assess whether the screened studies met the eligibility criteria of our study. We resolved any conflicts or disagreements regarding eligibility by referring to and discussing the original articles with a third reviewer.

### Data collection process and data items

During a calibration exercise, we coordinated the data collection method and revised the pre-piloted formats for evaluating study quality and synthesizing evidence. The data extraction was conducted by two independent reviewers (HWL and JMP). The retrieved data items included basic study information (e.g., year of study, study duration, device used for treatment, study outcomes, number of patients included in the intention-to-treat analysis, and research sponsorship), baseline characteristics of the study population (e.g., mean age, sex, body mass index [BMI], smoking status, and ethnicity), clinical information of the study population (e.g., time since COPD diagnosis, whether emphysema or chronic bronchitis was also present, severity of COPD, post-bronchodilator forced expiratory volume in the first second [FEV1], history of total exacerbations in the past year, history of total exacerbations ≥2 or severe exacerbations ≥1 in the past year, modified medical research council [mMRC] dyspnoea scale score, COPD assessment test [CAT] score, percentage of eosinophils in serum, and reversibility), and study outcomes (e.g., number of patients with total exacerbations of COPD, number of patients with moderate to severe exacerbations of COPD, number of all-cause mortality cases, and number of patients with adverse events until the final follow-up). We extracted the number of patients with acute exacerbations from the results if the data were available. If an acute exacerbation was measured as the time to the first event or presented with a Kaplan-Meier curve, we recovered the raw data by digitization [19]. The severity of COPD exacerbation was determined by the Exacerbations of Chronic Pulmonary Disease Tool (EXACT) [20] or health care resource use (HCRU) [21]. Reversibility was defined as the difference between post-bronchodilator and pre-bronchodilator FEV1% of predicted. The data were extracted independently by two individual reviewers who double-checked each other’s results, which were verified by a third reviewer. Insufficient data and information were addressed by sending an e-mail request to the study authors. Any controversy or disagreement regarding the data extraction process was resolved by discussion.

### Geometry of the network

All drugs in the eligible head-to-head comparisons were examined to determine whether the classes of comparable drugs were the same. If a comparison of different drugs in the same drug class was identified as an auto-loop in the geometry, the drugs were merged into the same group by manually reviewing the extracted data. In the geometry of the network at the drug class level, each drug class was expressed as a node, and a direct comparison of two different drug classes in an RCT was shown as a link between the nodes. The thickness of the edges was proportionally weighted according to the number of direct treatment comparisons, and the size of the nodes reflected the number of studies using the treatment.

### Risk of bias within and across individual studies

After a calibration exercise and discussions to reach agreement, two reviewers (HWL and JMP) independently appraised the risk of bias of each included study according to the 7 areas provided in the Cochrane Risk-of-Bias tool [22]. Further controversy or disagreement regarding the risk-of-bias assessment was resolved by additional discussion.

### Data synthesis and analysis

In the present Bayesian NMA, we used a random-effects model with a heterogeneous variance structure [23]. The prior distributions of the parameters in the Bayesian model were assumed to be noninformative and were assumed to have a normal or uniform distribution. We estimated the posterior median odds ratio (OR) with 95% credible intervals (CrIs) and the posterior probability of the OR exceeding 1 (P[OR >1]) to measure the association between the inhaled drugs and treatment outcomes. A significant result was assumed if P(OR > 1) or the posterior probability of a regression coefficient (beta) lower than 0 (P[beta < 0]) was less than 0.025 or more than 0.975.

The best treatment reducing each outcome was determined based on the relative probability of being the most effective treatment based on the surface under the cumulative ranking curve (SUCRA) [24]. A sensitivity analysis was conducted at the study level based on FEV1, previous history of exacerbation, symptoms (mMRC or CAT), and study duration. In the network meta-regression analysis, the regression coefficients of the covariates (FEV1, previous history of exacerbation, serum eosinophil, mMRC, and reversibility) are presented to explain the effect of each covariate on the outcome [25]. Detailed information regarding the data synthesis, sensitivity and regression analysis, investigation of publication bias, and consistency assumptions [26] is provided in S2 Text and the PROSPERO protocol.

## Results

### Study selection and network geometry

**Fig 1:**
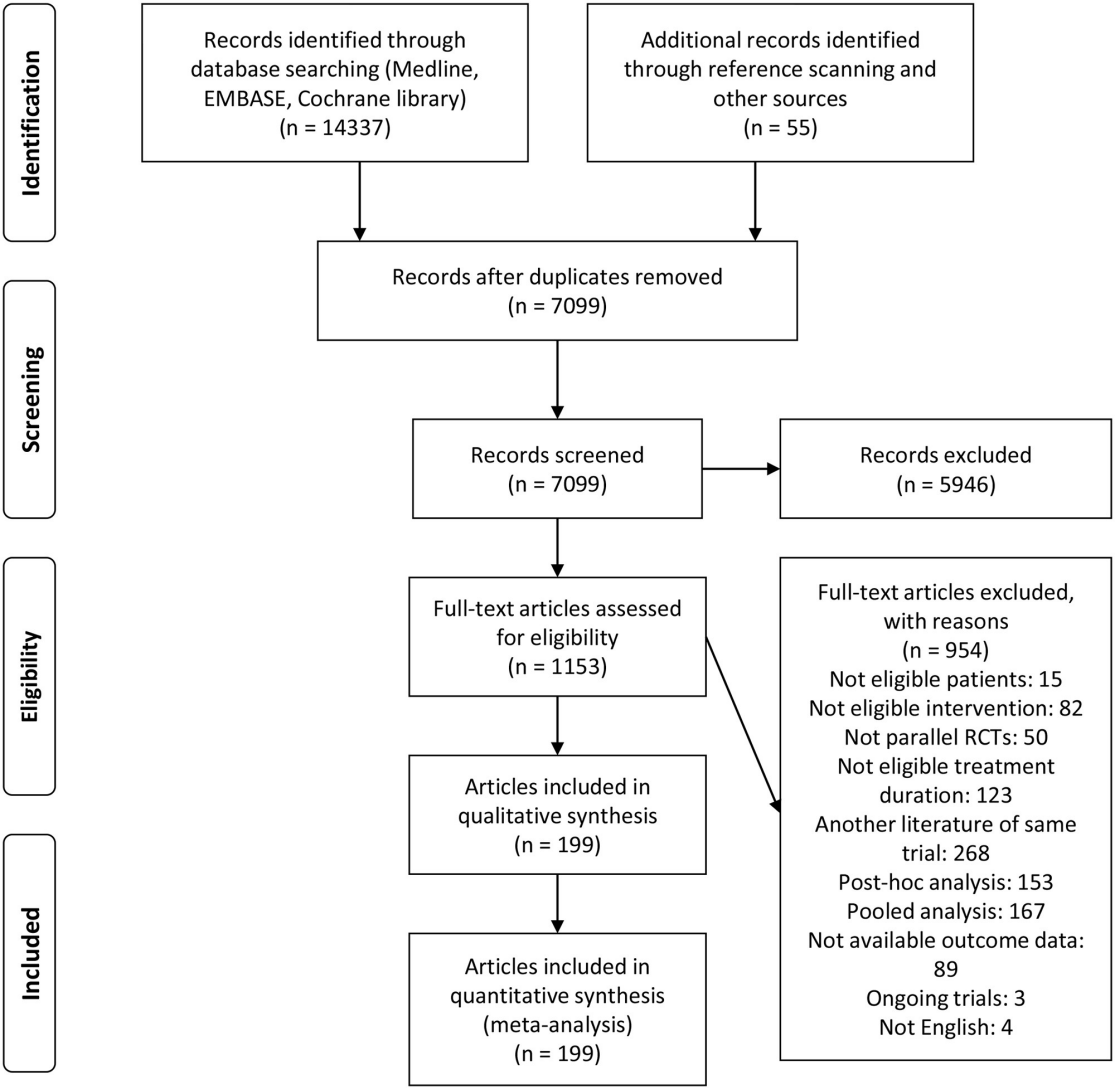
PRISMA flow chart of the study selection for the NMA. NMA, network meta-analysis; PRISMA, Preferred Reporting Items for Systematic reviews and Meta-analyses; RCT, randomised controlled trial.

**Fig 2:**
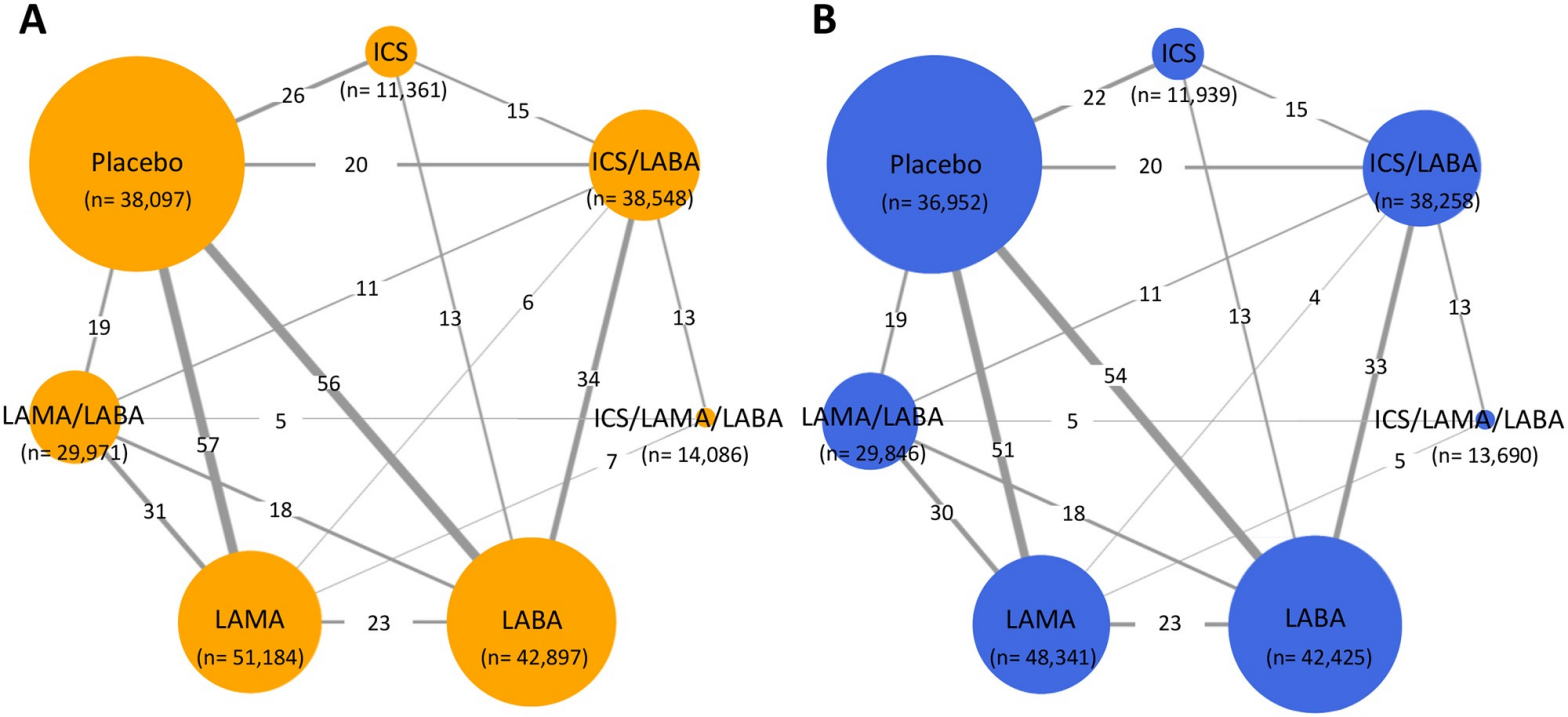
Network geometry used to evaluate the risk of total exacerbations (A) and all-cause mortality (B). ICS, inhaled corticosteroid; LABA, long-acting beta-agonist; LAMA, long-acting muscarinic antagonist.

After the removal of duplicate references, we identified 7,044 articles, and 1,153 potentially relevant articles were found after screening the titles and abstracts (Fig 1). After a full-text review, we found 199 articles involving 228,710 patients in 219 RCTs that met the eligibility criteria of the present study. The published and unpublished articles are listed in S3 Text. The network geometry addressing total exacerbations is expressed in orange (Fig 2A), while the network geometry addressing all-cause mortality is shown in blue (Fig 2B).

### Study characteristics

The characteristics of the included studies and participants are summarized in Table 1. Since the 2000s, the number of studies investigating regular inhaled therapies for COPD has increased. More than half of these studies were conducted for >12 to ≤24 weeks, and approximately one-quarter of the studies had a duration of >48 and ≤72 weeks. A dry powder inhaler was used in 75.3% of the studies, while a metered dose inhaler and soft mist inhaler were used in 23.3% and 9.6% of the studies, respectively. The total exacerbations and all-cause mortality were examined in 95.0% of the studies, while moderate to severe exacerbations were reported in 30.6% of the studies. MACE was reported in a relatively small number of studies (11.9%). The most commonly studied COPD patients were classified as moderate to severe (Global Initiative for Chronic Obstructive Lung Disease [GOLD] stage II–III) or moderate to very severe (GOLD stage II–IV).

**Table 1:** Baseline characteristics of the 219 trials eligible for inclusion and 228,710 patients with stable COPD.

Characteristic	Number of trials	Percentage
**Study characteristics**		
***Published year***		
<2000	6	2.7%
2000–2004	26	11.9%
2005–2009	40	18.3%
2010–2014	69	31.5%
2015–2019^a^	66	30.1%
Not described	12	5.5%
***Follow-up duration***, ***wk***		
>12 to ≤24	115	52.5%
>24 to ≤48	38	17.4%
>48 to ≤72	51	23.3%
>72 to ≤120	4	1.8%
>120	11	5.0%
***Type of inhaler device***^b^		
Dry powder inhaler	165	75.3%
Metered dose inhaler	51	23.3%
Soft mist inhaler	21	9.6%
Unclear	1	0.5%
***Evaluated outcome***		
Total exacerbation	208	95.0%
Moderate to severe exacerbation	67	30.6%
All-cause mortality	190	86.8%
Cardiovascular disease-related mortality	109	49.8%
MACE	26	11.9%
Pneumonia	147	67.1%
***Research sponsorship***		
Declaration of commercial sponsorship	199	90.9%
Without declaration of commercial sponsorship	20	9.1%
**Patient characteristics**		
***Mean age***, ***y***		
≤50	2	0.9%
>50 to ≤65	169	77.2%
>65	44	20.1%
Unclear	4	1.8%
***Male***, ***%***		
≤50	6	2.7%
>50 to ≤75	133	60.7%
>75	76	34.7%
Unclear	4	1.8%
***BMI***		
≤25	19	8.7%
>25	60	27.4%
Unclear	140	63.9%
***Current smoker***, ***%***		
≤25	5	2.3%
>25 to ≤50	114	52.1%
>50 to ≤75	41	18.7%
>75	6	2.7%
Unclear	53	24.2%
***Pack-years***		
≤15	2	0.9%
>15 to ≤30	6	2.7%
>30	141	64.4%
Unclear	70	32.0%
***Time since COPD diagnosis***, ***y***		
≤5	6	2.7%
>5 to ≤10	77	35.2%
>10	10	4.6%
Unclear	126	57.5%
***Dominant ethnicity (>80%)***		
Caucasian	83	37.9%
Asian	14	6.4%
Caucasian and Asian	17	7.8%
Caucasian and other races	1	0.5%
Non-Hispanic	1	0.5%
Unclear	103	47.0%
***Diagnosis of emphysema***, ***%***		
≤50%	6	2.7%
>50%	12	5.5%
Unclear	201	91.8%
***Diagnosis of chronic bronchitis***, ***%***		
≤50%	5	2.3%
>50%	17	7.8%
Unclear	197	90.0%
***Severity of COPD (FEV1%)***		
Mild to moderate (>50%)	4	1.8%
Mild to severe (>30%)	3	1.4%
Mild to very severe (all)	2	0.9%
Moderate (50%–80%)	9	4.1%
Moderate to **severe (30%–80%)**	90	41.1%
Moderate to very severe (≤80%)	85	38.8%
Severe **(30%–50%)**	3	1.4%
Severe to very severe (≤50%)	20	9.1%
Unclear	3	1.4%

^a^Articles were searched until July 9, 2019. The actual duration of 2015–2019 is approximately 54 months.

^b^Different types of devices were used together in 19 studies.

**Abbreviations:** BMI, body mass index; FEV1, forced expiratory volume in the first second; COPD, chronic obstructive pulmonary disease; MACE, major adverse cardiovascular event

### Risk of bias within studies and across studies

**Fig 3:**
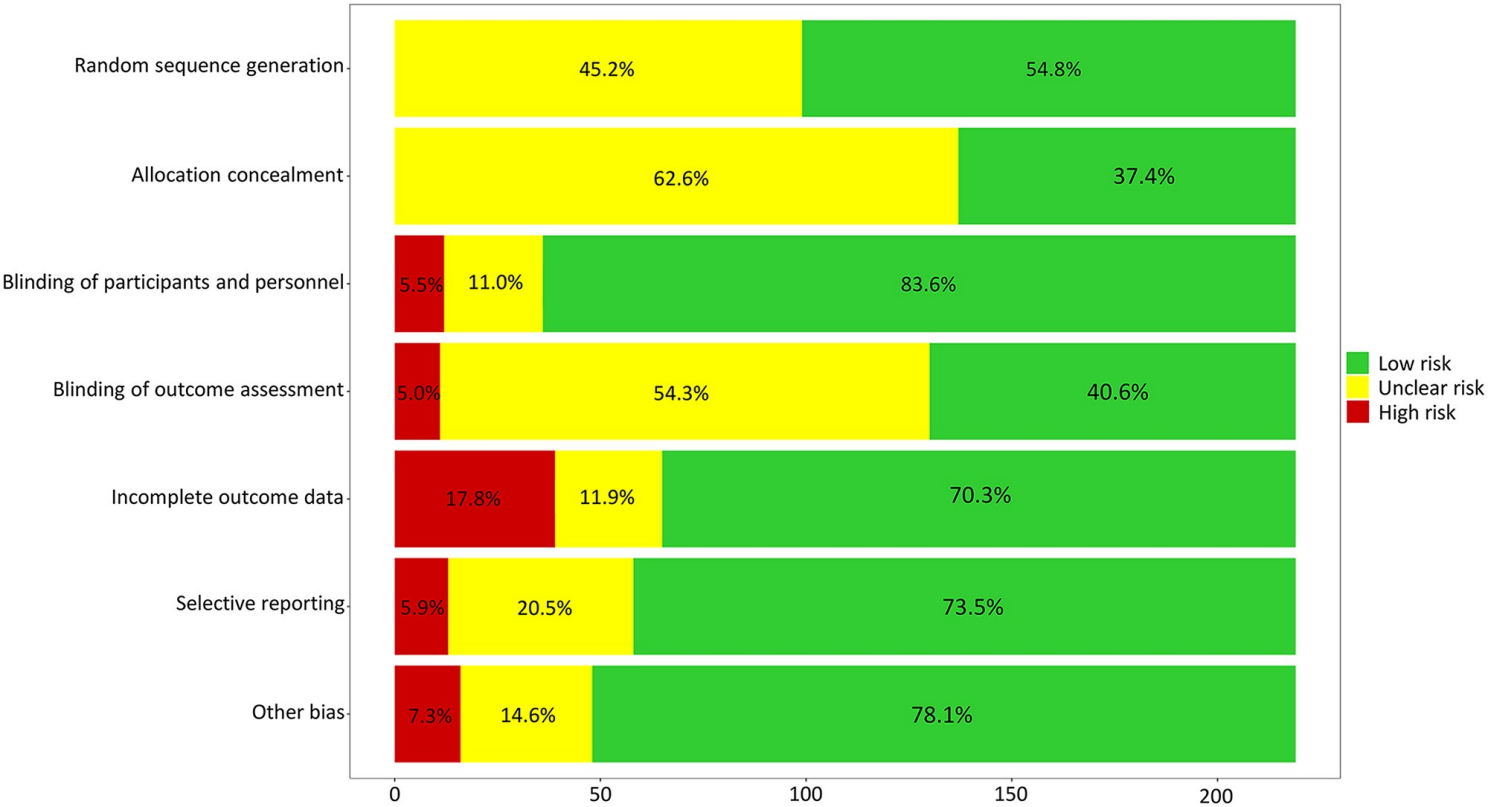
Assessment of the risk of bias in the included studies.

In general, we assessed the risk of bias as acceptable for our NMA (Fig 3, S1 Table). No substantial risk of bias was detected in random sequence generation and allocation concealment. Blinding of the participants and personnel was conducted well in most included RCTs. Our primary and secondary outcomes were unlikely influenced by incomplete outcome data because the reasons for withdrawal or follow-up loss were balanced. Selective reporting bias or additional sources of bias were rarely found. In the analyses exploring the potential risk of bias across the studies, publication bias and selective reporting were rarely found (S2 Table). Although significant publication bias could not be excluded using the Egger test in a few comparisons, we considered that there was no significant risk of publication bias because the results were unchanged after adjustment using the trim and fill method.

### Total exacerbations

**Fig 4:**
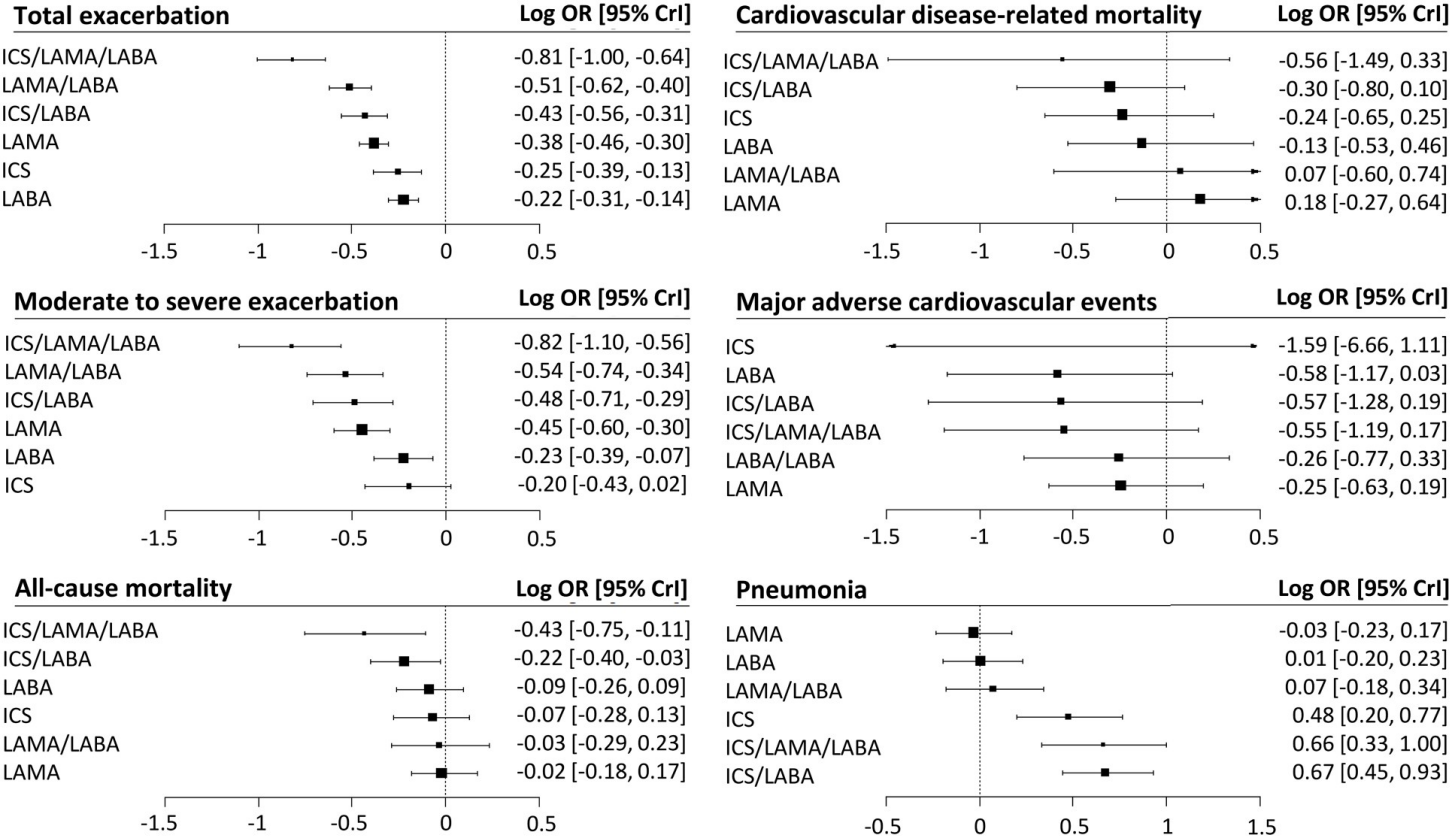
Forest plots of the risk of exacerbations, mortality, and adverse events compared with a placebo. CrI, credible interval; ICS, inhaled corticosteroid; LABA, long-acting beta-agonist; LAMA, long-acting muscarinic antagonist; OR, odds ratio.

The effectiveness of reducing the total exacerbations of COPD was evaluated in 226,117 patients in 208 RCTs (Fig 4). Compared with placebo, all drug classes showed significant benefits in reducing total exacerbations. ICS/LAMA/LABA had a higher probability of reducing total exacerbations than the other drugs (Table 2, S3 Table, and S1 Fig). Compared with each single drug, all dual combination drugs—including ICS/LABA and LAMA/LABA—had a higher probability of reducing total exacerbations. LAMA had a higher probability of reducing total exacerbation than LABA.

**Table 2:** Results of Bayesian NMAs of exacerbations, mortality, and adverse events according to the drug class.

NMA estimate OR (95% CrI)	Placebo	ICS/LAMA/LABA	LAMA/LABA	ICS/LABA	LAMA	LABA	ICS
**Total exacerbations (208 studies, 226,117 patients)**							
**Placebo**	1						
**ICS/LAMA/LABA**	0.57 (0.5–0.64)	1					
**LAMA/LABA**	0.7 (0.65–0.76)	1.24 (1.1–1.41)	1				
**ICS/LABA**	0.74 (0.68–0.81)	1.31 (1.16–1.47)	1.06 (0.96–1.16)	1			
**LAMA**	0.77 (0.73–0.81)	1.35 (1.2–1.53)	1.09 (1.02–1.18)	1.03 (0.95–1.13)	1		
**LABA**	0.86 (0.81–0.9)	1.51 (1.34–1.72)	1.22 (1.13–1.32)	1.15 (1.06–1.25)	1.12 (1.05–1.19)	1	
**ICS**	0.84 (0.76–0.91)	1.48 (1.28–1.7)	1.19 (1.07–1.33)	1.13 (1.02–1.25)	1.09 (0.99–1.2)	0.98 (0.89–1.07)	1
**Moderate to severe exacerbations (67 studies, 116,131 patients)**							
**Placebo**	1						
**ICS/LAMA/LABA**	0.56 (0.47–0.68)	1					
**LAMA/LABA**	0.69 (0.6–0.79)	1.22 (1.05–1.44)	1				
**ICS/LABA**	0.71 (0.61–0.82)	1.27 (1.07–1.48)	1.04 (0.9–1.18)	1			
**LAMA**	0.73 (0.66–0.81)	1.3 (1.1–1.56)	1.06 (0.95–1.2)	1.03 (0.9–1.19)	1		
**LABA**	0.85 (0.76–0.95)	1.51 (1.27–1.82)	1.24 (1.09–1.41)	1.2 (1.05–1.37)	1.17 (1.05–1.29)	1	
**ICS**	0.87 (0.74–1.02)	1.54 (1.25–1.91)	1.26 (1.05–1.51)	1.22 (1.04–1.44)	1.19 (1–1.4)	1.02 (0.87–1.19)	1
**All-cause mortality (190 studies, 221,451 patients)**							
**Placebo**	1						
**ICS/LAMA/LABA**	0.74 (0.59–0.93)	1					
**LAMA/LABA**	0.98 (0.82–1.18)	1.32 (1.05–1.66)	1				
**ICS/LABA**	0.86 (0.76–0.98)	1.16 (0.95–1.42)	0.88 (0.73–1.05)	1			
**LAMA**	0.98 (0.88–1.12)	1.33 (1.07–1.67)	1.01 (0.86–1.19)	1.15 (0.99–1.34)	1		
**LABA**	0.94 (0.83–1.07)	1.27 (1.02–1.58)	0.96 (0.8–1.16)	1.1 (0.97–1.24)	0.95 (0.82–1.1)	1	
**ICS**	0.95 (0.82–1.09)	1.29 (1.01–1.63)	0.97 (0.79–1.2)	1.11 (0.94–1.28)	0.97 (0.8–1.14)	1.01 (0.87–1.17)	1
**Cardiovascular-disease–related mortality (109 studies, 142,622 patients)**							
**Placebo**	1						
**ICS/LAMA/LABA**	0.68 (0.36–1.26)	1					
**LAMA/LABA**	1.05 (0.66–1.67)	1.54 (0.84–2.91)	1				
**ICS/LABA**	0.81 (0.57–1.07)	1.19 (0.67–2.09)	0.77 (0.49–1.17)	1			
**LAMA**	1.13 (0.83–1.56)	1.66 (0.87–3.29)	1.08 (0.67–1.74)	1.4 (0.98–2.11)	1		
**LABA**	0.91 (0.69–1.38)	1.35 (0.73–2.76)	0.87 (0.54–1.5)	1.13 (0.84–1.8)	0.81 (0.56–1.26)	1	
**ICS**	0.85 (0.64–1.19)	1.25 (0.67–2.49)	0.81 (0.49–1.38)	1.05 (0.78–1.58)	0.75 (0.5–1.16)	0.93 (0.63–1.29)	1
**MACEs (26 studies, 52,881 patients)**							
**Placebo**	1						
**ICS/LAMA/LABA**	0.68 (0.44–1.12)	1					
**LAMA/LABA**	0.84 (0.59–1.26)	1.23 (0.86–1.76)	1				
**ICS/LABA**	0.68 (0.41–1.14)	0.99 (0.67–1.43)	0.81 (0.54–1.19)	1			
**LAMA**	0.84 (0.65–1.14)	1.24 (0.82–1.81)	1.01 (0.74–1.34)	1.25 (0.79–1.96)	1		
**LABA**	0.67 (0.44–1.02)	0.98 (0.58–1.61)	0.8 (0.51–1.2)	0.99 (0.58–1.68)	0.79 (0.52–1.19)	1	
**ICS**	0.33 (0.01–2.15)	0.48 (0.01–3.01)	0.4 (0.01–2.47)	0.49 (0.02–2.99)	0.39 (0.01–2.5)	0.5 (0.02–3.16)	1
**Pneumonia (147 studies, 195,628 patients)**							
**Placebo**	1						
**ICS/LAMA/LABA**	1.58 (1.26–2)	1					
**LAMA/LABA**	1.05 (0.88–1.27)	0.66 (0.54–0.83)	1				
**ICS/LABA**	1.59 (1.36–1.91)	1.01 (0.84–1.24)	1.52 (1.29–1.79)	1			
**LAMA**	0.98 (0.85–1.13)	0.62 (0.5–0.77)	0.93 (0.79–1.08)	0.61 (0.52–0.72)	1		
**LABA**	1.01 (0.87–1.18)	0.64 (0.51–0.8)	0.96 (0.81–1.13)	0.63 (0.55–0.72)	1.03 (0.89–1.2)	1	
**ICS**	1.39 (1.15–1.7)	0.88 (0.68–1.15)	1.32 (1.05–1.65)	0.87 (0.72–1.05)	1.42 (1.15–1.76)	1.38 (1.15–1.67)	1

Median OR and 95% CrI were calculated as a row to column ratio.

**Abbreviations:** CrI, credible interval; ICS, inhaled corticosteroid; LABA, long-acting beta-agonist; LAMA, long-acting muscarinic antagonist; MACE, major adverse cardiovascular event; NMA, network meta-analysis; OR, odds ratio

The sensitivity analyses, including those of studies involving only patients with a low FEV1, a previous history of exacerbation, or more symptoms, showed similar results (S4 Table). In the analysis of the studies conducted for ≥24 weeks or ≥48 weeks, ICS/LAMA/LABA had a significantly higher probability of reducing the total exacerbations than any other drug class. In all analyses, ICS/LAMA/LABA showed the highest posterior probability for reducing the total exacerbations, followed by LAMA/LABA.

In the network meta-regression analysis, only the mean post-bronchodilator FEV1% of predicted showed a significant association with the total exacerbations (regression coefficient [95% CrI] = −0.03 [−0.05 to 0.003], P[beta < 0] = 0.990) (S5 Table). Even when the predicted post-bronchodilator FEV1% was adjusted, the results were similar (S6 Table).

### Moderate to severe exacerbations

The effectiveness in reducing moderate to severe exacerbations of COPD was evaluated in 67 RCTs involving 116,131 patients (Fig 4). Compared with placebo, all drug classes, except for ICS, showed significant benefits for moderate to severe exacerbations (Table 2). ICS/LAMA/LABA had a higher probability of reducing moderate to severe exacerbations than the other drug classes and placebo (S3 Table and S1 Fig). LAMA/LABA, ICS/LABA, and LAMA had a higher probability of reducing moderate to severe exacerbations than LABA or ICS.

In the sensitivity analyses, ICS/LAMA/LABA showed the highest posterior probability of reducing moderate to severe exacerbations (S7 Table). The ORs of moderate to severe exacerbations in ICS/LAMA/LABA further decreased in the patients with FEV1 ≤ 60% and a previous history of exacerbations. ICS/LAMA/LABA was the only treatment better than placebo in reducing moderate to severe exacerbations in patients with a previous exacerbation history.

In the network meta-regression analyses, no significant relationship was found (S8 Table).

### All-cause mortality

The probability of all-cause mortality was evaluated in 190 RCTs involving 221,451 patients (Fig 4). Compared with the patients who received a placebo, the patients who received ICS/LAMA/LABA and ICS/LABA showed a significantly higher probability of reduced mortality (Table 2, S3 Table, and S1 Fig). ICS/LAMA/LABA was associated with a significantly higher probability of reduced mortality than all other drug classes, except for ICS/LABA.

The sensitivity analyses, including those of studies involving only patients with a low FEV1, a previous history of exacerbation, or more symptoms, showed similar results. In the sensitivity analysis of the studies conducted for ≥24 weeks or ≥48 weeks, ICS/LAMA/LABA had a significantly higher probability of reducing the total exacerbations than any other drug class, except for ICS/LABA. ICS/LAMA/LABA showed the highest posterior probability of reducing mortality in most other analyses (S9 Table).

In the network meta-regression analyses, the mean post-bronchodilator FEV1% of predicted, percentage of patients with an exacerbation history during the past year, mean mMRC scale score, and reversibility were significantly associated with all-cause mortality (S10 Table). Even after adjusting the predicted post-bronchodilator FEV1%, the proportion of exacerbation history during the past year, and reversibility, ICS/LAMA/LABA remained the most effective treatment in reducing all-cause mortality in SUCRA (S11 Table).

### Cardiovascular-disease–related mortality

The probability of cardiovascular-disease–related mortality was evaluated in 109 RCTs involving 142,622 participants (Fig 4). ICS/LAMA/LABA and ICS/LABA did not significantly reduced cardiovascular-disease–related mortality compared to LAMA or placebo (Table 2 and S3 Table). In the meta-regression analyses, we found a significant positive relationship between the risk of total exacerbation and the risk of cardiovascular-disease–related mortality in the comparisons between ICS/LABA and placebo (regression coefficient = 1.51; 95% CrI 0.64–2.36; *P* = 0.040) (S2 Fig). There was no direct comparison of cardiovascular-disease–related mortality between ICS/LAMA/LABA and placebo.

### MACE

The probability of MACE was evaluated in 26 RCTs involving 52,881 patients (Fig 4). None of the inhaled therapies significantly increased the MACE risk (Table 2 and S3 Table).

### Pneumonia

The probability of pneumonia was evaluated in 147 RCTs involving 195,628 patients (Fig 4). Compared with LAMA/LABA, LAMA, LABA, and a placebo, ICS/LAMA/LABA, ICS/LABA, and ICS were associated with significantly higher probabilities of pneumonia (Table 2 and S3 Table).

## Discussion

We conducted the present NMA using Bayesian statistics to compare drugs and drug combinations in their efficacy to reduce exacerbations and mortality and in their safety to minimize adverse events. This NMA found that ICS/LAMA/LABA was the most effective treatment in reducing total exacerbations and all-cause mortality compared with other regular inhaled therapies in patients with stable COPD. LAMA/LABA was the second most efficacious drug class in reducing the exacerbation risk, and ICS/LABA was the second most effective drug in decreasing the mortality risk. The sensitivity analyses, including those of studies conducted for ≥24 weeks and those conducted for ≥48 weeks, showed similar results. Corticosteroid-containing therapy, including ICS/LABA/LAMA, increased the risk of pneumonia. The posterior effect size estimated by the comparison in the NMA was consistent with that revealed by the direct comparison approach (S3 Table). In the evaluation of the level of inconsistency, almost all results satisfied the consistency assumption.

To the best of our knowledge, this study is the first to find an effect of mortality reduction by pharmacologic therapy for stable COPD using Bayesian NMA. Mortality is the most important outcome in various acute and chronic diseases, including COPD. A recent pairwise meta-analysis including only 21 RCTs did not reveal a significant mortality reduction with ICS/LAMA/LABA compared with ICS/LABA, LAMA/LABA, or LAMA [4]. Although previous pairwise meta-analyses found that ICS/LAMA/LABA had benefits in reducing lung function decline and preventing acute exacerbations compared with LAMA/LABA [27] and ICS/LABA [28], mortality was not evaluated. The significant benefit of reducing all-cause mortality in the inhaled drug classes was not elucidated in recent NMAs [5–7]. Our study including all available 219 RCTs found that ICS/LAMA/LABA reduced all-cause mortality compared with the other inhaled therapies, except for ICS/LABA. We also found that ICS/LABA reduced all-cause mortality compared with placebo.

The all-cause mortality reduction by ICS/LAMA/LABA or ICS/LABA may be due to the reduction in total exacerbations. The major causes of death among COPD patients are respiratory, cardiovascular, and cancer related [8,29]. COPD treatments can have both beneficial and harmful effects on COPD-related outcomes and other comorbidity-related outcomes. Drugs that reduce acute exacerbations can also decrease the risk of respiratory death [30] and the risk of cardiovascular-disease–related death given that COPD exacerbations increase the risk of cardiovascular disease and stroke [31–34]. Exacerbations are major determinants of patients’ health condition and strong predictors of mortality [35,36] and have been considered the main efficacy outcome in RCTs [1–3]. In our study, ICS/LAMA/LABA had higher probabilities of decreasing the risk of total exacerbations and moderate to severe exacerbations than the other drug classes. Meanwhile, ICS/LAMA/LABA showed a tendency to reduce cardiovascular-disease–related death but was not statistically significant, which could be due to the lack of statistical power. In fact, only half of the included RCTs reported cardiovascular-disease–related mortality, and approximately 12% of the studies reported MACEs. In contrast, severe adverse events due to drug treatment can lead to worse survival outcomes. ICS is potentially associated with an increased pneumonia risk [8–10], and bronchodilators—including LABA and LAMA—may be related to an increase in cardiovascular risk [11,12,37]. In our NMA, there were no signals that the drugs could increase the risk of MACE, but the ICS-containing drug classes—including ICS/LAMA/LABA—had higher probabilities of increasing pneumonia. However, the risk of pneumonia was unlikely to result in an increased risk of all-cause mortality in our study. Previous studies have found that ICS does not increase pneumonia-related mortality [10,38,39]. This paradox could be explained by the fact that most patients with pneumonia in the RCTs were not severe cases [40]; additionally, ICS has a beneficial effect on pneumonia [41,42], i.e., the “double effect of ICS” [43]. This paradoxical effect could be due to the protective effects of ICS towards exacerbation, which offset its harmful effect on pneumonia [2].

We attempted to evaluate the lung cancer incidence based on the included RCTs, but it was difficult to derive a pooled outcome. Convergence for a statistic model to perform an NMA was not achieved. In most studies, the description on the lung cancer incidence was unclear and inconsistent. We think that the study period was not long enough to affect the incidence of lung cancer (study duration was less than 1 year in 64.4% of the included RCTs), and regular chest imaging (e.g., low-dose chest computed tomography [CT]) was not included in the study protocol. The problem of proving whether this is related to the intervention even if malignancy is detected remains to be solved. Another well-designed study with long-term follow-up could provide the answer to this important question.

At the expert opinion level, it has not been conclusive whether the funding source should be considered when assessing the “Other bias” domain [44,45]. After the full-text review, we could not find clear evidence that commercial sponsorship lead to a high risk of bias. In fact, there was a tendency for the studies with commercial funding sources to show a lower risk of bias. Therefore, we did not consider the funding source while assessing the “Other bias” domain.

Our study has certain strengths. First, to the best of our knowledge, this study is the largest meta-analysis comparing all eligible inhaled therapies in stable COPD patients. We extensively reviewed more than 1,400 articles concerning clinical trials, including recent large RCTs and a number of unpublished data, and included 219 RCTs in the NMA. Second, we applied the appropriate Bayesian methods to analyse rare events, such as cardiovascular-disease–related and all-cause mortality [46]. Bayesian NMA can also be used to compare treatment efficacy in the absence of head-to-head comparison studies. In addition, Bayesian NMA can provide probability statements related to one drug being better than another and probability calculations of which drugs are the best. Thus, this method can directly appeal to physicians and provide useful information to aid decision-making [13].

We acknowledge several limitations. First, we admit that several issues must be clarified before it is recommended that all COPD patients be treated with triple therapy. Although our NMA included all available RCTs, we could not conduct a subgroup analysis to identify a specific group of patients who could benefit from triple therapy more prominently. Importantly, there were few RCTs involving only less symptomatic patients or patients at a low risk. Because studies reporting information—such as eosinophil counts and chronic bronchitis—were fewer than expected, we could not generate a sufficient network for the sensitivity and meta-regression analyses. In addition, we did not evaluate the symptoms, use of rescue medication, quality of life, and lung function, which are other important outcomes. Second, NMAs such as our study combine RCTs with different study populations, inclusion criteria, and outcome measurement methods. In our NMA, COPD patients with various characteristics were included. Therefore, we performed various sensitivity analyses, which showed similar results. Among several outcome measurements, we analysed the number of participants who experienced exacerbations during the study period. This analysis was performed not only because it was the most available outcome by which to evaluate exacerbations but also because it can be derived from the time to the first exacerbation, which is another frequently measured outcome, by extracting the data from Kaplan-Meier graphs [19].

In conclusion, our Bayesian NMA suggests that triple therapy with ICS/LAMA/LABA can be the most appropriate pharmacotherapeutic option in terms of reducing the risk of exacerbations and all-cause mortality in patients with stable COPD. However, it should be considered that only a small number of studies conducted in less symptomatic patients or patients at a low risk were included in this NMA. Further studies are needed to determine whether any specific subgroup can benefit from triple therapy the most.

## Supporting information

S1 PRISMA checklist PRISMA, Preferred Reporting Items for Systematic reviews and Meta-analyses. (DOC)

S1 Text Search strategy for the SR and NMA. (DOCX)

S2 Text Detailed information regarding data synthesis and analysis. (DOCX)

S3 Text Published and unpublished studies eligible for inclusion in the SR and NMA. (DOCX)

S1 Table Risk of bias in each domain for the included studies. (DOCX)

S2 Table Assessment of publication bias in the included studies. (DOCX)

S3 Table Results of Bayesian NMAs and direct meta-analysis of total exacerbations, moderate to severe exacerbations, all-cause mortality, cardiovascular-disease–related mortality, MACEs, and pneumonia according to the drug classes. CrI, credible interval; ICS, inhaled corticosteroid; LABA, long-acting beta-agonist; LAMA, long-acting muscarinic antagonist; MACE, major adverse cardiovascular event; NMA, network meta-analysis; OR, odds ratio.
(DOCX)

S4 Table Sensitivity analyses of the drug classes to evaluate their effectiveness in reducing total exacerbations. Median OR and 95% CrI were calculated as a row to column ratio. CAT, chronic obstructive pulmonary disease assessment test; CrI, credible interval; FEV1, forced expiratory volume in 1 second; ICS, inhaled corticosteroid; LABA, long-acting beta-agonist; LAMA, long-acting muscarinic antagonist; mMRC, modified medical research council; OR, odds ratio; SUCRA, surface under the cumulative ranking curve.
(DOCX)

S5 Table Network meta-regression analysis evaluating the relationship between the covariates and total exacerbations. CrI, credible interval; FEV1, forced expiratory volume in 1 second; mMRC, modified medical research council.
(DOCX)

S6 Table NMA adjusted by the predicted post-bronchodilator FEV1% to evaluate effectiveness in reducing total exacerbations. Median OR and 95% CrI were calculated as a row to column ratio. CAT, chronic obstructive pulmonary disease assessment test; CrI, credible interval; FEV1, forced expiratory volume in 1 second; ICS, inhaled corticosteroid; LABA, long-acting beta-agonist; LAMA, long-acting muscarinic antagonist; mMRC, modified medical research council; NMA, network meta-analysis; OR, odds ratio; SUCRA, surface under the cumulative ranking curve.
(DOCX)

S7 Table Sensitivity analyses of the drug classes to evaluate their effectiveness in reducing moderate to severe exacerbations. Median OR and 95% CrI were calculated as a row to column ratio. CAT, chronic obstructive pulmonary disease assessment test; CrI, credible interval; FEV1, forced expiratory volume in 1 second; ICS, inhaled corticosteroid; LABA, long-acting beta-agonist; LAMA, long-acting muscarinic antagonist; mMRC, modified medical research council; OR, odds ratio; SUCRA, surface under the cumulative ranking curve.
(DOCX)

S8 Table Network meta-regression analysis evaluating the relationship between the covariates and moderate to severe exacerbations. CrI, credible interval; FEV1, forced expiratory volume in 1 second; mMRC, modified medical research council.
(DOCX)

S9 Table Sensitivity analyses of the drug classes to evaluate effectiveness in reducing all-cause mortality. Median OR and 95% CrI were calculated as a row to column ratio. CAT, chronic obstructive pulmonary disease assessment test; CrI, credible interval; FEV1, forced expiratory volume in 1 second; ICS, inhaled corticosteroid; LABA, long-acting beta-agonist; LAMA, long-acting muscarinic antagonist; mMRC, modified medical research council; OR, odds ratio; SUCRA, surface under the cumulative ranking curve.
(DOCX)

S10 Table Network meta-regression analysis evaluating the relationship between the covariates and all-cause mortality. CrI, credible interval; FEV1, forced expiratory volume in 1 second; mMRC, modified medical research council.
(DOCX)

S11 Table NMA adjusted by covariates evaluating effectiveness in reducing all-cause mortality. Median OR and 95% CrI were calculated as a row to column ratio. CAT, chronic obstructive pulmonary disease assessment test; CrI, credible interval; FEV1, forced expiratory volume in 1 second; ICS, inhaled corticosteroid; LABA, long-acting beta-agonist; LAMA, long-acting muscarinic antagonist; mMRC, modified medical research council; NMA, network meta-analysis; OR, odds ratio; SUCRA, surface under the cumulative ranking curve.
(DOCX)

S1 Fig Rank of each drug class evaluated by the SUCRA. The SUCRA presents the overall ranking numerically; the closer a therapy is to 100% at the higher rank, the higher the likelihood that the therapy is in the top rank. ICS, inhaled corticosteroid; LABA, long-acting beta-agonist; LAMA, long-acting muscarinic antagonist.
(TIFF)

S2 Fig The relationship between the risk of total exacerbation and the risk of all-cause or cardiovascular disease-related mortality. (TIFF)

## Abbreviations

CAT: COPD assessment test
COPD: chronic obstructive pulmonary disease
CrI: credible interval
CT: computed tomography
DAMI: Design Algorithm for Medical Literature on Intervention
EXACT: Exacerbations of Chronic Pulmonary Disease Tool
FEV1: forced expiratory volume in the first second
GOLD: Global Initiative for Chronic Obstructive Lung Disease
HCRU: health care resource use
ICS: inhaled corticosteroid
LABA: long-acting beta-agonist
LAMA: long-acting muscarinic antagonist
MACE: major adverse cardiovascular event
mMRC: modified medical research council
NMA: network meta-analysis
OR: odds ratio
P(beta < 0): posterior probability of the beta coefficient being less than 0
P(OR > 1): posterior probability of the OR exceeding 1
PRESS: Peer Review of Electronic Search Strategies
PRISMA: Preferred Reporting Items for Systematic reviews and Meta-analyses
PROSPERO: International Prospective Register of Systematic Reviews
RCT: randomized controlled trial
SR: systematic review
SUCRA: surface under the cumulative ranking curve
