# Supplementary material for: Comparisons of exacerbations and mortality among regular inhaled therapies for patients with stable chronic obstructive pulmonary disease: Systematic review and Bayesian network meta-analysis
Source: PLoS Med. 2019 Nov 15;16(11):e1002958. doi: 10.1371/journal.pmed.1002958 (PMC6857849; doi:10.1371/journal.pmed.1002958)
Supplement: S1 Text — (DOCX) [file pmed.1002958.s002.docx]

**S1 Text. Search strategy for the systematic review and network meta-analysis**

**1. MEDLINE search strategy (Search date: 2019.07.09)**

**#1. COPD patients**

"Lung Diseases, Obstructive"[Mesh:noexp] OR "Pulmonary Disease, Chronic Obstructive"[Mesh] OR "Pulmonary Emphysema"[Mesh] OR "Chronic obstructive pulmonary disease"[tiab] OR "Emphysema"[tiab] OR "Chronic bronchitis"[tiab] OR "Chronic obstructive lung disease"[tiab] OR "Obstructive lung disease"[tiab] OR "Obstructive pulmonary disease"[tiab] OR "Obstructive lung diseases"[tiab] OR "Obstructive pulmonary diseases"[tiab] OR "COPD"[tiab] **107843**

**#2. LABA (General)**

"adrenergic beta-2 receptor agonists/administration and dosage"[Mesh] OR "adrenergic beta-2 receptor agonists/adverse effects"[Mesh] OR "adrenergic beta-2 receptor agonists/pharmacology"[Mesh] OR "adrenergic beta-2 receptor agonists/therapeutic use"[Mesh] OR "adrenergic beta-2 receptor agonists/toxicity"[Mesh] OR (("long-acting"[tiab] or "long acting"[tiab] or "ultra-long acting"[tiab] or "ultra-long-acting"[tiab]) **AND** (β agonist*[tiab] OR β-agonist*[tiab] OR β2 agonist*[tiab] OR β2-agonist*[tiab] OR β-2 agonist*[tiab] OR β-2-agonist*[tiab] OR B2 agonist*[tiab] OR B2-agonist*[tiab] OR B-2 agonist*[tiab] OR B-2-agonist*[tiab] OR β(2) agonist*[tiab] OR β(2)-agonist*[tiab] OR β adrenergic agonist*[tiab] OR β-adrenergic agonist*[tiab] OR β2 adrenergic agonist*[tiab] OR β2-adrenergic agonist*[tiab] OR β-2 adrenergic agonist*[tiab] OR β-2-adrenergic agonist*[tiab] OR B2 adrenergic agonist*[tiab] OR B2-adrenergic agonist*[tiab] OR B-2 adrenergic agonist*[tiab] OR B-2-adrenergic agonist*[tiab] OR β(2) adrenergic agonist*[tiab] OR β(2)-adrenergic agonist*[tiab] OR β adrenoceptor agonist*[tiab] OR β-adrenoceptor agonist*[tiab] OR β2 adrenoceptor agonist*[tiab] OR β2-adrenoceptor agonist*[tiab] OR β-2 adrenoceptor agonist*[tiab] OR β-2-adrenoceptor agonist*[tiab] OR B2 adrenoceptor agonist*[tiab] OR B2-adrenoceptor agonist*[tiab] OR B-2 adrenoceptor agonist*[tiab] OR B-2-adrenoceptor agonist*[tiab] OR β(2) adrenoceptor agonist*[tiab] OR β(2)-adrenoceptor agonist*[tiab] OR beta agonist*[tiab] OR beta-agonist*[tiab] OR beta2 agonist*[tiab] OR beta2-agonist*[tiab] OR beta-2 agonist*[tiab] OR beta-2-agonist*[tiab] OR beta(2) agonist*[tiab] OR beta(2)-agonist*[tiab] OR beta adrenergic agonist*[tiab] OR beta-adrenergic agonist*[tiab] OR beta2 adrenergic agonist*[tiab] OR beta2-adrenergic agonist*[tiab] OR beta-2 adrenergic agonist*[tiab] OR beta-2-adrenergic agonist*[tiab] OR beta(2) adrenergic agonist*[tiab] OR beta(2)-adrenergic agonist*[tiab] OR beta adrenoceptor agonist*[tiab] OR beta-adrenoceptor agonist*[tiab] OR beta2 adrenoceptor agonist*[tiab] OR beta2-adrenoceptor agonist*[tiab] OR beta-2 adrenoceptor agonist*[tiab] OR beta-2-adrenoceptor agonist*[tiab] OR beta(2) adrenoceptor agonist*[tiab] OR beta(2)-adrenoceptor agonist*[tiab])) OR "LABA"[tiab] OR "LABAs"[tiab] OR "ultra-LABA"[tiab] OR "ultra-LABAs"[tiab] **3242**

**#3. LABA (Individual drugs)**

"Formoterol fumarate"[Mesh] OR "formoterol"[tiab] OR "formoterol-fumarate"[tiab] OR "eformoterol"[tiab] OR "Atock"[tiab] OR "Atimos"[tiab] OR "Foradil"[tiab] OR "Foradile"[tiab] OR "Oxeze"[tiab] OR "Oxis"[tiab] OR "Perforomist"[tiab] OR "BD 40A"[tiab] OR "HSDB 7287"[tiab] OR "5ZZ84GCW8B"[tiab] OR "73573-87-2"[tiab] OR "183814-30-4"[tiab] OR "Indacaterol"[Supplementary Concept] OR "Indacaterol"[tiab] OR "Indacaterol-maleate"[tiab] OR "Onbrez"[tiab] OR "Arcapta"[tiab] OR "QAB 149"[tiab] OR "QAB-149"[tiab] OR "QAB149"[tiab] OR "8OR09251MQ"[tiab] OR "312753-06-3"[tiab] OR "Olodaterol"[Supplementary Concept] OR "Olodaterol"[tiab] OR "Striverdi"[tiab] OR "BI 1744 CL"[tiab] OR "BI 1744"[tiab] OR "BI-1744"[tiab] OR "BI-1744-CL"[tiab] OR "BI1744"[tiab] OR "BI1744CL"[tiab] OR "VD2YSN1AFD"[tiab] OR "868049-49-4"[tiab] OR "Salmeterol Xinafoate"[Mesh] OR "Salmeterol"[tiab] OR "Salmeterol-xinafoate"[tiab] OR "Aeromax"[tiab] OR "Arial"[tiab] OR "Asmerole"[tiab] OR "Astmerole"[tiab] OR "Beglan"[tiab] OR "Betamican"[tiab] OR "Dilamax"[tiab] OR "Inaspir"[tiab] OR "Salmetedur"[tiab] OR "Serevent"[tiab] OR "Ultrabeta"[tiab] OR "GR 33343 X"[tiab] OR "GR 33343-X"[tiab] OR "GR 33343X"[tiab] OR "HSDB 7315"[tiab] OR "SN408D"[tiab] OR "6EW8Q962A5"[tiab] OR "2I4BC502BT"[tiab] OR " 89365-50-4"[tiab] OR " 94749-08-3"[tiab] OR "Vilanterol"[Supplementary Concept] OR ("Vilanterol"[tiab] OR "Vilanterol-trifenatate"[tiab] OR "Ellipta"[tiab] OR "GW 642444M"[tiab] OR "GW 642444x"[tiab] OR "GW-642444M"[tiab] OR "GW-642444x"[tiab] OR "GW642444M"[tiab] OR "GW642444x"[tiab] OR "028LZY775B"[tiab] OR "503068-34-6"[tiab]) **5330**

**#4. LAMA (General)**

"Muscarinic Antagonists/administration and dosage"[Mesh] OR "Muscarinic Antagonists/adverse effects"[Mesh] OR "Muscarinic Antagonists/pharmacology"[Mesh] OR "Muscarinic Antagonists/therapeutic use"[Mesh] OR "Muscarinic Antagonists/toxicity"[Mesh] OR "Cholinergic Antagonists/administration and dosage"[Mesh] OR "Cholinergic Antagonists/adverse effects"[Mesh] OR "Cholinergic Antagonists/pharmacology"[Mesh] OR "Cholinergic Antagonists/therapeutic use"[Mesh] OR "Cholinergic Antagonists/toxicity"[Mesh] OR (("long-acting"[tiab] or "long acting"[tiab] or "ultra-long acting"[tiab] or "ultra-long-acting"[tiab]) **AND** (muscarinic antagonist*[tiab] OR muscarinic receptor antagonist*[tiab] OR antimuscarinic agent*[tiab] OR anti-muscarinic agent*[tiab] OR muscarinic blocker*[tiab] OR muscarinic receptor blocker*[tiab] OR Cholinergic antagonist*[tiab] OR Cholinergic receptor antagonist*[tiab] OR anticholinergic agent*[tiab] OR anti-cholinergic agent*[tiab] OR cholinergic blocker*[tiab] OR cholinergic receptor blocker*[tiab] OR cholinolytic agent*[tiab])) OR "LAMA"[tiab] OR "LAMAs"[tiab] OR "ultra-LAMA"[tiab] OR "ultra-LAMAs"[tiab] **14880**

**#5. LAMA (Individual drugs)**

"Aclidinium bromide"[Supplementary Concept] OR "aclidinium"[tiab] OR "aclidinium-bromide"[tiab] OR "Tudorza"[tiab] OR "Eklira"[tiab] OR "Bretaris"[tiab] OR "LAS 34273"[tiab] OR "LAS W-330"[tiab] OR "UQW7UF9N91"[tiab] OR "320345-99-1"[tiab] OR "Glycopyrrolate"[Mesh] OR "Glycopyrronium"[tiab] OR "Glycopyrronium-bromide"[tiab] OR "Glycopyrrolate"[tiab] OR "Erythro-glycopyrronium"[tiab] OR "NVA237"[tiab] OR "NVA-237"[tiab] OR "Seebri"[tiab] OR "Enurev"[tiab] OR "Tovanor"[tiab] OR "DRM-04"[tiab] OR "DRM04"[tiab] OR "9SFK0PX55W "[tiab] OR "V92SO9WP2I"[tiab] OR "51186-83-5"[tiab] OR "Tiotropium Bromide"[Mesh] OR "Tiotropium"[tiab] OR "Tiotropium-bromide"[tiab] OR "Tiotropium-bromide-monohydrate"[tiab] OR "Spriva"[tiab] OR "BA 679 BR"[tiab] OR "BA-679 BR"[tiab] OR "BA 679BR"[tiab] OR "0EB439235F"[tiab] OR "XX112XZP0J"[tiab] OR "L64SXO195N"[tiab] OR "186691-13-4"[tiab] OR "136310-93-5"[tiab] OR "411207-31-3"[tiab] OR "GSK573719"[Supplementary Concept] OR "Umeclidinium"[tiab] OR "Umeclidinium-bromide"[tiab] OR "Incruse"[tiab] OR "Incruse Ellipta"[tiab] OR "GSK573719"[tiab] OR "GSK-573719"[tiab] OR "GSK573719A"[tiab] OR "7AN603V4JV"[tiab] OR "869113-09-7"[tiab] **3280**

**#6. ICS (General)**

(Inhal*[tiab]) **AND** ((("Steroids/administration and dosage"[Mesh] OR "Steroids/adverse effects"[Mesh] OR "Steroids/pharmacology"[Mesh] OR "Steroids/therapeutic use"[Mesh] OR "Steroids/toxicity"[Mesh] OR "Adrenal Cortex Hormones/administration and dosage"[Mesh] OR "Adrenal Cortex Hormones/adverse effects"[Mesh] OR "Adrenal Cortex Hormones/pharmacology"[Mesh] OR "Adrenal Cortex Hormones/therapeutic use"[Mesh] OR "Adrenal Cortex Hormones/toxicity"[Mesh]) **AND** ("Bronchodilator Agents" [Pharmacological Action] OR "Anti-Asthmatic Agents" [Pharmacological Action])) OR corticosteroid*[tiab] OR cortico-steroid*[tiab] OR glucocorticoid*[tiab] OR steroid*[tiab]) OR "ICS"[tiab] **22446**

**#7. ICS (Individual drugs)**

"Beclomethasone"[Mesh] OR "beclomethasone 17-monopropionate"[Supplementary Concept] OR "Beclomethasone"[tiab] OR "Beclometasone"[tiab] OR "Beclomethasone-17-monopropionate"[tiab] OR "Beclometasone-17-monopropionate"[tiab] OR "Beclomethasone-dipropionate"[tiab] OR "Beclometasone-dipropionate"[tiab] OR "Beclomethasone-dipropionate-monohydrate"[tiab] OR "Beclometasone-dipropionate-monohydrate"[tiab] OR "Beclodisk"[tiab] OR "Becloforte"[tiab] OR "Beclovent"[tiab] OR "Qvar"[tiab] OR "Vancenase"[tiab] OR "Vanceril"[tiab] OR "4H7L9AI22I"[tiab] OR "5B307S63B2"[tiab] OR "77011-63-3"[tiab] OR "5534-09-8"[tiab] OR "Budesonide"[Mesh] OR "Budesonide"[tiab] OR "Pulmicort"[tiab] OR "Rhinocort"[tiab] OR "Noex"[tiab] OR "Q3OKS62Q6X"[tiab] OR "51333-22-3"[tiab] OR "Fluticasone"[Mesh] OR "fluticasone propionate-17-carboxylic acid" [Supplementary Concept] OR "fluticasone furoate"[Supplementary Concept] OR Fluticason*[tiab] OR "Fluticasone-propionate"[tiab] OR "Fluticasone-furoate"[tiab] OR "CCI 18781"[tiab] OR "GW 685698X"[tiab] OR "GSK 685698"[tiab] OR "Flovent"[tiab] OR "Arnuity"[tiab] OR "O2GMZ0LF5W"[tiab] OR "JS86977WNV"[tiab] OR "80474-14-2"[tiab] OR "397864-44-7"[tiab] OR "Mometasone Furoate"[Mesh]) OR Mometason*[tiab] OR "Mometasone-furoate"[tiab] OR "Mometasone-furoate-monohydrate"[tiab] OR "Sch 32088"[tiab] OR "Asmanex"[tiab] OR "Ecural"[tiab] OR "Elocom"[tiab] OR "Elomet"[tiab] OR "Eziwin"[tiab] OR "MometAid"[tiab] OR "Novasone"[tiab] OR "8HR4QJ6DW8"[tiab] OR "105102-22-5"[tiab] OR "Triamcinolone"[Mesh] OR "Triamcinolone acetonide"[Mesh] OR "Triamcinolone"[tiab] OR "Azmacort"[tiab] OR "F446C597KA"[tiab] OR "1ZK20VI6TY"[tiab] OR "76-25-5"[tiab] OR "124-94-7"[tiab] **24241**

**#8. LABA/LAMA (Mixed drugs)**

"Duaklir"[tiab] OR "Brimica"[tiab] OR "870244-45-4"[tiab] OR "PT003"[tiab] OR "Bevespi"[tiab] OR "Bevespi Aerosphere"[tiab] OR "Duova"[tiab] OR "Tioform"[tiab] OR "QVA149"[Supplementary Concept] OR "QVA149"[tiab] OR "Ultibro"[tiab] OR "Ultibro breezhaler"[tiab] OR "Ultibron"[tiab] OR "Ultibron neohaler"[tiab] "Tiotropium-olodaterol" [Supplementary Concept] OR "Stiolto"[tiab] OR "Spiolto"[tiab] OR "Vahelva"[tiab] OR "Anoro ellipta"[tiab] **20**

**#9. ICS/LABA (Mixed drugs)**

"Fostair"[tiab] OR "Fluticasone Propionate, Salmeterol Xinafoate Drug Combination"[Mesh] OR "Advair"[tiab] OR "Seretide"[tiab] OR "Seroflo"[tiab] OR "fluticasone furoate-vilanterol trifenatate" [Supplementary Concept] OR "Breo Ellipta"[tiab] OR "Relvar Ellipta"[tiab] OR "flutiform" [Supplementary Concept] OR "Flutiform"[tiab] OR "Budesonide, Formoterol Fumarate Drug Combination"[Mesh] OR "Symbicort"[tiab] OR "Turbuhaler"[tiab] OR "Mometasone Furoate, Formoterol Fumarate Drug Combination"[Mesh] OR "Zenhale"[tiab] OR "Dulera"[tiab] OR "QMF 149"[tiab] **1016**

**#10. ICS/LABA/LAMA (Mixed drugs)**

"Trimbow"[tiab] **2**

**#11. RCT**

(((((((groups[tiab]) OR (trial[tiab])) OR (randomly[tiab])) OR (drug therapy[sh])) OR (placebo[tiab])) OR (randomized[tiab])) OR (controlled clinical trial[pt])) OR (randomized controlled trial[pt])

**#12. Human**

NOT (animals[Mesh] NOT (humans[Mesh] AND animals[Mesh]))

**#13. English**

English[Language]

**#1 AND (#2 OR #3 OR #4 OR #5 OR #6 OR #7 OR #8 OR #9 OR #10) AND #11 AND #12 AND #13 4237**

**EMBASE search strategy (Search date: 2019.07.09)**

**#1. COPD patients**

'Chronic obstructive lung disease'/exp OR 'Chronic bronchitis'/exp OR 'Lung emphysema'/exp OR 'Chronic obstructive pulmonary disease':ab,ti OR 'Emphysema':ab,ti OR 'Chronic bronchitis':ab,ti OR 'Chronic obstructive lung disease':ab,ti OR 'Obstructive lung disease':ab,ti OR 'Obstructive pulmonary disease':ab,ti OR 'Obstructive lung diseases':ab,ti OR 'Obstructive pulmonary diseases':ab,ti OR 'COPD':ab,ti **193465**

**#2. LABA (General)**

'beta 2 adrenergic receptor stimulating agent'/exp OR (('long-acting':ab,ti OR 'long acting':ab,ti OR 'ultra-long acting':ab,ti OR 'ultra-long-acting':ab,ti) **AND** ('β agonist*':ab,ti OR 'β-agonist*':ab,ti OR 'β2 agonist*':ab,ti OR 'β2-agonist*':ab,ti OR 'β-2 agonist*':ab,ti OR 'β-2-agonist*':ab,ti OR 'B2 agonist*':ab,ti OR 'B2-agonist*':ab,ti OR 'B-2 agonist*':ab,ti OR 'B-2-agonist*':ab,ti OR 'β(2) agonist*':ab,ti OR 'β(2)-agonist*':ab,ti OR 'β adrenergic agonist*':ab,ti OR 'β-adrenergic agonist*':ab,ti OR 'β2 adrenergic agonist*':ab,ti OR 'β2-adrenergic agonist*':ab,ti OR 'β-2 adrenergic agonist*':ab,ti OR 'β-2-adrenergic agonist*':ab,ti OR 'B2 adrenergic agonist*':ab,ti OR 'B2-adrenergic agonist*':ab,ti OR 'B-2 adrenergic agonist*':ab,ti OR 'B-2-adrenergic agonist*':ab,ti OR 'β(2) adrenergic agonist*':ab,ti OR 'β(2)-adrenergic agonist*':ab,ti OR 'β adrenoceptor agonist*':ab,ti OR 'β-adrenoceptor agonist*':ab,ti OR 'β2 adrenoceptor agonist*':ab,ti OR 'β2-adrenoceptor agonist*':ab,ti OR 'β-2 adrenoceptor agonist*':ab,ti OR 'β-2-adrenoceptor agonist*':ab,ti OR 'B2 adrenoceptor agonist*':ab,ti OR 'B2-adrenoceptor agonist*':ab,ti OR 'B-2 adrenoceptor agonist*':ab,ti OR 'B-2-adrenoceptor agonist*':ab,ti OR 'β(2) adrenoceptor agonist*':ab,ti OR 'β(2)-adrenoceptor agonist*':ab,ti OR 'beta agonist*':ab,ti OR 'beta-agonist*':ab,ti OR 'beta2 agonist*':ab,ti OR 'beta2-agonist*':ab,ti OR 'beta-2 agonist*':ab,ti OR 'beta-2-agonist*':ab,ti OR 'beta(2) agonist*':ab,ti OR 'beta(2)-agonist*':ab,ti OR 'beta adrenergic agonist*':ab,ti OR 'beta-adrenergic agonist*':ab,ti OR 'beta2 adrenergic agonist*':ab,ti OR 'beta2-adrenergic agonist*':ab,ti OR 'beta-2 adrenergic agonist*':ab,ti OR 'beta-2-adrenergic agonist*':ab,ti OR 'beta(2) adrenergic agonist*':ab,ti OR 'beta(2)-adrenergic agonist*':ab,ti OR 'beta adrenoceptor agonist*':ab,ti OR 'beta-adrenoceptor agonist*':ab,ti OR 'beta2 adrenoceptor agonist*':ab,ti OR 'beta2-adrenoceptor agonist*':ab,ti OR 'beta-2 adrenoceptor agonist*':ab,ti OR 'beta-2-adrenoceptor agonist*':ab,ti OR 'beta(2) adrenoceptor agonist*':ab,ti OR 'beta(2)-adrenoceptor agonist*':ab,ti)) OR 'LABA':ab,ti OR 'LABAs':ab,ti OR 'ultra-LABA':ab,ti OR 'ultra-LABAs':ab,ti **84833**

**#3. LABA (Individual drugs)**

'formoterol fumarate'/exp OR 'formoterol':ab,ti OR 'formoterol-fumarate':ab,ti OR 'eformoterol':ab,ti OR 'Atock':ab,ti OR 'Atimos':ab,ti OR 'Foradil':ab,ti OR 'Foradile':ab,ti OR 'Oxeze':ab,ti OR 'Oxis':ab,ti OR 'Perforomist':ab,ti OR 'BD 40A':ab,ti OR 'HSDB 7287':ab,ti OR '5ZZ84GCW8B':ab,ti OR '73573-87-2':ab,ti OR '183814-30-4':ab,ti OR 'indacaterol'/exp OR 'Indacaterol':ab,ti OR 'Indacaterol-maleate':ab,ti OR 'Onbrez':ab,ti OR 'Arcapta':ab,ti OR 'QAB 149':ab,ti OR 'QAB-149':ab,ti OR 'QAB149':ab,ti OR '8OR09251MQ':ab,ti OR '312753-06-3':ab,ti OR 'olodaterol'/exp OR 'Olodaterol':ab,ti OR 'Striverdi':ab,ti OR 'BI 1744 CL':ab,ti OR 'BI 1744':ab,ti OR 'BI-1744':ab,ti OR 'BI-1744-CL':ab,ti OR 'BI1744':ab,ti OR 'BI1744CL':ab,ti OR 'VD2YSN1AFD':ab,ti OR '868049-49-4':ab,ti OR 'salmeterol xinafoate'/exp OR 'Salmeterol':ab,ti OR 'Salmeterol-xinafoate':ab,ti OR 'Aeromax':ab,ti OR 'Arial':ab,ti OR 'Asmerole':ab,ti OR 'Astmerole':ab,ti OR 'Beglan':ab,ti OR 'Betamican':ab,ti OR 'Dilamax':ab,ti OR 'Inaspir':ab,ti OR 'Salmetedur':ab,ti OR 'Serevent':ab,ti OR 'Ultrabeta':ab,ti OR 'GR 33343 X':ab,ti OR 'GR 33343-X':ab,ti OR 'GR 33343X':ab,ti OR 'HSDB 7315':ab,ti OR 'SN408D':ab,ti OR '6EW8Q962A5':ab,ti OR '2I4BC502BT':ab,ti OR '89365-50-4':ab,ti OR '94749-08-3':ab,ti OR 'vilanterol'/exp OR 'vilanterol trifenatate'/exp OR 'Vilanterol':ab,ti OR 'Vilanterol-trifenatate':ab,ti OR 'Ellipta':ab,ti OR 'GW 642444M':ab,ti OR 'GW 642444x':ab,ti OR 'GW-642444M':ab,ti OR 'GW-642444x':ab,ti OR 'GW642444M':ab,ti OR 'GW642444x':ab,ti OR '028LZY775B':ab,ti OR '503068-34-6':ab,ti **9946**

**#4. LAMA (General)**

'cholinergic receptor blocking agent'/exp OR 'muscarinic receptor blocking agent'/exp OR (('long-acting':ab,ti or 'long acting':ab,ti or 'ultra-long acting':ab,ti or 'ultra-long-acting':ab,ti) **AND** ('muscarinic antagonist*':ab,ti OR 'muscarinic receptor antagonist*':ab,ti OR 'antimuscarinic agent*':ab,ti OR 'anti-muscarinic agent*':ab,ti OR 'muscarinic blocker*':ab,ti OR 'muscarinic receptor blocker*':ab,ti OR 'Cholinergic antagonist*':ab,ti OR 'Cholinergic receptor antagonist*':ab,ti OR 'anticholinergic agent*':ab,ti OR 'anti-cholinergic agent*':ab,ti OR 'cholinergic blocker*':ab,ti OR 'cholinergic receptor blocker*':ab,ti OR 'cholinolytic agent*':ab,ti)) OR 'LAMA':ab,ti OR 'LAMAs':ab,ti OR 'ultra-LAMA':ab,ti OR 'ultra-LAMAs':ab,ti **232961**

**#5. LAMA (Individual drugs)**

'aclidinium bromide'/exp OR 'aclidinium':ab,ti OR 'aclidinium-bromide':ab,ti OR 'Tudorza':ab,ti OR 'Eklira':ab,ti OR 'Bretaris':ab,ti OR 'LAS 34273':ab,ti OR 'LAS W-330':ab,ti OR 'UQW7UF9N91':ab,ti OR '320345-99-1':ab,ti OR 'glycopyrronium'/exp OR 'Glycopyrronium':ab,ti OR 'Glycopyrronium-bromide':ab,ti OR 'Glycopyrrolate':ab,ti OR 'Erythro-glycopyrronium':ab,ti OR 'NVA237':ab,ti OR 'Seebri':ab,ti OR 'Enurev':ab,ti OR 'Tovanor':ab,ti OR 'DRM-04':ab,ti OR 'DRM04':ab,ti OR '9SFK0PX55W':ab,ti OR 'V92SO9WP2I':ab,ti OR '51186-83-5':ab,ti OR 'tiotropium bromide'/exp OR 'Tiotropium':ab,ti OR 'Tiotropium-bromide':ab,ti OR 'Tiotropium-bromide-monohydrate':ab,ti OR 'Spriva':ab,ti OR 'BA 679 BR':ab,ti OR 'BA-679 BR':ab,ti OR 'BA 679BR':ab,ti OR '0EB439235F':ab,ti OR 'XX112XZP0J':ab,ti OR 'L64SXO195N':ab,ti OR '186691-13-4':ab,ti OR '136310-93-5':ab,ti OR '411207-31-3':ab,ti OR 'umeclidinium'/exp OR 'Umeclidinium-bromide':ab,ti OR 'Incruse':ab,ti OR 'Incruse Ellipta':ab,ti OR 'GSK573719':ab,ti OR 'GSK-573719':ab,ti OR 'GSK573719A':ab,ti OR '7AN603V4JV':ab,ti OR '869113-09-7':ab,ti **12623**

**#6. LABA/LAMA (Mixed drugs)**

'aclidinium bromide plus formoterol fumarate'/exp OR 'Duaklir':ab,ti OR 'Brimica':ab,ti OR '870244-45-4':ab,ti OR 'formoterol fumarate plus glycopyrronium bromide'/exp OR 'PT003':ab,ti OR 'Bevespi':ab,ti OR 'Bevespi Aerosphere':ab,ti OR 'Duova':ab,ti OR 'Tioform':ab,ti OR 'glycopyrronium bromide plus indacaterol'/exp OR 'QVA149':ab,ti OR 'Ultibro':ab,ti OR 'Ultibro breezhaler':ab,ti OR 'Ultibron':ab,ti OR 'Ultibron neohaler':ab,ti OR 'olodaterol plus tiotropium bromide'/exp OR 'Stiolto':ab,ti OR 'Spiolto':ab,ti OR 'Vahelva':ab,ti OR 'umeclidinium plus vilanterol'/exp OR 'Anoro ellipta':ab,ti **873**

**#7. ICS (General)**

(('inhal*':ab,ti) **AND** ('glucocorticoid'/exp OR 'corticosteroid':ab,ti OR 'cortico-steroid':ab,ti OR 'glucocorticoid*':ab,ti OR 'steroid*':ab,ti)) OR 'ICS':ab,ti **36724**

**#8. ICS (Individual drugs)**

'beclometasone'/exp OR 'beclometasone dipropionate'/exp OR 'Beclomethasone':ab,ti OR 'Beclometasone':ab,ti OR 'Beclomethasone-17-monopropionate':ab,ti OR 'Beclometasone-17-monopropionate':ab,ti OR 'Beclomethasone-dipropionate':ab,ti OR 'Beclometasone-dipropionate':ab,ti OR 'Beclomethasone-dipropionate-monohydrate':ab,ti OR 'Beclometasone-dipropionate-monohydrate':ab,ti OR 'Beclodisk':ab,ti OR 'Becloforte':ab,ti OR 'Beconase':ab,ti OR 'Beclovent':ab,ti OR 'Qvar':ab,ti OR 'Vancenase':ab,ti OR 'Vanceril':ab,ti OR '4H7L9AI22I':ab,ti OR '5B307S63B2':ab,ti OR '77011-63-3':ab,ti OR '5534-09-8':ab,ti OR 'budesonide'/exp OR 'Budesonide':ab,ti OR 'Pulmicort':ab,ti OR 'Rhinocort':ab,ti OR 'Noex':ab,ti OR 'Q3OKS62Q6X':ab,ti OR '51333-22-3':ab,ti OR 'fluticasone'/exp OR 'fluticasone furoate'/exp OR 'fluticasone propionate'/exp OR 'Fluticason*':ab,ti OR 'Fluticasone-propionate':ab,ti OR 'Fluticasone-furoate':ab,ti OR 'CCI 18781':ab,ti OR 'GW 685698X':ab,ti OR 'GSK 685698':ab,ti OR 'Flovent':ab,ti OR 'Arnuity':ab,ti OR 'O2GMZ0LF5W':ab,ti OR 'JS86977WNV':ab,ti OR '80474-14-2':ab,ti OR '397864-44-7':ab,ti OR 'mometasone furoate'/exp OR 'Mometason*':ab,ti OR 'Mometasone-furoate':ab,ti OR 'Mometasone-furoate-monohydrate':ab,ti OR 'Sch 32088':ab,ti OR 'Asmanex':ab,ti OR 'Ecural':ab,ti OR 'Elocom':ab,ti OR 'Elomet':ab,ti OR 'Eziwin':ab,ti OR 'MometAid':ab,ti OR 'Novasone':ab,ti OR '8HR4QJ6DW8':ab,ti OR '105102-22-5':ab,ti OR 'Triamcinolone'/exp OR 'Triamcinolone acetonide'/exp OR 'Triamcinolone':ab,ti OR 'Azmacort':ab,ti OR 'F446C597KA':ab,ti OR '1ZK20VI6TY':ab,ti OR '76-25-5':ab,ti OR '124-94-7':ab,ti **67939**

**#9. ICS/LABA (Mixed drugs)**

'beclometasone dipropionate plus formoterol fumarate'/exp OR 'Fostair':ab,ti OR 'fluticasone propionate plus salmeterol'/exp OR 'fluticasone propionate plus salmeterol xinafoate'/exp OR 'Advair':ab,ti OR 'Seretide':ab,ti OR 'Seroflo':ab,ti OR 'fluticasone furoate plus vilanterol'/exp OR 'Breo Ellipta':ab,ti OR 'Relvar Ellipta':ab,ti OR 'fluticasone propionate plus formoterol fumarate'/exp OR 'Flutiform':ab,ti OR 'budesonide plus formoterol'/exp OR 'Symbicort':ab,ti OR 'Turbuhaler':ab,ti OR 'formoterol fumarate plus mometasone furoate'/exp OR 'Zenhale':ab,ti OR 'Dulera':ab,ti OR 'indacaterol plus mometasone furoate'/exp OR 'QMF 149':ab,ti **6067**

**#10. RCT**

'crossover procedure'/exp OR 'crossover procedure':ab,ti OR 'double blind procedure'/exp OR 'double blind procedure':ab,ti OR 'randomized controlled trial'/exp OR 'randomized controlled trial':ab,ti OR 'single blind procedure'/exp OR 'single blind procedure':ab,ti OR 'random*':ab,ti OR 'factorial*':ab,ti OR 'crossover*':ab,ti OR 'cross over':ab,ti OR 'cross-over':ab,ti OR 'placebo*':ab,ti OR ('doubl*':ab,ti AND 'blind*':ab,ti) OR ('singl*':ab,ti AND 'blind*':ab,ti) OR 'assign*':ab,ti OR 'allocat*':ab,ti OR 'volunteer*':ab,ti

**#11. Human**

NOT ('animal'/exp NOT ('human'/exp AND 'animal'/exp))

**#12. English**

[english]/lim

**#1 AND (#2 OR #3 OR #4 OR #5 OR #6 OR #7 OR #8 OR #9) AND #10 AND #11 AND #12 4964**

**Cochrane Library search strategy (Search date: 2019.07.09)**

**#1. COPD patients**

MeSH descriptor: [Pulmonary Disease, Chronic Obstructive] explode all trees

OR

Chronic obstructive pulmonary disease:ti,ab,kw OR Emphysema:ti,ab,kw OR Chronic bronchitis:ti,ab,kw OR Chronic obstructive lung disease:ti,ab,kw OR Obstructive lung disease:ti,ab,kw OR Obstructive pulmonary disease:ti,ab,kw OR Obstructive lung diseases:ti,ab,kw OR Obstructive pulmonary diseases:ti,ab,kw OR COPD:ti,ab,kw **22161**

**#2. LABA (General)**

MeSH descriptor: [Adrenergic beta-2 Receptor Agonists] explode all trees

OR

((long-acting:ti,ab,kw OR long acting:ti,ab,kw OR ultra-long acting:ti,ab,kw OR ultra-long-acting:ti,ab,kw) **AND** (β agonist*:ti,ab,kw OR β-agonist*:ti,ab,kw OR β2 agonist*:ti,ab,kw OR β-2 agonist*:ti,ab,kw OR B2 agonist*:ti,ab,kw OR B-2 agonist*:ti,ab,kw OR β(2) agonist*:ti,ab,kw OR β adrenergic agonist*:ti,ab,kw OR β-adrenergic agonist*:ti,ab,kw OR β2 adrenergic agonist*:ti,ab,kw OR β-2 adrenergic agonist*:ti,ab,kw OR B2 adrenergic agonist*:ti,ab,kw OR B-2 adrenergic agonist*:ti,ab,kw OR β(2) adrenergic agonist*:ti,ab,kw OR β adrenoceptor agonist*:ti,ab,kw OR β-adrenoceptor agonist*:ti,ab,kw OR β2 adrenoceptor agonist*:ti,ab,kw OR β-2 adrenoceptor agonist*:ti,ab,kw OR B2 adrenoceptor agonist*:ti,ab,kw OR B-2 adrenoceptor agonist*:ti,ab,kw OR β(2) adrenoceptor agonist*:ti,ab,kw OR beta agonist*:ti,ab,kw OR beta-agonist*:ti,ab,kw OR beta2 agonist*:ti,ab,kw OR beta-2 agonist*:ti,ab,kw OR beta(2) agonist*:ti,ab,kw OR beta adrenergic agonist*:ti,ab,kw OR beta-adrenergic agonist*:ti,ab,kw OR beta2 adrenergic agonist*:ti,ab,kw OR beta-2 adrenergic agonist*:ti,ab,kw OR beta(2) adrenergic agonist*:ti,ab,kw OR beta adrenoceptor agonist*:ti,ab,kw OR beta-adrenoceptor agonist*:ti,ab,kw OR beta2 adrenoceptor agonist*:ti,ab,kw OR beta-2 adrenoceptor agonist*:ti,ab,kw OR beta(2) adrenoceptor agonist*:ti,ab,kw)) OR LABA:ti,ab,kw OR LABAs:ti,ab,kw OR ultra-LABA:ti,ab,kw OR ultra-LABAs:ti,ab,kw **3502**

**#3. LABA (Individual drugs)**

MeSH descriptor: [Formoterol Fumarate] explode all trees OR MeSH descriptor: [Salmeterol Xinafoate] explode all trees

OR

formoterol:ti,ab,kw OR formoterol-fumarate:ti,ab,kw OR eformoterol:ti,ab,kw OR Atock:ti,ab,kw OR Atimos:ti,ab,kw OR Foradil:ti,ab,kw OR Foradile:ti,ab,kw OR Oxeze:ti,ab,kw OR Oxis:ti,ab,kw OR Perforomist:ti,ab,kw OR BD 40A:ti,ab,kw OR HSDB 7287:ti,ab,kw OR 5ZZ84GCW8B:ti,ab,kw OR Indacaterol:ti,ab,kw OR Indacaterol-maleate:ti,ab,kw OR Onbrez:ti,ab,kw OR Arcapta:ti,ab,kw OR QAB 149:ti,ab,kw OR QAB-149:ti,ab,kw OR QAB149:ti,ab,kw OR 8OR09251MQ:ti,ab,kw OR Olodaterol:ti,ab,kw OR Striverdi:ti,ab,kw OR BI 1744 CL:ti,ab,kw OR BI 1744:ti,ab,kw OR BI-1744:ti,ab,kw OR BI1744:ti,ab,kw OR BI1744CL:ti,ab,kw OR VD2YSN1AFD:ti,ab,kw OR Salmeterol:ti,ab,kw OR Salmeterol-xinafoate:ti,ab,kw OR Aeromax:ti,ab,kw OR Arial:ti,ab,kw OR Asmerole:ti,ab,kw OR Astmerole:ti,ab,kw OR Beglan:ti,ab,kw OR Betamican:ti,ab,kw OR Dilamax:ti,ab,kw OR Inaspir:ti,ab,kw OR Salmetedur:ti,ab,kw OR Serevent:ti,ab,kw OR Ultrabeta:ti,ab,kw OR GR 33343 X:ti,ab,kw OR GR 33343X:ti,ab,kw OR HSDB 7315:ti,ab,kw OR SN408D:ti,ab,kw OR 6EW8Q962A5:ti,ab,kw OR 2I4BC502BT:ti,ab,kw OR Vilanterol:ti,ab,kw OR Vilanterol-trifenatate:ti,ab,kw OR Ellipta:ti,ab,kw OR GW 642444M:ti,ab,kw OR GW 642444x:ti,ab,kw OR GW-642444M:ti,ab,kw OR GW-642444x:ti,ab,kw OR GW642444M:ti,ab,kw OR GW642444x:ti,ab,kw OR 028LZY775B:ti,ab,kw **6911**

**#4. LAMA (General)**

MeSH descriptor: [Muscarinic Antagonists] explode all trees OR MeSH descriptor: [Cholinergic Antagonists] explode all trees

OR

((long-acting:ti,ab,kw or long acting:ti,ab,kw or ultra-long acting:ti,ab,kw or ultra-long-acting:ti,ab,kw) **AND** (muscarinic antagonist*:ti,ab,kw OR muscarinic receptor antagonist*:ti,ab,kw OR antimuscarinic agent*:ti,ab,kw OR anti-muscarinic agent*:ti,ab,kw OR muscarinic blocker*:ti,ab,kw OR muscarinic receptor blocker*:ti,ab,kw OR Cholinergic antagonist*:ti,ab,kw OR Cholinergic receptor antagonist*:ti,ab,kw OR anticholinergic agent*:ti,ab,kw OR anti-cholinergic agent*:ti,ab,kw OR cholinergic blocker*:ti,ab,kw OR cholinergic receptor blocker*:ti,ab,kw OR cholinolytic agent*:ti,ab,kw)) OR LAMA:ti,ab,kw OR LAMAs:ti,ab,kw OR ultra-LAMA:ti,ab,kw OR ultra-LAMAs:ti,ab,kw **2267**

**#5. LAMA (Individual drugs)**

MeSH descriptor: [Glycopyrrolate] explode all trees OR MeSH descriptor: [Tiotropium Bromide] explode all trees

OR

aclidinium:ti,ab,kw OR aclidinium-bromide:ti,ab,kw OR Tudorza:ti,ab,kw OR Eklira:ti,ab,kw OR Bretaris:ti,ab,kw OR LAS 34273:ti,ab,kw OR UQW7UF9N91:ti,ab,kw OR Glycopyrronium:ti,ab,kw OR Glycopyrronium-bromide:ti,ab,kw OR Glycopyrrolate:ti,ab,kw OR Erythro-glycopyrronium:ti,ab,kw OR NVA237:ti,ab,kw OR Seebri:ti,ab,kw OR Enurev:ti,ab,kw OR Tovanor:ti,ab,kw OR DRM-04:ti,ab,kw OR DRM04:ti,ab,kw OR 9SFK0PX55W:ti,ab,kw OR V92SO9WP2I:ti,ab,kw OR Tiotropium:ti,ab,kw OR Tiotropium-bromide:ti,ab,kw OR Tiotropium-bromide-monohydrate:ti,ab,kw OR Spriva:ti,ab,kw OR BA 679 BR:ti,ab,kw OR BA-679 BR:ti,ab,kw OR BA 679BR:ti,ab,kw OR 0EB439235F:ti,ab,kw OR XX112XZP0J:ti,ab,kw OR L64SXO195N:ti,ab,kw OR Umeclidinium-bromide:ti,ab,kw OR Incruse:ti,ab,kw OR Incruse Ellipta:ti,ab,kw OR GSK573719:ti,ab,kw OR GSK-573719:ti,ab,kw OR GSK573719A:ti,ab,kw OR 7AN603V4JV:ti,ab,kw **3676**

**#5. ICS (General)**

((inhal*:ti,ab,kw) **AND** (((MeSH descriptor: [Steroids] explode all trees OR MeSH descriptor: [Adrenal Cortex Hormones] explode all trees) **AND** (MeSH descriptor: [Bronchodilator Agents] explode all trees OR MeSH descriptor: [Anti-Asthmatic Agents] explode all trees)) OR corticosteroid:ti,ab,kw OR cortico-steroid:ti,ab,kw OR glucocorticoid*:ti,ab,kw OR steroid*:ti,ab,kw)) OR ICS:ti,ab,kw **7983**

**#6. ICS (Individual drugs)**

MeSH descriptor: [Beclomethasone] explode all trees OR MeSH descriptor: [Budesonide] explode all trees OR MeSH descriptor: [Fluticasone] explode all trees OR MeSH descriptor: [Mometasone Furoate] explode all trees OR MeSH descriptor: [Triamcinolone] explode all trees OR MeSH descriptor: [Triamcinolone acetonide] explode all trees

OR

Beclomethasone:ti,ab,kw OR Beclometasone:ti,ab,kw OR Beclomethasone-dipropionate:ti,ab,kw OR Beclometasone-dipropionate:ti,ab,kw OR Beclodisk:ti,ab,kw OR Becloforte:ti,ab,kw OR Beconase:ti,ab,kw OR Beclovent:ti,ab,kw OR Qvar:ti,ab,kw OR Vancenase:ti,ab,kw OR Vanceril:ti,ab,kw OR 4H7L9AI22I:ti,ab,kw OR 5B307S63B2:ti,ab,kw OR Budesonide:ti,ab,kw OR Pulmicort:ti,ab,kw OR Rhinocort:ti,ab,kw OR Noex:ti,ab,kw OR Q3OKS62Q6X:ti,ab,kw OR Fluticason*:ti,ab,kw OR Fluticasone-propionate:ti,ab,kw OR Fluticasone-furoate:ti,ab,kw OR CCI 18781:ti,ab,kw OR GW 685698X:ti,ab,kw OR GSK 685698:ti,ab,kw OR Flovent:ti,ab,kw OR Arnuity:ti,ab,kw OR O2GMZ0LF5W:ti,ab,kw OR JS86977WNV:ti,ab,kw OR Mometason*:ti,ab,kw OR Mometasone-furoate:ti,ab,kw OR Mometasone-furoate-monohydrate:ti,ab,kw OR Sch 32088:ti,ab,kw OR Asmanex:ti,ab,kw OR Ecural:ti,ab,kw OR Elocom:ti,ab,kw OR Elomet:ti,ab,kw OR Eziwin:ti,ab,kw OR MometAid:ti,ab,kw OR Novasone:ti,ab,kw OR 8HR4QJ6DW8:ti,ab,kw OR Triamcinolone:ti,ab,kw OR Azmacort:ti,ab,kw OR F446C597KA:ti,ab,kw OR 1ZK20VI6TY:ti,ab,kw **14329**

**#7. LABA/LAMA (Mixed drugs)**

Duaklir:ti,ab,kw OR Brimica:ti,ab,kw OR PT003:ti,ab,kw OR Bevespi:ti,ab,kw OR Bevespi Aerosphere:ti,ab,kw OR Duova:ti,ab,kw OR Tioform:ti,ab,kw OR QVA149:ti,ab,kw OR Ultibro:ti,ab,kw OR Ultibro breezhaler:ti,ab,kw OR Ultibron:ti,ab,kw OR Ultibron neohaler:ti,ab,kw OR Stiolto:ti,ab,kw OR Spiolto:ti,ab,kw OR Vahelva:ti,ab,kw OR Anoro ellipta:ti,ab,kw  **316**

**#8. ICS/LABA (Mixed drugs)**

MeSH descriptor: [Fluticasone Propionate, Salmeterol Xinafoate Drug Combination] explode all trees OR MeSH descriptor: [Budesonide, Formoterol Fumarate Drug Combination] explode all trees OR MeSH descriptor: [Mometasone Furoate, Formoterol Fumarate Drug Combination] explode all trees

OR

Fostair:ti,ab,kw OR Advair:ti,ab,kw OR Seretide:ti,ab,kw OR Seroflo:ti,ab,kw OR Breo Ellipta:ti,ab,kw OR Relvar Ellipta:ti,ab,kw OR Flutiform:ti,ab,kw OR Symbicort:ti,ab,kw OR Turbuhaler:ti,ab,kw OR Zenhale:ti,ab,kw OR Dulera:ti,ab,kw OR QMF 149:ti,ab,kw **1814**

**#9. RCT**

Select "Trials"

**#1 AND (#2 OR #3 OR #4 OR #5 OR #6 OR #7 OR #8) AND #9 5136**
