# Supplementary material for: Comparisons of exacerbations and mortality among regular inhaled therapies for patients with stable chronic obstructive pulmonary disease: Systematic review and Bayesian network meta-analysis
Source: PLoS Med. 2019 Nov 15;16(11):e1002958. doi: 10.1371/journal.pmed.1002958 (PMC6857849; doi:10.1371/journal.pmed.1002958)
Supplement: S2 Text — (DOCX) [file pmed.1002958.s003.docx]

**S2 Text.** **Detailed information regarding data synthesis and analysis**

For the present Bayesian network meta-analysis, we used a random effects model with a heterogeneous variance structure (1, 2) because we found more than two drug classes in individual studies and assumed that the variance of the log odds ratio of individual treatment compared with baseline treatment was different. The prior distributions for the parameters in the Bayesian model were assumed to be noninformative and to have normal or uniform distribution (3).

We estimated the posterior median odds ratio (OR) with 95% credible intervals (CrIs) and the posterior probability of the OR exceeding 1 (P(OR>1)) to measure the association between the inhaled drugs and treatment outcomes. If an OR greater than 1 was derived in a pairwise comparison, it indicated that the comparator group (upper side of the league table) was more beneficial than the treatment group (left side of the league table). Additionally, direct meta-analyses were conducted using the random effects model for each direct comparison, and the results are presented as ORs with 95% confidence intervals (CIs). The best treatment for reducing each outcome was determined using the relative probability of that treatment being the most effective based on the surface under the cumulative ranking curve (SUCRA) (4). In the network meta-regression analysis, regression coefficients of covariates were presented to explain the effect of each covariate on the outcome. A significant result was assumed if P(OR>1) or the posterior probability of the regression coefficient (beta) lower than 0 (P(beta<0)) was less than 0.025 or more than 0.975.

The parameters were estimated using the Markov Chain Monte Carlo (MCMC) algorithm in WinBUGS version 1.4.6 (Imperial College and Medical Research Council, UK). The convergence of the MCMC algorithm was checked using trace plots, autocorrelation plots, and Gelman-Rubin statistics. We discarded the first 10,000 iterations to eliminate the initial value effect and selected 10,000 samples after applying the appropriate thinning rate to satisfy the autocorrelation assumption in the MCMC algorithm in two chains.

We compared the baseline characteristics of the eligible trials and the demographic characteristics of patients to check the homogeneity and similarity assumptions. A network meta-regression was performed to adjust the clinical heterogeneities across studies, and the baseline lung function, previous history of exacerbation, and current symptoms were considered as covariates (5). The publication bias was investigated using funnel plots, Egger’s test, the Orwin Fail-Safe N method, and Duval-Tweedie Trim and Fill for each direct comparison. The consistency assumption that the direct estimate might be consistent with indirect estimates is another main assumption in a network meta-analysis, and this assumption was assessed using the node-splitting method.(6) Heterogeneity was assessed based on the posterior median of the standard deviation (SD) between studies. SD close to 0 indicates small heterogeneity, and SD>1 indicates substantial heterogeneity (7, 8).

Sensitivity analyses were performed at study level according to the following criteria: studies including patients with post-bronchodilator FEV1%≤60%, studies including those with post-bronchodilator FEV1%≤50%, studies including those with total exacerbations ≥1 in the past year, studies including those with total exacerbations ≥2 or severe exacerbation ≥1 in the past year, studies including those with an mMRC scale score ≥2 or a CAT score ≥10, studies with a treatment duration ≥24 weeks, and studies with a treatment duration ≥48 weeks.

In the network meta-regression analyses, we evaluated the impacts of pre-specified covariates, such as the mean post-bronchodilator FEV1% of predicted, the percentage of those with total exacerbations ≥1 in the past year, the percentage of those with total exacerbations ≥ 2 or severe exacerbation ≥1 in the past year, the percentage of eosinophils in serum, mMRC scale scores, and reversibility. For covariates that were significant in the regression model, adjusted ORs are presented. In addition, we estimated regression coefficient to evaluate the relationship between the risk of total exacerbation and the risk of all-cause or cardiovascular disease-related mortality using direct meta-regression analysis with Bayesian method.

**References**

1. Lu G, Ades AE. Combination of direct and indirect evidence in mixed treatment comparisons. Statistics in medicine. 2004;23(20):3105-24.

2. Dias S, Welton NJ, Sutton AJ, Ades AE. NICE Decision Support Unit Technical Support Documents. A Generalised Linear Modelling Framework for Pairwise and Network Meta-Analysis of Randomised Controlled Trials. London: National Institute for Health and Care Excellence (NICE)

unless otherwise stated. All rights reserved.; 2014.

3. Carlin BP, Hong H, Shamliyan TA, Sainfort F, Kane RL. AHRQ Methods for Effective Health Care. Case Study Comparing Bayesian and Frequentist Approaches for Multiple Treatment Comparisons. Rockville (MD): Agency for Healthcare Research and Quality (US); 2013.

4. Salanti G, Ades AE, Ioannidis JP. Graphical methods and numerical summaries for presenting results from multiple-treatment meta-analysis: an overview and tutorial. Journal of clinical epidemiology. 2011;64(2):163-71.

5. Dias S, Sutton AJ, Welton NJ, Ades AE. Evidence synthesis for decision making 3: heterogeneity--subgroups, meta-regression, bias, and bias-adjustment. Medical decision making : an international journal of the Society for Medical Decision Making. 2013;33(5):618-40.

6. Dias S, Welton NJ, Caldwell DM, Ades AE. Checking consistency in mixed treatment comparison meta-analysis. Statistics in medicine. 2010;29(7-8):932-44.

7. Spiegelhalter DJ, Abrams KR, Myles JP. Bayesian approaches to clinical trials and health-care evaluation: John Wiley & Sons; 2004.

8. Moran JL, Graham PL, Rockliff S, Bersten AD. Updating the evidence for the role of corticosteroids in severe sepsis and septic shock: a Bayesian meta-analytic perspective. Critical care (London, England). 2010;14(4):R134.
