# Supplementary material for: Comparisons of exacerbations and mortality among regular inhaled therapies for patients with stable chronic obstructive pulmonary disease: Systematic review and Bayesian network meta-analysis
Source: PLoS Med. 2019 Nov 15;16(11):e1002958. doi: 10.1371/journal.pmed.1002958 (PMC6857849; doi:10.1371/journal.pmed.1002958)
Supplement: S3 Text — (DOCX) [file pmed.1002958.s004.docx]

**S3 Text. Published and unpublished studies eligible for inclusion in the systematic review and network meta-analysis**

**Published studies (1-185)**

1. Aalbers R, Ayres J, Backer V, Decramer M, Lier PA, Magyar P, et al. Formoterol in patients with chronic obstructive pulmonary disease: a randomized, controlled, 3-month trial. The european respiratory journal [Internet]. 2002; 19(5):[936-43 pp.]. Available from: <http://onlinelibrary.wiley.com/o/cochrane/clcentral/articles/992/CN-00394992/frame.html>.

2. Aaron SD, Vandemheen KL, Fergusson D, Maltais F, Bourbeau J, Goldstein R, et al. Tiotropium in combination with placebo, salmeterol, or fluticasone-salmeterol for treatment of chronic obstructive pulmonary disease: a randomized trial. Annals of internal medicine. 2007;146(8):545-55.

3. Abrahams R, Moroni-Zentgraf P, Ramsdell J, Schmidt H, Joseph E, Karpel J. Safety and efficacy of the once-daily anticholinergic BEA2180 compared with tiotropium in patients with COPD. Respir Med. 2013;107(6):854-62.

4. Ambrosino N, Foglio K, Balzano G, Paggiaro PL, Lessi P, Kesten S. Tiotropium and exercise training in COPD patients: effects on dyspnea and exercise tolerance. International journal of chronic obstructive pulmonary disease [Internet]. 2008; 3(4):[771-80 pp.]. Available from: <http://onlinelibrary.wiley.com/o/cochrane/clcentral/articles/138/CN-00683138/frame.html>.

5. Anzueto A, Ferguson GT, Feldman G, Chinsky K, Seibert A, Emmett A, et al. Effect of fluticasone propionate/salmeterol (250/50) on COPD exacerbations and impact on patient outcomes. Copd [Internet]. 2009; 6(5):[320-9 pp.]. Available from: <http://onlinelibrary.wiley.com/o/cochrane/clcentral/articles/375/CN-00730375/frame.html>.

6. Asai K, Kobayashi A, Makihara Y, Johnson M. Anti-inflammatory effects of salmeterol/fluticasone propionate 50/250 mcg combination therapy in japanese patients with chronic obstructive pulmonary disease. International Journal of COPD [Internet]. 2015; 10:[803-11 pp.]. Available from: <http://onlinelibrary.wiley.com/o/cochrane/clcentral/articles/273/CN-01075273/frame.html>.

7. Barnes PJ, Pocock SJ, Magnussen H, Iqbal A, Kramer B, Higgins M, et al. Integrating indacaterol dose selection in a clinical study in COPD using an adaptive seamless design. Pulm Pharmacol Ther. 2010;23(3):165-71.

8. Bateman E, Singh D, Smith D, Disse B, Towse L, Massey D, et al. Efficacy and safety of tiotropium Respimat SMI in COPD in two 1-year randomized studies. International journal of chronic obstructive pulmonary disease. 2010;5:197-208.

9. Bateman ED, Ferguson GT, Barnes N, Gallagher N, Green Y, Henley M, et al. Dual bronchodilation with QVA149 versus single bronchodilator therapy: the SHINE study. The European respiratory journal. 2013;42(6):1484-94.

10. Bateman ED, Tashkin D, Siafakas N, Dahl R, Towse L, Massey D, et al. A one-year trial of tiotropium Respimat® plus usual therapy in COPD patients. Respiratory Medicine. 2010;104(10):1460-72.

11. Beeh KM, Beier J, Buhl R, Stark-Lorenzen P, Gerken F, Metzdorf N, et al. Efficacy of tiotropium bromide (Spiriva) in patients with chronic-obstructive pulmonary disease (COPD) of different severities. Pneumologie (stuttgart, germany) [Internet]. 2006; 60(6):[341-6 pp.]. Available from: <http://onlinelibrary.wiley.com/o/cochrane/clcentral/articles/864/CN-00556864/frame.html>.

12. Betsuyaku T, Kato M, Fujimoto K, Kobayashi A, Hayamizu T, Hitosugi H, et al. A randomized trial of symptom-based management in Japanese patients with COPD. International journal of chronic obstructive pulmonary disease. 2018;13:2409-23.

13. Bhatt SP, Dransfield MT, Cockcroft JR, Wang-Jairaj J, Midwinter DA, Rubin DB, et al. A randomized trial of once-daily fluticasone furoate/vilanterol or vilanterol versus placebo to determine effects on arterial stiffness in COPD. International journal of COPD [Internet]. 2017; 12:[351-65 pp.]. Available from: <http://onlinelibrary.wiley.com/o/cochrane/clcentral/articles/731/CN-01329731/frame.html>.

14. Bogdan MA, Aizawa H, Fukuchi Y, Mishima M, Nishimura M, Ichinose M. Efficacy and safety of inhaled formoterol 4.5 and 9 μg twice daily in Japanese and European COPD patients: Phase III study results. BMC Pulmonary Medicine. 2011;11.

15. Bourbeau J, Rouleau MY, Boucher S. Randomised controlled trial of inhaled corticosteroids in patients with chronic obstructive pulmonary disease. Thorax [Internet]. 1998; 53(6):[477-82 pp.]. Available from: <http://onlinelibrary.wiley.com/o/cochrane/clcentral/articles/469/CN-00683469/frame.html>.

16. Boyd G, Morice AH, Pounsford JC, Siebert M, Peslis N, Crawford C. An evaluation of salmeterol in the treatment of chronic obstructive pulmonary disease (COPD). The european respiratory journal [Internet]. 1997; 10(4):[815-21 pp.]. Available from: <http://onlinelibrary.wiley.com/o/cochrane/clcentral/articles/557/CN-00139557/frame.html>.

17. Briggs Jr DD, Covelli H, Lapidus R, Bhattycharya S, Kesten S, Cassino C. Improved daytime spirometric efficacy of tiotropium compared with salmeterol in patients with COPD. Pulmonary Pharmacology and Therapeutics. 2005;18(6):397-404.

18. Brusasco V, Hodder R, Miravitlles M, Korducki L, Towse L, Kesten S. Health outcomes following treatment for six months with once daily tiotropium compared with twice daily salmeterol in patients with COPD. Thorax [Internet]. 2003; 58(5):[399-404 pp.]. Available from: <http://onlinelibrary.wiley.com/o/cochrane/clcentral/articles/036/CN-00431036/frame.html>.

19. Buhl R, Dunn LJ, Disdier C, Lassen C, Amos C, Henley M, et al. Blinded 12-week comparison of once-daily indacaterol and tiotropium in COPD. The European respiratory journal. 2011;38(4):797-803.

20. Buhl R, Gessner C, Schuermann W, Foerster K, Sieder C, Hiltl S, et al. Efficacy and safety of once-daily QVA149 compared with the free combination of once-daily tiotropium plus twice-daily formoterol in patients with moderate-to-severe COPD (QUANTIFY): a randomised, non-inferiority study. Thorax. 2015;70(4):311-9.

21. Buhl R, Maltais F, Abrahams R, Bjermer L, Derom E, Ferguson G, et al. Tiotropium and olodaterol fixed-dose combination versus mono-components in COPD (GOLD 2-4). European Respiratory Journal. 2015;45(4):969-79.

22. Burge PS, Calverley PM, Jones PW, Spencer S, Anderson JA, Maslen TK. Randomised, double blind, placebo controlled study of fluticasone propionate in patients with moderate to severe chronic obstructive pulmonary disease: the ISOLDE trial. BMJ (Clinical research ed). 2000;320(7245):1297-303.

23. Calverley P, Pauwels R, Nieminem M, Stryszak P, Staudinger H, Lee T. Once daily mometasone furoate dry powder inhaler preserves lung function, reduces symptoms and delays exacerbations in patients with COPD previously maintained on ICS. European respiratory journal [Internet]. 2003; 22(Suppl 45):[Abstract No: [155] p.]. Available from: <http://onlinelibrary.wiley.com/o/cochrane/clcentral/articles/398/CN-00483398/frame.html>.

24. Calverley P, Pauwels R, Vestbo J, Jones P, Pride N, Gulsvik A, et al. Combined salmeterol and fluticasone in the treatment of chronic obstructive pulmonary disease: a randomised controlled trial. Lancet. 2003;361(9356):449-56.

25. Calverley PM, Boonsawat W, Cseke Z, Zhong N, Peterson S, Olsson H. Maintenance therapy with budesonide and formoterol in chronic obstructive pulmonary disease. The european respiratory journal [Internet]. 2003; 22(6):[912-9 pp.]. Available from: <http://onlinelibrary.wiley.com/o/cochrane/clcentral/articles/805/CN-00471805/frame.html>.

26. Calverley PM, Kuna P, Monsó E, Costantini M, Petruzzelli S, Sergio F, et al. Beclomethasone/formoterol in the management of COPD: a randomised controlled trial. Respiratory medicine [Internet]. 2010; 104(12):[1858-68 pp.]. Available from: <http://onlinelibrary.wiley.com/o/cochrane/clcentral/articles/083/CN-00781083/frame.html>.

27. Calverley PM, Rennard S, Nelson HS, Karpel JP, Abbate EH, Stryszak P, et al. One-year treatment with mometasone furoate in chronic obstructive pulmonary disease. Respiratory research [Internet]. 2008; 9:[73 p.]. Available from: <http://onlinelibrary.wiley.com/o/cochrane/clcentral/articles/394/CN-00687394/frame.html>.

28. Calverley PMA, Anderson JA, Celli B, Ferguson GT, Jenkins C, Jones PW, et al. Salmeterol and fluticasone propionate and survival in chronic obstructive pulmonary disease. New England Journal of Medicine. 2007;356(8):775-89.

29. Calverley PMA, Anzueto AR, Carter K, Gronke L, Hallmann C, Jenkins C, et al. Tiotropium and olodaterol in the prevention of chronic obstructive pulmonary disease exacerbations (DYNAGITO): a double-blind, randomised, parallel-group, active-controlled trial. The Lancet Respiratory medicine. 2018.

30. Campbell M, Eliraz A, Johansson G, Tornling G, Nihlén U, Bengtsson T, et al. Formoterol for maintenance and as-needed treatment of chronic obstructive pulmonary disease. Respiratory medicine [Internet]. 2005; 99(12):[1511-20 pp.]. Available from: <http://onlinelibrary.wiley.com/o/cochrane/clcentral/articles/397/CN-00552397/frame.html>.

31. Casaburi R, Briggs DD, Donohue JF, Serby CW, Menjoge SS, Witek TJ. The spirometric efficacy of once-daily dosing with tiotropium in stable COPD: a 13-week multicenter trial. The US Tiotropium Study Group. Chest [Internet]. 2000; 118(5):[1294-302 pp.]. Available from: <http://onlinelibrary.wiley.com/o/cochrane/clcentral/articles/619/CN-00328619/frame.html>.

32. Casaburi R, Kukafka D, Cooper CB, Witek TJ, Kesten S. Improvement in exercise tolerance with the combination of tiotropium and pulmonary rehabilitation in patients with COPD. Chest [Internet]. 2005; 127(3):[809-17 pp.]. Available from: <http://onlinelibrary.wiley.com/o/cochrane/clcentral/articles/400/CN-00502400/frame.html>.

33. Casaburi R, Mahler DA, Jones PW, Wanner A, San Pedro G, ZuWallack RL, et al. A long-term evaluation of once-daily inhaled tiotropium in chronic obstructive pulmonary disease. European Respiratory Journal. 2002;19(2):217-24.

34. Cazzola M, Andò F, Santus P, Ruggeri P, Marco F, Sanduzzi A, et al. A pilot study to assess the effects of combining fluticasone propionate/salmeterol and tiotropium on the airflow obstruction of patients with severe-to-very severe COPD. Pulmonary pharmacology & therapeutics [Internet]. 2007; 20(5):[556-61 pp.]. Available from: <http://onlinelibrary.wiley.com/o/cochrane/clcentral/articles/439/CN-00617439/frame.html>.

35. Cazzola M, Lorenzo G, Perna F, Calderaro F, Testi R, Centanni S. Additive effects of salmeterol and fluticasone or theophylline in COPD. Chest [Internet]. 2000; 118(6):[1576-81 pp.]. Available from: <http://onlinelibrary.wiley.com/o/cochrane/clcentral/articles/403/CN-00330403/frame.html>.

36. Celli B, Crater G, Kilbride S, Mehta R, Tabberer M, Kalberg CJ, et al. Once-daily umeclidinium/vilanterol 125/25 μg therapy in COPD. Chest. 2014;145(5):981-91.

37. Celli B, Halpin D, Hepburn R, Byrne N, Keating ET, Goldman M. Symtoms are an important outcome in chronic obstructive pulmonary disease clinical trials: Results of a 3-month comparative study using the Breathlessness, Cough and Sputum Scale (BCSS). Respiratory Medicine. 2003;97(SUPPL. A):S35-S43.

38. Chan CKN, Maltais F, Sigouin C, Haddon JM, Ford GT. A randomized controlled trial to assess the efficacy of tiotropium in Canadian patients with chronic obstructive pulmonary disease. Canadian Respiratory Journal. 2007;14(8):465-72.

39. Chapman KR, Arvidsson P, Chuchalin AG, Dhillon DP, Faurschou P, Goldstein RS, et al. The addition of salmeterol 50 microg bid to anticholinergic treatment in patients with COPD: a randomized, placebo controlled trial. Chronic obstructive pulmonary disease. Canadian respiratory journal [Internet]. 2002; 9(3):[178-85 pp.]. Available from: <http://onlinelibrary.wiley.com/o/cochrane/clcentral/articles/627/CN-00397627/frame.html>.

40. Chapman KR, Rennard SI, Dogra A, Owen R, Lassen C, Kramer B. Long-term safety and efficacy of indacaterol, a long-acting β2-agonist, in subjects with COPD: A randomized, placebo-controlled study. Chest. 2011;140(1):68-75.

41. Choudhury AB, Dawson CM, Kilvington HE, Eldridge S, James WY, Wedzicha JA, et al. Withdrawal of inhaled corticosteroids in people with COPD in primary care: a randomised controlled trial. Respiratory research [Internet]. 2007; 8:[93 p.]. Available from: <http://onlinelibrary.wiley.com/o/cochrane/clcentral/articles/056/CN-00629056/frame.html>.

42. Contoli M, Pauletti A, Rossi MR, Spanevello A, Casolari P, Marcellini A, et al. Long-term effects of inhaled corticosteroids on sputum bacterial and viral loads in COPD. 2017;50(4).

43. Cooper CB, Celli BR, Jardim JR, Wise RA, Legg D, Guo J, et al. Treadmill endurance during 2-year treatment with tiotropium in patients with COPD: a randomized trial. Chest. 2013;144(2):490-7.

44. Covelli H, Bhattacharya S, Cassino C, Conoscenti C, Kesten S. Absence of electrocardiographic findings and improved function with once-daily tiotropium in patients with chronic obstructive pulmonary disease. Pharmacotherapy [Internet]. 2005; 25(12):[1708-18 pp.]. Available from: <http://onlinelibrary.wiley.com/o/cochrane/clcentral/articles/098/CN-00553098/frame.html>.

45. Covelli H, Pek B, Schenkenberger I, Scott-Wilson C, Emmett A, Crim C. Efficacy and safety of fluticasone furoate/vilanterol or tiotropium in subjects with COPD at cardiovascular risk. International journal of chronic obstructive pulmonary disease [Internet]. 2016; 11:[1-12 pp.]. Available from: <http://onlinelibrary.wiley.com/o/cochrane/clcentral/articles/234/CN-01129234/frame.html>.

46. Dahl R, Chapman KR, Rudolf M, Mehta R, Kho P, Alagappan VKT, et al. Safety and efficacy of dual bronchodilation with QVA149 in COPD patients: The ENLIGHTEN study. Respiratory medicine [Internet]. 2013; 107(10):[1558-67 pp.]. Available from: <http://onlinelibrary.wiley.com/o/cochrane/clcentral/articles/888/CN-00915888/frame.html>.

47. Dahl R, Chung KF, Buhl R, Magnussen H, Nonikov V, Jack D, et al. Efficacy of a new once-daily long-acting inhaled β<inf>2</inf>-agonist indacaterol versus twice-daily formoterol in COPD. Thorax. 2010;65(6):473-9.

48. Dahl R, Greefhorst LA, Nowak D, Nonikov V, Byrne AM, Thomson MH, et al. Inhaled formoterol dry powder versus ipratropium bromide in chronic obstructive pulmonary disease. American journal of respiratory and critical care medicine [Internet]. 2001; 164(5):[778-84 pp.]. Available from: <http://onlinelibrary.wiley.com/o/cochrane/clcentral/articles/304/CN-00388304/frame.html>.

49. Decramer M, Anzueto A, Kerwin E, Kaelin T, Richard N, Crater G, et al. Efficacy and safety of umeclidinium plus vilanterol versus tiotropium, vilanterol, or umeclidinium monotherapies over 24 weeks in patients with chronic obstructive pulmonary disease: results from two multicentre, blinded, randomised controlled trials. The Lancet Respiratory medicine. 2014;2(6):472-86.

50. Decramer ML, Chapman KR, Dahl R, Frith P, Devouassoux G, Fritscher C, et al. Once-daily indacaterol versus tiotropium for patients with severe chronic obstructive pulmonary disease (INVIGORATE): a randomised, blinded, parallel-group study. The Lancet Respiratory medicine [Internet]. 2013; 1(7):[524-33 pp.]. Available from: <http://onlinelibrary.wiley.com/o/cochrane/clcentral/articles/863/CN-01118863/frame.html>.

51. Doherty DE, Tashkin DP, Kerwin E, Knorr BA, Shekar T, Banerjee S, et al. Effects of mometasone furoate/formoterol fumarate fixed-dose combination formulation on chronic obstructive pulmonary disease (COPD): results from a 52-week Phase III trial in subjects with moderate-to-very severe COPD. International journal of chronic obstructive pulmonary disease [Internet]. 2012; 7:[57-71 pp.]. Available from: <http://onlinelibrary.wiley.com/o/cochrane/clcentral/articles/386/CN-00833386/frame.html>.

52. Donohue JF, Fogarty C, Lötvall J, Mahler DA, Worth H, Yorgancioǧlu A, et al. Once-daily bronchodilators for chronic obstructive pulmonary disease: Indacaterol versus tiotropium. American Journal of Respiratory and Critical Care Medicine. 2010;182(2):155-62.

53. Donohue JF, Maleki-Yazdi M, Kilbride S, Mehta R, Kalberg CJ, Church A. Efficacy and safety of once-daily umeclidinium/vilanterol 62.5/25 MCG in COPD: A randomized, placebo-controlled, 24-week study. American Journal of Respiratory and Critical Care Medicine. 2013;187.

54. Donohue JF, Niewoehner D, Brooks J, O'Dell D, Church A. Safety and tolerability of once-daily umeclidinium/vilanterol 125/25 mcg and umeclidinium 125 mcg in patients with chronic obstructive pulmonary disease: results from a 52-week, randomized, double-blind, placebo-controlled study. Respiratory research [Internet]. 2014; 15:[78 p.]. Available from: <http://onlinelibrary.wiley.com/o/cochrane/clcentral/articles/246/CN-01115246/frame.html>.

55. Donohue JF, Soong W, Wu X, Shrestha P, Lei A. Long-term safety of aclidinium bromide/formoterol fumarate fixed-dose combination: Results of a randomized 1-year trial in patients with COPD. Respir Med. 2016;116:41-8.

56. Donohue JF, Worsley S, Zhu CQ, Hardaker L, Church A. Improvements in lung function with umeclidinium/vilanterol versus fluticasone propionate/salmeterol in patients with moderate-to-severe COPD and infrequent exacerbations. Respiratory medicine [Internet]. 2015; 109(7):[870-81 pp.]. Available from: <http://onlinelibrary.wiley.com/o/cochrane/clcentral/articles/991/CN-01072991/frame.html>.

57. Dransfield MT, Bourbeau J, Jones PW, Hanania NA, Mahler DA, Vestbo J, et al. Once-daily inhaled fluticasone furoate and vilanterol versus vilanterol only for prevention of exacerbations of COPD: Two replicate double-blind, parallel-group, randomised controlled trials. The Lancet Respiratory Medicine. 2013;1(3):210-23.

58. Dransfield MT, Cockcroft JR, Townsend RR, Coxson HO, Sharma SS, Rubin DB, et al. Effect of fluticasone propionate/salmeterol on arterial stiffness in patients with COPD. Respiratory medicine [Internet]. 2011; 105(9):[1322-30 pp.]. Available from: <http://onlinelibrary.wiley.com/o/cochrane/clcentral/articles/153/CN-00799153/frame.html>.

59. D'Urzo A, Ferguson GT, van Noord JA, Hirata K, Martin C, Horton R, et al. Efficacy and safety of once-daily NVA237 in patients with moderate-to-severe COPD: the GLOW1 trial. Respiratory research. 2011;12:156.

60. D'Urzo A, Rennard S, Kerwin E, Donohue JF, Lei A, Molins E, et al. A randomised double-blind, placebo-controlled, long-term extension study of the efficacy, safety and tolerability of fixed-dose combinations of aclidinium/formoterol or monotherapy in the treatment of chronic obstructive pulmonary disease. Respiratory medicine [Internet]. 2017; 125:[39-48 pp.]. Available from: <http://onlinelibrary.wiley.com/o/cochrane/clcentral/articles/620/CN-01338620/frame.html>.

61. D'Urzo AD, Rennard SI, Kerwin EM, Mergel V, Leselbaum AR, Caracta CF. Efficacy and safety of fixed-dose combinations of aclidinium bromide/formoterol fumarate: the 24-week, randomized, placebo-controlled AUGMENT COPD study. Respiratory research. 2014;15:123.

62. Dusser D, Bravo ML, Iacono P. The effect of tiotropium on exacerbations and airflow in patients with COPD. The european respiratory journal [Internet]. 2006; 27(3):[547-55 pp.]. Available from: <http://onlinelibrary.wiley.com/o/cochrane/clcentral/articles/003/CN-00563003/frame.html>.

63. Feldman G, Siler T, Prasad N, Jack D, Piggott S, Owen R, et al. Efficacy and safety of indacaterol 150 μg once-daily in COPD: A double-blind, randomised, 12-week study. BMC Pulmonary Medicine. 2010;10.

64. Ferguson G, Feldman G, Hofbauer P, Hamilton A, Allen L, Korducki L, et al. Lung function efficacy of olodaterol QD delivered via respimat in COPD patients: Results from two 48-week studies. Respirology (Carlton, Vic) [Internet]. 2014; 19:[119 p.]. Available from: <http://onlinelibrary.wiley.com/o/cochrane/clcentral/articles/784/CN-01059784/frame.html>.

65. Ferguson GT, Anzueto A, Fei R, Emmett A, Knobil K, Kalberg C. Effect of fluticasone propionate/salmeterol (250/50 microg) or salmeterol (50 microg) on COPD exacerbations. Respir Med. 2008;102(8):1099-108.

66. Ferguson GT, Tashkin DP, Skarby T, Jorup C, Sandin K, Greenwood M, et al. Effect of budesonide/formoterol pressurized metered-dose inhaler on exacerbations versus formoterol in chronic obstructive pulmonary disease: the 6-month, randomized RISE (Revealing the Impact of Symbicort in reducing Exacerbations in COPD) study. Respiratory medicine [Internet]. 2017; 132:[31-41 pp.]. Available from: <http://cochranelibrary-wiley.com/o/cochrane/clcentral/articles/741/CN-01425741/frame.html>.

67. Ferguson GT, Taylor AF, Thach C, Wang Q, Schubert-Tennigkeit AA, Patalano F, et al. Long-Term Maintenance Bronchodilation With Indacaterol/Glycopyrrolate Versus Indacaterol in Moderate-to-Severe COPD Patients: The FLIGHT 3 Study. Chronic obstructive pulmonary diseases (Miami, Fla). 2016;3(4):716-28.

68. Freeman D, Lee A, Price D. Efficacy and safety of tiotropium in COPD patients in primary care--the SPiRiva Usual CarE (SPRUCE) study. Respiratory research [Internet]. 2007; 8:[45 p.]. Available from: <http://onlinelibrary.wiley.com/o/cochrane/clcentral/articles/048/CN-00698048/frame.html>.

69. Frith PA, Thompson PJ, Ratnavadivel R, Chang CL, Bremner P, Day P, et al. Glycopyrronium once-daily significantly improves lung function and health status when combined with salmeterol/fluticasone in patients with COPD: the GLISTEN study, a randomised controlled trial. Thorax. 2015;70(6):519-27.

70. Fukuchi Y, Samoro R, Fassakhov R, Taniguchi H, Ekelund J, Carlsson LG. Budesonide/formoterol via Turbuhaler versus formoterol via Turbuhaler in patients with moderate to severe chronic obstructive pulmonary disease: Phase III multinational study results. Respirology (Carlton, Vic) [Internet]. 2013; 18(5):[866-73 pp.]. Available from: <http://onlinelibrary.wiley.com/o/cochrane/clcentral/articles/199/CN-00866199/frame.html>.

71. Gross NJ, Nelson HS, Lapidus RJ, Dunn L, Lynn L, Rinehart M, et al. Efficacy and safety of formoterol fumarate delivered by nebulization to COPD patients. Respir Med. 2008;102(2):189-97.

72. Guido Boom DEN, Rutten-Van Molken M, Tirimanna J, Weel C, Schayck CP. The cost effectiveness of early treatment with fluticasone propionate 250 mug twice a day in subjects with obstructive airway disease: results of the DIMCA program. American journal of respiratory and critical care medicine [Internet]. 2001; 164(11):[2057-66 pp.]. Available from: <http://onlinelibrary.wiley.com/o/cochrane/clcentral/articles/437/CN-00425437/frame.html>.

73. Hanania NA, Crater GD, Morris AN, Emmett AH, O'Dell DM, Niewoehner DE. Benefits of adding fluticasone propionate/salmeterol to tiotropium in moderate to severe COPD. Respir Med. 2012;106(1):91-101.

74. Hanania NA, Darken P, Horstman D, Reisner C, Lee B, Davis S, et al. The efficacy and safety of fluticasone propionate (250 microg)/salmeterol (50 microg) combined in the Diskus inhaler for the treatment of COPD. Chest [Internet]. 2003; 124(3):[834-43 pp.]. Available from: <http://onlinelibrary.wiley.com/o/cochrane/clcentral/articles/377/CN-00440377/frame.html>.

75. Hanania NA, Tashkin DP, Kerwin EM, Donohue JF, Denenberg M, O'Donnell DE, et al. Long-term safety and efficacy of glycopyrrolate/formoterol metered dose inhaler using novel Co-Suspension Delivery Technology in patients with chronic obstructive pulmonary disease. Respir Med. 2017;126:105-15.

76. Hanrahan JP, Hanania NA, Calhoun WJ, Sahn SA, Sciarappa K, Baumgartner RA. Effect of nebulized arformoterol on airway function in COPD: results from two randomized trials. Copd. 2008;5(1):25-34.

77. Hattotuwa KL, Gizycki MJ, Ansari TW, Jeffery PK, Barnes NC. The effects of inhaled fluticasone on airway inflammation in chronic obstructive pulmonary disease: a double-blind, placebo-controlled biopsy study. Am J Respir Crit Care Med. 2002;165(12):1592-6.

78. Ichinose M, Kato M, Takizawa A, Sakamoto W, Gronke L, Tetzlaff K, et al. Long-term safety and efficacy of combined tiotropium and olodaterol in Japanese patients with chronic obstructive pulmonary disease. Respiratory investigation. 2017;55(2):121-9.

79. Ichinose M, Nakamura H, Shijubo N, Saito T, Taniguchi H, Tsuda T, et al. Tolerability and efficacy of budesonide/formoterol via Turbuhaler® vs standard treatment in Japanese patients with moderate to severe COPD: 52-week phase III study results. European Respiratory Journal. 2012;40.

80. Johansson G, Lindberg A, Romberg K, Nordstrom L, Gerken F, Roquet A. Bronchodilator efficacy of tiotropium in patients with mild to moderate COPD. Primary care respiratory journal : journal of the General Practice Airways Group. 2008;17(3):169-75.

81. Jones PW, Rennard SI, Agusti A, Chanez P, Magnussen H, Fabbri L, et al. Efficacy and safety of once-daily aclidinium in chronic obstructive pulmonary disease. Respiratory research. 2011;12:55.

82. Jones PW, Singh D, Bateman ED, Agusti A, Lamarca R, de Miquel G, et al. Efficacy and safety of twice-daily aclidinium bromide in COPD patients: the ATTAIN study. The European respiratory journal. 2012;40(4):830-6.

83. Jung KS, Park HY, Park SY, Kim SK, Kim YK, Shim JJ, et al. Comparison of tiotropium plus fluticasone propionate/salmeterol with tiotropium in COPD: a randomized controlled study. Respir Med. 2012;106(3):382-9.

84. Kardos P, Wencker M, Glaab T, Vogelmeier C. Impact of salmeterol/fluticasone propionate versus salmeterol on exacerbations in severe chronic obstructive pulmonary disease. Am J Respir Crit Care Med. 2007;175(2):144-9.

85. Kerwin E, Hebert J, Gallagher N, Martin C, Overend T, Alagappan VK, et al. Efficacy and safety of NVA237 versus placebo and tiotropium in patients with COPD: the GLOW2 study. The European respiratory journal. 2012;40(5):1106-14.

86. Kerwin EM, D'Urzo AD, Gelb AF, Lakkis H, Garcia Gil E, Caracta CF. Efficacy and safety of a 12-week treatment with twice-daily aclidinium bromide in COPD patients (ACCORD COPD I). Copd. 2012;9(2):90-101.

87. Kerwin EM, Gotfried MH, Lawrence D, Lassen C, Kramer B. Efficacy and tolerability of indacaterol 75 mug once daily in patients aged >/=40 years with chronic obstructive pulmonary disease: results from 2 double-blind, placebo-controlled 12-week studies. Clinical therapeutics. 2011;33(12):1974-84.

88. Kerwin EM, Kalberg CJ, Galkin DV, Zhu CQ, Church A, Riley JH, et al. Umeclidinium/vilanterol as step-up therapy from tiotropium in patients with moderate COPD: a randomized, parallel-group, 12-week study. International journal of chronic obstructive pulmonary disease. 2017;12:745-55.

89. Kerwin EM, Scott-Wilson C, Sanford L, Rennard S, Agusti A, Barnes N, et al. A randomised trial of fluticasone furoate/vilanterol (50/25 mug; 100/25 mug) on lung function in COPD. Respir Med. 2013;107(4):560-9.

90. Kerwin EM, Siler TM, Korenblat PE, White AC, Eckert JH, Cha E, et al. Glycopyrronium twice daily improves lung function and health status and is well tolerated in COPD patients with moderate-to-severe airflow limitation: The GEM2 study. American Journal of Respiratory and Critical Care Medicine. 2015;191.

91. Kinoshita M, Lee SH, Hang LW, Ichinose M, Hosoe M, Okino N, et al. Efficacy and safety of indacaterol 150 and 300 microg in chronic obstructive pulmonary disease patients from six Asian areas including Japan: a 12-week, placebo-controlled study. Respirology. 2012;17(2):379-89.

92. Koch A, Pizzichini E, Hamilton A, Hart L, Korducki L, De Salvo MC, et al. Lung function efficacy and symptomatic benefit of olodaterol once daily delivered via Respimat® versus placebo and formoterol twice daily in patients with GOLD 2-4 COPD: Results from two replicate 48-week studies. International Journal of COPD. 2014;9:697-714.

93. Korn S, Kerwin E, Atis S, Amos C, Owen R, Lassen C. Indacaterol once-daily provides superior efficacy to salmeterol twice-daily in COPD: a 12-week study. Respir Med. 2011;105(5):719-26.

94. Kornmann O, Dahl R, Centanni S, Dogra A, Owen R, Lassen C, et al. Once-daily indacaterol versus twice-daily salmeterol for COPD: a placebo-controlled comparison. The European respiratory journal. 2011;37(2):273-9.

95. LaForce C, Feldman G, Spangenthal S, Eckert JH, Henley M, Patalano F, et al. Efficacy and safety of twice-daily glycopyrrolate in patients with stable, symptomatic COPD with moderate-to-severe airflow limitation: the GEM1 study. International journal of chronic obstructive pulmonary disease. 2016;11:1233-43.

96. Laptseva IM, Laptseva EA, Borshchevsky VV, Gurevich G, Kalechits O. Inhaled budesonide in the management of chronic obstructive pulmonary diseases. European respiratory society annual congress 2002 [Internet]. 2002:[abstract P1584 p.]. Available from: <http://onlinelibrary.wiley.com/o/cochrane/clcentral/articles/252/CN-00430252/frame.html>.

97. Larbig M, Fowlertaylor A, Maitra S, Schubert-Tennigkeit A, Banerji D. Efficacy and safety of IND/GLY versus placebo and tiotropium in symptomatic patients with moderate-to-severe COPD: The 52-week radiate study. Respirology. 2015;20:44.

98. Lee SD, Xie CM, Yunus F, Itoh Y, Ling X, Yu WC, et al. Efficacy and tolerability of budesonide/formoterol added to tiotropium compared with tiotropium alone in patients with severe or very severe COPD: A randomized, multicentre study in East Asia. Respirology. 2016;21(1):119-27.

99. Lee SH, Lee J, Yoo KH, Uh ST, Park MJ, Lee SY, et al. Efficacy and safety of aclidinium bromide in patients with COPD: A phase 3 randomized clinical trial in a Korean population. Respirology. 2015;20(8):1222-8.

100. Lipson DA, Barnacle H, Birk R, Brealey N, Locantore N, Lomas DA, et al. FULFIL Trial: Once-Daily Triple Therapy in Patients with Chronic Obstructive Pulmonary Disease. Am J Respir Crit Care Med. 2017.

101. Lipson DA, Barnhart F, Brealey N, Brooks J, Criner GJ, Day NC, et al. Once-Daily Single-Inhaler Triple versus Dual Therapy in Patients with COPD. The New England journal of medicine. 2018.

102. Mahler DA, Donohue JF, Barbee RA, Goldman MD, Gross NJ, Wisniewski ME, et al. Efficacy of salmeterol xinafoate in the treatment of COPD. Chest. 1999;115(4):957-65.

103. Magnussen H, Bugnas B, van Noord J, Schmidt P, Gerken F, Kesten S. Improvements with tiotropium in COPD patients with concomitant asthma. Respir Med. 2008;102(1):50-6.

104. Magnussen H, Disse B, Rodriguez-Roisin R, Kirsten A, Watz H, Tetzlaff K, et al. Withdrawal of inhaled glucocorticoids and exacerbations of COPD. The New England journal of medicine. 2014;371(14):1285-94.

105. Mahler DA, D'Urzo A, Bateman ED, Ozkan SA, White T, Peckitt C, et al. Concurrent use of indacaterol plus tiotropium in patients with COPD provides superior bronchodilation compared with tiotropium alone: a randomised, double-blind comparison. Thorax. 2012;67(9):781-8.

106. Mahler DA, Gifford AH, Satti A, Jessop N, Eckert JH, D'Andrea P, et al. Long-term safety of glycopyrrolate: A randomized study in patients with moderate-to-severe COPD (GEM3). Respir Med. 2016;115:39-45.

107. Mahler DA, Kerwin E, Ayers T, FowlerTaylor A, Maitra S, Thach C, et al. FLIGHT1 and FLIGHT2: Efficacy and Safety of QVA149 (Indacaterol/Glycopyrrolate) versus Its Monocomponents and Placebo in Patients with Chronic Obstructive Pulmonary Disease. Am J Respir Crit Care Med. 2015;192(9):1068-79.

108. Mahler DA, Wire P, Horstman D, Chang CN, Yates J, Fischer T, et al. Effectiveness of fluticasone propionate and salmeterol combination delivered via the Diskus device in the treatment of chronic obstructive pulmonary disease. Am J Respir Crit Care Med. 2002;166(8):1084-91.

109. Maleki-Yazdi MR, Kaelin T, Richard N, Zvarich M, Church A. Efficacy and safety of umeclidinium/vilanterol 62.5/25 mcg and tiotropium 18 mcg in chronic obstructive pulmonary disease: results of a 24-week, randomized, controlled trial. Respir Med. 2014;108(12):1752-60.

110. Maltais F, O'Donnell D, Galdiz Iturri JB, Kirsten AM, Singh D, Hamilton A, et al. Effect of 12 weeks of once-daily tiotropium/olodaterol on exercise endurance during constant work-rate cycling and endurance shuttle walking in chronic obstructive pulmonary disease. Ther Adv Respir Dis. 2018;12:1753465818755091.

111. Martinez FJ, Boscia J, Feldman G, Scott-Wilson C, Kilbride S, Fabbri L, et al. Fluticasone furoate/vilanterol (100/25; 200/25 mug) improves lung function in COPD: a randomised trial. Respir Med. 2013;107(4):550-9.

112. Martinez FJ, Rabe KF, Ferguson GT, Fabbri LM, Rennard S, Feldman GJ, et al. Efficacy and Safety of Glycopyrrolate/Formoterol Metered Dose Inhaler Formulated Using Co-Suspension Delivery Technology in Patients With COPD. Chest. 2017;151(2):340-57.

113. Moita J, Barbara C, Cardoso J, Costa R, Sousa M, Ruiz J, et al. Tiotropium improves FEV1 in patients with COPD irrespective of smoking status. Pulm Pharmacol Ther. 2008;21(1):146-51.

114. Niewoehner DE, Rice K, Cote C, Paulson D, Cooper JA, Jr., Korducki L, et al. Prevention of exacerbations of chronic obstructive pulmonary disease with tiotropium, a once-daily inhaled anticholinergic bronchodilator: a randomized trial. Annals of internal medicine. 2005;143(5):317-26.

115. Ohar JA, Crater GD, Emmett A, Ferro TJ, Morris AN, Raphiou I, et al. Fluticasone propionate/salmeterol 250/50 mug versus salmeterol 50 mug after chronic obstructive pulmonary disease exacerbation. Respiratory research. 2014;15:105.

116. Paggiaro PL, Dahle R, Bakran I, Frith L, Hollingworth K, Efthimiou J. Multicentre randomised placebo-controlled trial of inhaled fluticasone propionate in patients with chronic obstructive pulmonary disease. Lancet. 1998;351(9105):773-80.

117. Papi A, Dokic D, Tzimas W, Meszaros I, Olech-Cudzik A, Koroknai Z, et al. Fluticasone propionate/formoterol for COPD management: a randomized controlled trial. International journal of chronic obstructive pulmonary disease. 2017;12:1961-71.

118. Papi A, Vestbo J, Fabbri L, Corradi M, Prunier H, Cohuet G, et al. Extrafine inhaled triple therapy versus dual bronchodilator therapy in chronic obstructive pulmonary disease (TRIBUTE): a double-blind, parallel group, randomised controlled trial. Lancet. 2018;391(10125):1076-84.

119. Pauwels RA, Löfdahl CG, Laitinen LA, Schouten JP, Postma DS, Pride NB, et al. Long-term treatment with inhaled budesonide in persons with mild chronic obstructive pulmonary disease who continue smoking. New England Journal of Medicine. 1999;340(25):1948-53.

120. Powrie DJ, Wilkinson TMA, Donaldson GC, Jones P, Scrine K, Viel K, et al. Effect of tiotropium on sputum and serum inflammatory markers and exacerbations in COPD. European Respiratory Journal. 2007;30(3):472-8.

121. Reid DW, Wen Y, Johns DP, Williams TJ, Ward C, Walters EH. Bronchodilator reversibility, airway eosinophilia and anti-inflammatory effects of inhaled fluticasone in COPD are not related. Respirology. 2008;13(6):799-809.

122. Rennard S, Bailey W, Tashkin D, Abrahams R, Horstman D, Ho S, et al. Improvements in airflow & dyspnea in COPD patients following BID treatment with fluticasone propionate (FP) 250mcg & 500mcg for 24 weeks via the diskus(r) inhaler. American journal of respiratory and critical care medicine [Internet]. 2001; 163(5 Suppl):[A279 p.]. Available from: <http://onlinelibrary.wiley.com/o/cochrane/clcentral/articles/689/CN-00451689/frame.html>.

123. Rennard SI, Anderson W, ZuWallack R, Broughton J, Bailey W, Friedman M, et al. Use of a long-acting inhaled beta2-adrenergic agonist, salmeterol xinafoate, in patients with chronic obstructive pulmonary disease. Am J Respir Crit Care Med. 2001;163(5):1087-92.

124. Rennard SI, Scanlon PD, Ferguson GT, Rekeda L, Maurer BT, Garcia Gil E, et al. ACCORD COPD II: a randomized clinical trial to evaluate the 12-week efficacy and safety of twice-daily aclidinium bromide in chronic obstructive pulmonary disease patients. Clin Drug Investig. 2013;33(12):893-904.

125. Rennard SI, Tashkin DP, McElhattan J, Goldman M, Ramachandran S, Martin UJ, et al. Efficacy and tolerability of budesonide/formoterol in one hydrofluoroalkane pressurized metered-dose inhaler in patients with chronic obstructive pulmonary disease: results from a 1-year randomized controlled clinical trial. Drugs. 2009;69(5):549-65.

126. Rossi A, Kristufek P, Levine BE, Thomson MH, Till D, Kottakis J, et al. Comparison of the efficacy, tolerability, and safety of formoterol dry powder and oral, slow-release theophylline in the treatment of COPD. Chest [Internet]. 2002; 121(4):[1058-69 pp.]. Available from: <http://onlinelibrary.wiley.com/o/cochrane/clcentral/articles/464/CN-00379464/frame.html>.

127. Rossi A, Van Der Molen T, Del Olmo R, Papi A, Webhe L, Quinn M, et al. No loss in efficacy following switch from salmeterol/fluticasone combination to indacaterol monotherapy in patients with moderate copd: The instead study. Thorax. 2014;69:A55.

128. Saraç P, Say?ner A. Compare the efficacy and safety of long-acting anticholinergic and a combination of inhaled steroids and long-acting beta-2 agonist in moderate chronic obstructive pulmonary disease. Tuberkuloz ve toraks [Internet]. 2016; 64(2):[112-8 pp.]. Available from: <http://onlinelibrary.wiley.com/o/cochrane/clcentral/articles/778/CN-01177778/frame.html>.

129. Schermer T, Chavannes N, Dekhuijzen R, Wouters E, Muris J, Akkermans R, et al. Fluticasone and N-acetylcysteine in primary care patients with COPD or chronic bronchitis. Respir Med. 2009;103(4):542-51.

130. Shaker SB, Dirksen A, Ulrik CS, Hestad M, Stavngaard T, Laursen LC, et al. The effect of inhaled corticosteroids on the development of emphysema in smokers assessed by annual computed tomography. Copd. 2009;6(2):104-11.

131. Sharafkhaneh A, Southard JG, Goldman M, Uryniak T, Martin UJ. Effect of budesonide/formoterol pMDI on COPD exacerbations: a double-blind, randomized study. Respir Med. 2012;106(2):257-68.

132. Siler TM, Donald AC, O’Dell D, Church A, Fahy WA. Arandomized, parallel-group study to evaluate the efficacy of umeclidinium/vilanterol 62.5/25 μg on health-related quality of life in patients with COPD. International Journal of COPD. 2016;11(1):971-9.

133. Siler TM, Kerwin E, Singletary K, Brooks J, Church A. Efficacy and Safety of Umeclidinium Added to Fluticasone Propionate/Salmeterol in Patients with COPD: Results of Two Randomized, Double-Blind Studies. Copd. 2016;13(1):1-10.

134. Siler TM, Kerwin E, Sousa AR, Donald A, Ali R, Church A. Efficacy and safety of umeclidinium added to fluticasone furoate/vilanterol in chronic obstructive pulmonary disease: Results of two randomized studies. Respir Med. 2015;109(9):1155-63.

135. Siler TM, Nagai A, Scott-Wilson CA, Midwinter DA, Crim C. A randomised, phase III trial of once-daily fluticasone furoate/vilanterol 100/25 mug versus once-daily vilanterol 25 mug to evaluate the contribution on lung function of fluticasone furoate in the combination in patients with COPD. Respir Med. 2017;123:8-17.

136. Singh D, Ferguson GT, Bolitschek J, Gronke L, Hallmann C, Bennett N, et al. Tiotropium + olodaterol shows clinically meaningful improvements in quality of life. Respir Med. 2015;109(10):1312-9.

137. Singh D, Jones PW, Bateman ED, Korn S, Serra C, Molins E, et al. Efficacy and safety of aclidinium bromide/formoterol fumarate fixed-dose combinations compared with individual components and placebo in patients with COPD (ACLIFORM-COPD): a multicentre, randomised study. BMC Pulm Med. 2014;14:178.

138. Singh D, Papi A, Corradi M, Pavli?ová I, Montagna I, Francisco C, et al. Single inhaler triple therapy versus inhaled corticosteroid plus long-acting ?2-agonist therapy for chronic obstructive pulmonary disease (TRILOGY): a double-blind, parallel group, randomised controlled trial. Lancet (London, England) [Internet]. 2016; 388(10048):[963-73 pp.]. Available from: <http://onlinelibrary.wiley.com/o/cochrane/clcentral/articles/145/CN-01180145/frame.html>.

139. Singh D, Worsley S, Zhu CQ, Hardaker L, Church A. Umeclidinium/vilanterol versus fluticasone propionate/salmeterol in COPD: a randomised trial. BMC Pulm Med. 2015;15:91.

140. Stockley RA, Chopra N, Rice L. Addition of salmeterol to existing treatment in patients with COPD: a 12 month study. Thorax. 2006;61(2):122-8.

141. Szafranski W, Cukier A, Ramirez A, Menga G, Sansores R, Nahabedian S, et al. Efficacy and safety of budesonide/formoterol in the management of chronic obstructive pulmonary disease. The European respiratory journal. 2003;21(1):74-81.

142. Tashkin DP, Celli B, Senn S, Burkhart D, Kesten S, Menjoge S, et al. A 4-year trial of tiotropium in chronic obstructive pulmonary disease. The New England journal of medicine. 2008;359(15):1543-54.

143. Tashkin DP, Doherty DE, Kerwin E, Matiz-Bueno CE, Knorr B, Shekar T, et al. Efficacy and safety of a fixed-dose combination of mometasone furoate and formoterol fumarate in subjects with moderate to very severe COPD: results from a 52-week Phase III trial. International journal of chronic obstructive pulmonary disease [Internet]. 2012; 7:[43-55 pp.]. Available from: <http://onlinelibrary.wiley.com/o/cochrane/clcentral/articles/387/CN-00833387/frame.html>.

144. Tashkin DP, Pearle J, Iezzoni D, Varghese ST. Formoterol and tiotropium compared with tiotropium alone for treatment of COPD. Copd [Internet]. 2009; 6(1):[17-25 pp.]. Available from: <http://onlinelibrary.wiley.com/o/cochrane/clcentral/articles/679/CN-00688679/frame.html>.

145. Tashkin DP, Rennard SI, Martin P, Ramachandran S, Martin UJ, Silkoff PE, et al. Efficacy and safety of budesonide and formoterol in one pressurized metered-dose inhaler in patients with moderate to very severe chronic obstructive pulmonary disease: results of a 6-month randomized clinical trial. Drugs. 2008;68(14):1975-2000.

146. Trivedi R, Richard N, Mehta R, Church A. Umeclidinium in patients with COPD: a randomised, placebo-controlled study. The European respiratory journal. 2014;43(1):72-81.

147. Troosters T, Lavoie K, Leidy N, Maltais F, Sedeno M, Janssens W, et al. Effects of bronchodilator therapy and exercise training, added to a self-management behaviour-modification programme, on physical activity in COPD. European Respiratory Journal. 2016;48.

148. Troosters T, Sciurba FC, Decramer M, Siafakas NM, Klioze SS, Sutradhar SC, et al. Tiotropium in patients with moderate COPD naive to maintenance therapy: a randomised placebo-controlled trial. NPJ primary care respiratory medicine. 2014;24:14003.

149. van der Valk P, Monninkhof E, van der Palen J, Zielhuis G, van Herwaarden C. Effect of discontinuation of inhaled corticosteroids in patients with chronic obstructive pulmonary disease: the COPE study. Am J Respir Crit Care Med. 2002;166(10):1358-63.

150. van Grunsven P, Schermer T, Akkermans R, Albers M, van den Boom G, van Schayck O, et al. Short- and long-term efficacy of fluticasone propionate in subjects with early signs and symptoms of chronic obstructive pulmonary disease. Results of the DIMCA study. Respir Med. 2003;97(12):1303-12.

151. van Noord JA, de Munck DR, Bantje TA, Hop WC, Akveld ML, Bommer AM. Long-term treatment of chronic obstructive pulmonary disease with salmeterol and the additive effect of ipratropium. The European respiratory journal. 2000;15(5):878-85.

152. Verkindre C, Bart F, Aguilaniu B, Fortin F, Guerin JC, Le Merre C, et al. The effect of tiotropium on hyperinflation and exercise capacity in chronic obstructive pulmonary disease. Respiration; international review of thoracic diseases. 2006;73(4):420-7.

153. Vestbo J, Anderson JA, Brook RD, Calverley PM, Celli BR, Crim C, et al. Fluticasone furoate and vilanterol and survival in chronic obstructive pulmonary disease with heightened cardiovascular risk (SUMMIT): a double-blind randomised controlled trial. Lancet. 2016;387(10030):1817-26.

154. Vestbo J, Papi A, Corradi M, Blazhko V, Montagna I, Francisco C, et al. Single inhaler extrafine triple therapy versus long-acting muscarinic antagonist therapy for chronic obstructive pulmonary disease (TRINITY): a double-blind, parallel group, randomised controlled trial. Lancet. 2017;389(10082):1919-29.

155. Vestbo J, Sørensen T, Lange P, Brix A, Tone P, Viskum K. Long-term effect of inhaled budesonide in mild and moderate chronic obstructive pulmonary disease: A randomised controlled trial. Lancet. 1999;353(9167):1819-23.

156. Vincken W, Aumann J, Chen H, Henley M, McBryan D, Goyal P. Efficacy and safety of coadministration of once-daily indacaterol and glycopyrronium versus indacaterol alone in COPD patients: the GLOW6 study. International journal of chronic obstructive pulmonary disease. 2014;9:215-28.

157. Vogelmeier C, Hederer B, Glaab T, Schmidt H, Rutten-van Mölken MPMH, Beeh KM, et al. Tiotropium versus salmeterol for the prevention of exacerbations of COPD. New England Journal of Medicine. 2011;364(12):1093-103.

158. Vogelmeier C, Kardos P, Harari S, Gans SJ, Stenglein S, Thirlwell J. Formoterol mono- and combination therapy with tiotropium in patients with COPD: a 6-month study. Respir Med. 2008;102(11):1511-20.

159. Vogelmeier C, Paggiaro PL, Dorca J, Sliwinski P, Mallet M, Kirsten AM, et al. Efficacy and safety of aclidinium/formoterol versus salmeterol/fluticasone: a phase 3 COPD study. The European respiratory journal. 2016;48(4):1030-9.

160. Vogelmeier CF, Bateman ED, Pallante J, Alagappan VK, D'Andrea P, Chen H, et al. Efficacy and safety of once-daily QVA149 compared with twice-daily salmeterol-fluticasone in patients with chronic obstructive pulmonary disease (ILLUMINATE): a randomised, double-blind, parallel group study. The Lancet Respiratory medicine. 2013;1(1):51-60.

161. Voshaar T, Lapidus R, Maleki-Yazdi R, Timmer W, Rubin E, Lowe L, et al. A randomized study of tiotropium Respimat Soft Mist inhaler vs. ipratropium pMDI in COPD. Respir Med. 2008;102(1):32-41.

162. Wadbo M, Lofdahl CG, Larsson K, Skoogh BE, Tornling G, Arwestrom E, et al. Effects of formoterol and ipratropium bromide in COPD: a 3-month placebo-controlled study. The European respiratory journal. 2002;20(5):1138-46.

163. Wang C, Sun T, Huang Y, Humphries M, Bai L, Li L, et al. Effcacy and safety of once-daily glycopyrronium in predominantly Chinese patients with moderate-to-severe chronic obstructive pulmonary disease: The GLOW7 study. International Journal of COPD. 2015;10:57-68.

164. Wedzicha JA, Banerji D, Chapman KR, Vestbo J, Roche N, Ayers RT, et al. Indacaterol-Glycopyrronium versus Salmeterol-Fluticasone for COPD. The New England journal of medicine. 2016;374(23):2222-34.

165. Wedzicha JA, Calverley PMA, Seemungal TA, Hagan G, Ansari Z, Stockley RA. The prevention of chronic obstructive pulmonary disease exacerbations by salmeterol/fluticasone propionate or tiotropium bromide. American Journal of Respiratory and Critical Care Medicine. 2008;177(1):19-26.

166. Wedzicha JA, Decramer M, Ficker JH, Niewoehner DE, Sandstrom T, Taylor AF, et al. Analysis of chronic obstructive pulmonary disease exacerbations with the dual bronchodilator QVA149 compared with glycopyrronium and tiotropium (SPARK): a randomised, double-blind, parallel-group study. The Lancet Respiratory medicine. 2013;1(3):199-209.

167. Wedzicha JA, Singh D, Vestbo J, Paggiaro PL, Jones PW, Bonnet-Gonod F, et al. Extrafine beclomethasone/formoterol in severe COPD patients with history of exacerbations. Respir Med. 2014;108(8):1153-62.

168. Welte T, Miravitlles M, Hernandez P, Eriksson G, Peterson S, Polanowski T, et al. Efficacy and tolerability of budesonide/formoterol added to tiotropium in patients with chronic obstructive pulmonary disease. Am J Respir Crit Care Med. 2009;180(8):741-50.

169. Wise R, Connett J, Weinmann G, Scanlon P, Skeans M. Effect of inhaled triamcinolone on the decline in pulmonary function in chronic obstructive pulmonary disease. The New England journal of medicine. 2000;343(26):1902-9.

170. Yao W, Wang C, Zhong N, Han X, Wu C, Yan X, et al. Effect of once-daily indacaterol in a predominantly Chinese population with chronic obstructive pulmonary disease: a 26-week Asia-Pacific study. Respirology. 2014;19(2):231-8.

171. Zheng J, de Guia T, Wang-Jairaj J, Newlands AH, Wang C, Crim C, et al. Efficacy and safety of fluticasone furoate/vilanterol (50/25 mcg; 100/25 mcg; 200/25 mcg) in Asian patients with chronic obstructive pulmonary disease: a randomized placebo-controlled trial. Current medical research and opinion. 2015;31(6):1191-200.

172. Zheng J, Zhong N, Newlands A, Church A, Goh AH. Efficacy and safety of once-daily inhaled umeclidinium/vilanterol in Asian patients with COPD: results from a randomized, placebo-controlled study. International journal of chronic obstructive pulmonary disease. 2015;10:1753-67.

173. Zheng JP, Yang L, Wu YM, Chen P, Wen ZG, Huang WJ, et al. The efficacy and safety of combination salmeterol (50 microg)/fluticasone propionate (500 microg) inhalation twice daily via accuhaler in Chinese patients with COPD. Chest. 2007;132(6):1756-63.

174. Zhong N, Wang C, Zhou X, Zhang N, Humphries M, Wang L, et al. LANTERN: a randomized study of QVA149 versus salmeterol/fluticasone combination in patients with COPD. International journal of chronic obstructive pulmonary disease. 2015;10:1015-26.

175. Zhong N, Zheng J, Wen F, Yang L, Chen P, Xiu Q, et al. Efficacy and safety of budesonide/formoterol via a dry powder inhaler in Chinese patients with chronic obstructive pulmonary disease. Current medical research and opinion. 2012;28(2):257-65.

176. Zhou Y, Zhong NS, Li X, Chen S, Zheng J, Zhao D, et al. Tiotropium in Early-Stage Chronic Obstructive Pulmonary Disease. The New England journal of medicine. 2017;377(10):923-35.

177. ZuWallack R, Allen L, Hernandez G, Ting N, Abrahams R. Efficacy and safety of combining olodaterol Respimat((R)) and tiotropium HandiHaler((R)) in patients with COPD: results of two randomized, double-blind, active-controlled studies. International journal of chronic obstructive pulmonary disease. 2014;9:1133-44.

178. Sethi S, Kerwin E, Watz H, Ferguson GT, Mroz RM, Segarra R, et al. AMPLIFY: a randomized, Phase III study evaluating the efficacy and safety of aclidinium/formoterol vs monocomponents and tiotropium in patients with moderate-to-very severe symptomatic COPD. International journal of chronic obstructive pulmonary disease. 2019;14:667-82.

179. Chapman KR, Hurst JR, Frent SM, Larbig M, Fogel R, Guerin T, et al. Long-Term Triple Therapy De-escalation to Indacaterol/Glycopyrronium in Patients with Chronic Obstructive Pulmonary Disease (SUNSET): A Randomized, Double-Blind, Triple-Dummy Clinical Trial. Am J Respir Crit Care Med. 2018;198(3):329-39.

180. Ferguson GT, Rabe KF, Martinez FJ, Fabbri LM, Wang C, Ichinose M, et al. Triple therapy with budesonide/glycopyrrolate/formoterol fumarate with co-suspension delivery technology versus dual therapies in chronic obstructive pulmonary disease (KRONOS): a double-blind, parallel-group, multicentre, phase 3 randomised controlled trial. The Lancet Respiratory medicine. 2018;6(10):747-58.

181. Frith PA, Ashmawi S, Krishnamurthy S, Gurgun A, Hristoskova S, Pilipovic V, et al. Efficacy and safety of the direct switch to indacaterol/glycopyrronium from salmeterol/fluticasone in non-frequently exacerbating COPD patients: The FLASH randomized controlled trial. Respirology. 2018;23(12):1152-9.

182. Lipworth BJ, Collier DJ, Gon Y, Zhong N, Nishi K, Chen R, et al. Improved lung function and patient-reported outcomes with co-suspension delivery technology glycopyrrolate/formoterol fumarate metered dose inhaler in COPD: a randomized Phase III study conducted in Asia, Europe, and the USA. International journal of chronic obstructive pulmonary disease. 2018;13:2969-84.

183. Zhao D, Ling C, Guo Q, Jin J, Xu H. Efficacy and safety of tiotropium bromide combined with budesonide/formoterol in the treatment of moderate to severe chronic obstructive pulmonary disease. Experimental and therapeutic medicine. 2018;16(6):4578-84.

184. Ferguson GT, Papi A, Anzueto A, Kerwin EM, Cappelletti C, Duncan EA, et al. Budesonide/formoterol MDI with co-suspension delivery technology in COPD: the TELOS study. The European respiratory journal. 2018;52(3).

185. Wise RA, Chapman KR, Scirica BM, Bhatt DL, Daoud SZ, Zetterstrand S, et al. Effect of Aclidinium Bromide on Major Cardiovascular Events and Exacerbations in High-Risk Patients With Chronic Obstructive Pulmonary Disease: The ASCENT-COPD Randomized Clinical Trial. Jama. 2019;321(17):1693-701.

**Unpublished studies (186-199)**

186. Asai K, Minakata Y, Hirata K, Fukuchi Y, Kitawaki T, Ikeda K, et al. QVA149 once-daily is safe and well tolerated and improves lung function and health status in Japanese patients with COPD: The ARISE study. European Respiratory Journal. 2013;42. (Contact Information: kazuasai@med.osaka-cu.ac.jp)

187. Clerisme-Beaty E, Leidy NK, Mannino DM, Franceschina J, Ting N. Efficacy and safety of tiotropium in patients with chronic obstructive pulmonary disease (COPD) experiencing an acute respiratory tract infection. American Journal of Respiratory and Critical Care Medicine. 2014;189. (Contact Information: gill.sperrin@envisionpharmagroup.com)

188. data U. A multicentre, randomised, double-blind, double dummy, parallel group 12-week exploratory study to compare the effect of the salmeterol/fluticasone propionate combination product (SERETIDE) 50/500mcg bd via the DISKUS/ACCUHALER inhaler with tiotropium bromide 18 mcg od via the Handihaler inhalation device on efficacy and safety in patients with chronic obstructive pulmonary disease (COPD). Glaxosmithkline clinical study register [Internet]. Available from: <http://onlinelibrary.wiley.com/o/cochrane/clcentral/articles/827/CN-00591827/frame.html>. (Contact Information: Study No. SCO40034, https://www.gsk-studyregister.com, e-mail: gsk.korea@gsk.com)

189. data U. A multi-center, randomized, double-blind, parallel-group, comparison of salmeterol xinafoate inhalation Rotadisk versus placebo in subjects with chronic obstructive pulmonary disease treated with current medications. Glaxosmithkline clinical study register [Internet]. Available from: <http://onlinelibrary.wiley.com/o/cochrane/clcentral/articles/563/CN-00591563/frame.html>. (Contact Information: Study No. 408DP-03, https://www.gsk-studyregister.com, e-mail: gsk.korea@gsk.com)

190. data U. A mulit-centre, randomized, double-blind, parallel group study to evaluate the impact on quality of life of adding SEREVENT 50Âµg bid via MDI to patients' existing therapy in patients with chronic obstructive pulmonary disease (COPD). Glaxosmithkline clinical study register [Internet]. Available from: <http://onlinelibrary.wiley.com/o/cochrane/clcentral/articles/474/CN-00790474/frame.html>. (Contact Information: Study No. SMS40298, https://www.gsk-studyregister.com, e-mail: gsk.korea@gsk.com)

191. data U. A Randomized, Double-blind, Double-Dummy, Comparative Clinical Trial of 12-Week Courses of Salmeterol Xinafoate Versus Ipratropium Bromide Versus Placebo (PRN Ventolin) in Subjects With Chronic Obstructive Pulmonary Disease. Glaxosmithkline clinical study register [Internet]. Available from: <http://onlinelibrary.wiley.com/o/cochrane/clcentral/articles/200/CN-00689200/frame.html>. (Contact Information: Study No. SLGA4004, https://www.gsk-studyregister.com, e-mail: gsk.korea@gsk.com)

192. data U. A multicentre, randomised, placebo-controlled, double-blind comparison with 3 parallel groups to investigate the efficacy and safety of inhaled glucocorticoid fluticasone (500 Âµg bd via Diskus) vs. oral glucocorticoid therapy vs. placebo in subjects with chronic obstructive airways disease (COPD) under therapy with salmeterol (50 Âµg bd). Glaxosmithkline clinical study register [Internet]. Available from: <http://onlinelibrary.wiley.com/o/cochrane/clcentral/articles/539/CN-00790539/frame.html>. (Contact Information: Study No. FCO30002, https://www.gsk-studyregister.com, e-mail: gsk.korea@gsk.com)

193. data U. Multicentre, randomised, parallel group, placebo-controlled, double-blind study, stratified on tobacco status at enrolment, evaluating during 6 months the efficacy of salmeterol powder for inhalation, 50 µg two times per day for the reduction of thoracic distension in subjects with chronic obstructive pulmonary disease (COPD). GSK clinical study register [<http://wwwgsk-clinicalstudyregistercom/>] [Internet]. Available from: <http://onlinelibrary.wiley.com/o/cochrane/clcentral/articles/636/CN-00794636/frame.html>. (Contact Information: Study No. SLMF4010, https://www.gsk-studyregister.com, e-mail: gsk.korea@gsk.com)

194. data U. A multicentre, randomised, double-blind, parallel group, 24-week study to compare the effect of the salmeterol/fluticasone propionate combination product 50/250mcg, with salmeterol 50mcg both delivered twice daily via the DISKUS/ACCUHALER inhaler on lung function and dyspnoea in subjects with chronic obstructive pulmonary disease (COPD). GSK clinical study register [<http://wwwgsk-clinicalstudyregistercom/>] [Internet]. Available from: <http://onlinelibrary.wiley.com/o/cochrane/clcentral/articles/743/CN-00591743/frame.html>. (Contact Information: Study No. SCO100470, https://www.gsk-studyregister.com, e-mail: gsk.korea@gsk.com)

195. data U. A multicentre, randomised, double-blind, parallel group, placebo-controlled study to compare the efficacy and safety of inhaled salmeterol/fluticasone propionate combination product 25/250 µg two puffs bd and fluticasone propionate 250µg two puffs bd alone, all administered via metered dose inhalers (MDI), in the treatment of subjects with chronic obstructive pulmonary disease (COPD) for 52 weeks. GSK clinical study register [<http://wwwgsk-clinicalstudyregistercom/>] [Internet]. Available from: <http://onlinelibrary.wiley.com/o/cochrane/clcentral/articles/526/CN-00790526/frame.html>. (Contact Information: Study No. SFCT01 (SCO30002), https://www.gsk-studyregister.com, e-mail: gsk.korea@gsk.com)

196. data U. A multi-centre, randomised, double-blind, parallel group study to investigate the efficacy and safety of the salmeterol/fluticasone propionate combination at a strength of 50/500 µg BD, compared with placebo via Accuhaler, added to usual chronic obstructive pulmonary disease (COPD) therapy, in subjects with COPD for 24 weeks. GSK clinical study register [<http://wwwgsk-clinicalstudyregistercom>] [Internet]. Available from: <http://onlinelibrary.wiley.com/o/cochrane/clcentral/articles/536/CN-00790536/frame.html>. (Contact Information: Study No. SCO100540, https://www.gsk-studyregister.com, e-mail: gsk.korea@gsk.com)

197. data U. A Randomized, Double-Blind, Parallel-Group Clinical Trial Evaluating the Effect of the Fluticasone Propionate/Salmeterol Combination Product 250/50mcg BID Via DISKUS Versus Salmeterol 50mcg BID Via DISKUS on Bone Mineral Density in Subjects With Chronic Obstructive Pulmonary Disease (COPD). <Http://clinicaltrialsgov/show/NCT00355342> [Internet]. Available from: <http://onlinelibrary.wiley.com/o/cochrane/clcentral/articles/365/CN-01108365/frame.html>. (Contact Information: Study No. SCO40041, https://www.gsk-studyregister.com, e-mail: gsk.korea@gsk.com)

198. data U. A 13-week, double-blind, parallel-group, multicentre study to compare the bronchial anti-inflammatory activity of the combination of salmeterol/ fluticasone propionate (SERETIDE™/ADVAIR™/VIANI™) 50/500 mcg twice daily compared with placebo twice daily in patients with Chronic Obstructive Pulmonary Disease. Glaxosmithkline clinical study register [Internet]. Available from: <http://onlinelibrary.wiley.com/o/cochrane/clcentral/articles/140/CN-00790140/frame.html>. (Contact Information: Study No. SCO30005, https://www.gsk-studyregister.com, e-mail: gsk.korea@gsk.com)

199. Garcia Rio F. A randomised, double-blind, placebo-controlled, 12 weeks trial to evaluate the effect of Tiotropium Inhalation Capsules on the magnitude of exercise, measured using an accelerometer, in patients with Chronic Obstructive Pulmonary Disease (COPD). Boehringer Ingelheim Trial Results [Internet]. 2007. Available from: <http://onlinelibrary.wiley.com/o/cochrane/clcentral/articles/396/CN-00833396/frame.html>. (Contact Information: Trial No. 205.269, https://trials.boehringer-ingelheim.com, telephone number: +82-02-709-0112)
