# Supplementary material for: Comparisons of exacerbations and mortality among regular inhaled therapies for patients with stable chronic obstructive pulmonary disease: Systematic review and Bayesian network meta-analysis
Source: PLoS Med. 2019 Nov 15;16(11):e1002958. doi: 10.1371/journal.pmed.1002958 (PMC6857849; doi:10.1371/journal.pmed.1002958)
Supplement: S1 Table — (DOCX) [file pmed.1002958.s005.docx]

**S1 Table. Risk of bias in each domain for the included studies.**

| **Trial (first author, year)** | **Random sequence generation (selection bias)** | **Allocation concealment (selection bias)** | **Blinding of participants and personnel (performance bias)** | **Blinding of outcome assessment (detection bias)** | **Incomplete outcome data (attrition bias)** | **Selective reporting (reporting bias)** | **Other bias** |
| --- | --- | --- | --- | --- | --- | --- | --- |
| Aalber 2002 | Low risk | Low risk | Low risk | Unclear risk | Unclear risk | Unclear risk | Low risk |
| Aaron 2007 | Low risk | Low risk | Low risk | Low risk | Low risk | Low risk | Low risk |
| Abrahams 2013 | Unclear risk | Unclear risk | Unclear risk | Unclear risk | Low risk | Low risk | Low risk |
| Ambrosino 2008 | Unclear risk | Unclear risk | Unclear risk | Unclear risk | Low risk | Low risk | Low risk |
| Anzueto 2009 | Low risk | Unclear risk | Low risk | Unclear risk | High risk | Low risk | Low risk |
| Asai 2015 | Low risk | Low risk | Low risk | Unclear risk | Low risk | Low risk | Low risk |
| Barnes 2010 | Unclear risk | Unclear risk | Low risk | Low risk | Low risk | Unclear risk | Low risk |
| Bateman 2010 (NCT00168831) | Low risk | Low risk | Low risk | Low risk | Low risk | Low risk | Low risk |
| Bateman 2010 (NCT00168844) | Low risk | Low risk | Low risk | Low risk | High risk | Low risk | Low risk |
| Bateman 2010 (NCT00387088) | Low risk | Low risk | Low risk | Unclear risk | Low risk | Low risk | Low risk |
| Bateman 2013 | Low risk | Low risk | High risk | High risk | Low risk | Low risk | Low risk |
| Beeh 2006 | Low risk | Low risk | Low risk | Low risk | Unclear risk | Low risk | Low risk |
| Betsuyaku 2018 | Low risk | Low risk | Low risk | Unclear risk | Low risk | Low risk | Low risk |
| Bhatt 2017 | Low risk | Low risk | Low risk | Low risk | Low risk | Low risk | Unclear risk |
| Bogdan 2011 | Unclear risk | Unclear risk | Low risk | Unclear risk | Low risk | Low risk | Low risk |
| Bourbeau 1998 | Low risk | Low risk | Low risk | Low risk | High risk | High risk | Low risk |
| Boyd 1997 | Unclear risk | Unclear risk | Low risk | Unclear risk | Low risk | Low risk | Low risk |
| Briggs 2005 | Unclear risk | Unclear risk | Unclear risk | Unclear risk | Low risk | Unclear risk | Low risk |
| Brusasco 2003 | Low risk | Low risk | Low risk | Low risk | Low risk | Low risk | Low risk |
| Buhl 2011 | Unclear risk | Unclear risk | Low risk | Low risk | Low risk | Low risk | Low risk |
| Buhl 2015 (NCT01431274, NCT01431287) | Low risk | Unclear risk | Low risk | Low risk | Low risk | Low risk | Low risk |
| Buhl 2015 (NCT01574651) | Low risk | Low risk | Low risk | Low risk | Low risk | Low risk | Low risk |
| Burge 2000 | Low risk | Low risk | Low risk | Unclear risk | High risk | Unclear risk | Low risk |
| Calverley 2003 | Unclear risk | Unclear risk | Low risk | Unclear risk | High risk | Low risk | Unclear risk |
| Calverley 2003 (Study ID: AZSD-039-0670) | Unclear risk | Unclear risk | Low risk | Unclear risk | High risk | Low risk | Low risk |
| Calverley 2003 (Study ID: SFCB3024) | Low risk | Low risk | Low risk | Low risk | Unclear risk | Low risk | Low risk |
| Calverley 2007 | Low risk | Low risk | Low risk | Low risk | Low risk | Low risk | Low risk |
| Calverley 2008 | Low risk | Unclear risk | Unclear risk | Unclear risk | High risk | Unclear risk | Unclear risk |
| Calverley 2010 | Low risk | Low risk | Low risk | Low risk | Low risk | Low risk | Low risk |
| Calverley 2018 | Low risk | Low risk | Low risk | Low risk | Low risk | Low risk | Low risk |
| Campbell 2005 | Low risk | Low risk | Low risk | Low risk | Low risk | Low risk | Low risk |
| Casaburi 2000 | Unclear risk | Unclear risk | Low risk | Unclear risk | Low risk | Unclear risk | High risk |
| Casaburi 2002 | Unclear risk | Unclear risk | Low risk | Unclear risk | Low risk | Unclear risk | High risk |
| Casaburi 2005 | Unclear risk | Unclear risk | Low risk | Unclear risk | Low risk | Unclear risk | Low risk |
| Cazzola 2000 | Low risk | Unclear risk | Low risk | Unclear risk | Unclear risk | Unclear risk | Low risk |
| Cazzola 2007 | Low risk | Unclear risk | Low risk | Unclear risk | Low risk | Unclear risk | Low risk |
| Celli 2003 | Low risk | Low risk | Low risk | Low risk | Low risk | High risk | Unclear risk |
| Celli 2014 | Unclear risk | Unclear risk | Low risk | Unclear risk | High risk | Low risk | Low risk |
| Chan 2007 | Low risk | Unclear risk | Low risk | Unclear risk | Low risk | Low risk | Low risk |
| Chapman 2002 | Low risk | Low risk | Low risk | Unclear risk | Low risk | Unclear risk | Unclear risk |
| Chapman 2011 | Low risk | Low risk | High risk | High risk | Low risk | Low risk | Unclear risk |
| Chapman 2018 | Low risk | Low risk | Low risk | Low risk | Low risk | Low risk | High risk |
| Cheng 2014 | Low risk | Unclear risk | Low risk | Unclear risk | Low risk | Unclear risk | Low risk |
| Choudhury 2007 | Low risk | Low risk | Low risk | Unclear risk | Low risk | Low risk | Low risk |
| Contoli 2017 | Low risk | Unclear risk | Unclear risk | Low risk | Unclear risk | High risk | Low risk |
| Cooper 2013 | Unclear risk | Unclear risk | Low risk | Unclear risk | High risk | Low risk | Low risk |
| Covelli 2005 | Unclear risk | Unclear risk | Unclear risk | Unclear risk | Low risk | Unclear risk | Low risk |
| Covelli 2016 | Low risk | Low risk | Low risk | Low risk | Low risk | Low risk | Unclear risk |
| Dahl 2001 | Unclear risk | Unclear risk | Low risk | Unclear risk | Low risk | Unclear risk | Low risk |
| Dahl 2010 | Low risk | Low risk | Low risk | Low risk | High risk | Low risk | High risk |
| Dahl 2013 | Unclear risk | Unclear risk | Low risk | Low risk | Low risk | Low risk | Low risk |
| Decramer 2013 | Low risk | Low risk | Low risk | Low risk | High risk | Low risk | Low risk |
| Decramer 2014 (NCT01316900) | Low risk | Low risk | Low risk | Low risk | Low risk | Low risk | Low risk |
| Decramer 2014 (NCT01316913) | Low risk | Low risk | Low risk | Low risk | Low risk | Low risk | Low risk |
| Doherty 2012 | Unclear risk | Unclear risk | Low risk | Unclear risk | Low risk | Low risk | Low risk |
| Donohue 2002 | Unclear risk | Unclear risk | Low risk | Unclear risk | High risk | Unclear risk | Low risk |
| Donohue 2010 | Low risk | Low risk | High risk | High risk | Low risk | Low risk | Low risk |
| Donohue 2013 | Low risk | Low risk | Low risk | Unclear risk | Low risk | Low risk | High risk |
| Donohue 2014 | Low risk | Low risk | Low risk | Unclear risk | High risk | Low risk | Low risk |
| Donohue 2015 (NCT01817764) | Low risk | Low risk | Low risk | Unclear risk | Low risk | Low risk | Low risk |
| Donohue 2015 (NCT01879410) | Low risk | Low risk | Low risk | Unclear risk | Low risk | Low risk | High risk |
| Donohue 2016 | Low risk | Unclear risk | Low risk | Low risk | Low risk | Low risk | Low risk |
| Dransfield 2011 | Unclear risk | Unclear risk | Low risk | Unclear risk | Low risk | High risk | Low risk |
| Dransfield 2013 (NCT01009463) | Low risk | Low risk | Low risk | Low risk | Low risk | Low risk | Low risk |
| Dransfield 2013 (NCT01017952) | Low risk | Low risk | Low risk | Low risk | Low risk | Low risk | Low risk |
| D'Urzo 2011 | Unclear risk | Unclear risk | Low risk | Low risk | Low risk | Low risk | Low risk |
| D'Urzo 2014 | Unclear risk | Unclear risk | Low risk | Low risk | Low risk | Low risk | Unclear risk |
| D'Urzo 2017 | Unclear risk | Unclear risk | Low risk | Unclear risk | Low risk | Low risk | Unclear risk |
| Dusser 2006 | Unclear risk | Unclear risk | Low risk | Unclear risk | Low risk | Low risk | Low risk |
| Feldman 2010 | Unclear risk | Unclear risk | Low risk | Low risk | Low risk | Low risk | Low risk |
| Ferguson 2008 | Low risk | Unclear risk | Low risk | Unclear risk | High risk | Low risk | Low risk |
| Ferguson 2014 (NCT00782210) | Unclear risk | Unclear risk | Unclear risk | Unclear risk | Low risk | Low risk | Low risk |
| Ferguson 2014 (NCT00782509) | Unclear risk | Unclear risk | Unclear risk | Unclear risk | Low risk | Low risk | Low risk |
| Ferguson 2016 | Low risk | Unclear risk | Low risk | Unclear risk | Low risk | Low risk | Low risk |
| Ferguson 2017 | Low risk | Low risk | Low risk | Low risk | Low risk | Low risk | High risk |
| Ferguson 2018 (NCT02497001) | Low risk | Low risk | Low risk | Low risk | Low risk | Low risk | Low risk |
| Ferguson 2018 (NCT02766608) | Low risk | Low risk | High risk | Low risk | Low risk | Low risk | Low risk |
| Freeman 2007 | Unclear risk | Unclear risk | Low risk | Unclear risk | Low risk | Low risk | Low risk |
| Frith 2015 | Unclear risk | Unclear risk | Low risk | Unclear risk | Low risk | Low risk | Low risk |
| Frith 2018 | Low risk | Low risk | Low risk | Low risk | Low risk | Low risk | Low risk |
| Fukuchi 2013 | Unclear risk | Unclear risk | Low risk | Unclear risk | Low risk | Low risk | Low risk |
| Gross 2008 | Unclear risk | Unclear risk | Low risk | Unclear risk | Low risk | Unclear risk | Low risk |
| Guido 2001 | Unclear risk | Unclear risk | Unclear risk | Unclear risk | Low risk | High risk | Unclear risk |
| Hanania 2003 | Unclear risk | Unclear risk | Low risk | Low risk | High risk | Low risk | Low risk |
| Hanania 2012 | Unclear risk | Unclear risk | Low risk | Unclear risk | Low risk | Low risk | Low risk |
| Hanania 2017 | Low risk | Unclear risk | Low risk | Low risk | Low risk | Low risk | Low risk |
| Hanrahan 2008 | Unclear risk | Unclear risk | Unclear risk | Unclear risk | High risk | Low risk | Unclear risk |
| Hattotuwa 2002 | Low risk | Unclear risk | Low risk | Low risk | High risk | Unclear risk | Low risk |
| Ichinose 2012 | Unclear risk | Unclear risk | High risk | High risk | Unclear risk | Unclear risk | Low risk |
| Ichinose 2017 | Low risk | Low risk | Low risk | Low risk | Low risk | Low risk | Low risk |
| Johansson 2008 | Unclear risk | Unclear risk | Low risk | Unclear risk | Low risk | Unclear risk | Low risk |
| Jones 2011 (NCT00358436) | Unclear risk | Unclear risk | Low risk | Low risk | High risk | Low risk | Low risk |
| Jones 2011 (NCT00363896) | Unclear risk | Unclear risk | Low risk | Low risk | Low risk | Low risk | Low risk |
| Jung 2012 | Low risk | Unclear risk | Low risk | Low risk | Low risk | Unclear risk | Low risk |
| Kardos 2007 | Low risk | Low risk | Low risk | Unclear risk | Low risk | Unclear risk | Low risk |
| Kerwin 2011 (NCT01068600) | Low risk | Low risk | Low risk | Low risk | Low risk | Low risk | Low risk |
| Kerwin 2011 (NCT01072448) | Low risk | Low risk | Low risk | Low risk | Low risk | Low risk | Low risk |
| Kerwin 2012 (NCT00891462) | Unclear risk | Unclear risk | Low risk | Low risk | Low risk | Low risk | Low risk |
| Kerwin 2012 (NCT00929110) | Unclear risk | Unclear risk | High risk | High risk | Low risk | Low risk | Low risk |
| Kerwin 2013 | Low risk | Low risk | Low risk | Unclear risk | Low risk | Low risk | Low risk |
| Kerwin 2016 | Low risk | Low risk | Low risk | Low risk | Low risk | Low risk | Low risk |
| Kerwin 2017 | Low risk | Low risk | Low risk | Low risk | Low risk | Low risk | Low risk |
| Kinoshita 2012 | Low risk | Unclear risk | Low risk | Unclear risk | Low risk | Low risk | Low risk |
| Koch 2014 (NCT00793624) | Unclear risk | Unclear risk | Unclear risk | Unclear risk | Unclear risk | Low risk | Low risk |
| Koch 2014 (NCT00796653) | Unclear risk | Unclear risk | Unclear risk | Unclear risk | Unclear risk | Low risk | Unclear risk |
| Kornmann 2011 | Low risk | Low risk | Low risk | Low risk | Low risk | Low risk | Low risk |
| LaForce 2016 | Low risk | Low risk | Low risk | Low risk | Low risk | Low risk | Low risk |
| Laptseva 2002 | Unclear risk | Unclear risk | Unclear risk | Unclear risk | Unclear risk | High risk | Unclear risk |
| Larbig 2015 | Unclear risk | Unclear risk | Unclear risk | Low risk | Unclear risk | Low risk | Low risk |
| Lee 2015 | Low risk | Low risk | Low risk | Low risk | Unclear risk | Unclear risk | Low risk |
| Lee 2016 | Unclear risk | Unclear risk | High risk | High risk | Low risk | Low risk | Low risk |
| Lipson 2017 | Unclear risk | Unclear risk | Low risk | Unclear risk | High risk | Low risk | Low risk |
| Lipson 2018 | Unclear risk | Unclear risk | Low risk | Low risk | Low risk | Low risk | Low risk |
| Lipworth 2018 | Low risk | Low risk | Unclear risk | Unclear risk | Low risk | Low risk | Low risk |
| Magnussen 2008 | Unclear risk | Unclear risk | Unclear risk | Unclear risk | Unclear risk | Unclear risk | Low risk |
| Magnussen 2014 | Unclear risk | Unclear risk | High risk | Low risk | Low risk | Low risk | High risk |
| Mahler 1999 | Unclear risk | Unclear risk | Low risk | Unclear risk | Unclear risk | Unclear risk | Low risk |
| Mahler 2002 | Low risk | Unclear risk | Low risk | Low risk | High risk | Low risk | Low risk |
| Mahler 2012 (NCT00846586) | Low risk | Low risk | Low risk | Low risk | Low risk | Low risk | Low risk |
| Mahler 2012 (NCT00877383) | Low risk | Low risk | Low risk | Low risk | Low risk | Low risk | Low risk |
| Mahler 2015 | Low risk | Low risk | Low risk | Low risk | Low risk | Low risk | Low risk |
| Mahler 2016 | Low risk | Low risk | Low risk | Unclear risk | Unclear risk | Low risk | High risk |
| Maleki-Yazdi 2014 | Low risk | Low risk | Low risk | Low risk | Low risk | Low risk | Low risk |
| Maltais 2018 | Unclear risk | Unclear risk | Low risk | Unclear risk | Low risk | Low risk | High risk |
| Martinez 2013 | Low risk | Low risk | Low risk | Unclear risk | Low risk | Low risk | Low risk |
| Martinez 2017 (NCT01854645) | Low risk | Unclear risk | High risk | High risk | Low risk | Low risk | Low risk |
| Martinez 2017 (NCT01854658) | Low risk | Unclear risk | Low risk | Low risk | Low risk | Low risk | Low risk |
| Moita 2008 | Unclear risk | Unclear risk | Unclear risk | Unclear risk | Low risk | Unclear risk | Unclear risk |
| Niewoehner 2005 | Low risk | Unclear risk | Low risk | Unclear risk | Low risk | Unclear risk | Low risk |
| Ohar 2014 | Low risk | Low risk | Low risk | Low risk | Unclear risk | Low risk | Low risk |
| Paggiaro 1998 | Low risk | Low risk | Low risk | Unclear risk | High risk | High risk | Low risk |
| Papi 2017 | Unclear risk | Unclear risk | Low risk | Unclear risk | Low risk | Low risk | Low risk |
| Papi 2018 | Low risk | Low risk | Low risk | Low risk | Low risk | Low risk | High risk |
| Paul 2012 | Unclear risk | Unclear risk | Low risk | Unclear risk | Low risk | Low risk | Low risk |
| Pauwels 1999 | Unclear risk | Unclear risk | Low risk | Low risk | Low risk | High risk | Unclear risk |
| Powrie 2007 | Unclear risk | Unclear risk | Low risk | Low risk | Low risk | Low risk | Unclear risk |
| Reid 2008 | Low risk | Unclear risk | Low risk | Low risk | High risk | Unclear risk | Unclear risk |
| Rennard 2001 | Unclear risk | Unclear risk | Low risk | Unclear risk | High risk | Unclear risk | Low risk |
| Rennard 2001 (Study ID: FLTA3025) | Unclear risk | Unclear risk | Unclear risk | Unclear risk | High risk | Low risk | Low risk |
| Rennard 2009 | Unclear risk | Unclear risk | Low risk | Unclear risk | High risk | High risk | Low risk |
| Rennard 2013 | Unclear risk | Unclear risk | Low risk | Unclear risk | Low risk | Low risk | Low risk |
| Rossi 2002 | Unclear risk | Unclear risk | Low risk | Low risk | Low risk | Low risk | Low risk |
| Rossi 2014 | Low risk | Low risk | Low risk | Low risk | Low risk | Low risk | Low risk |
| Sarac 2016 | Low risk | Unclear risk | High risk | Unclear risk | Low risk | Unclear risk | Unclear risk |
| Schermer 2009 | Low risk | Unclear risk | Low risk | Low risk | High risk | High risk | Low risk |
| Sethi 2018 | Unclear risk | Unclear risk | Unclear risk | Low risk | Low risk | Low risk | Low risk |
| Shaker 2009 | Low risk | Unclear risk | Low risk | Unclear risk | High risk | Low risk | Low risk |
| Sharafkhaneh 2012 | Low risk | Low risk | Low risk | Unclear risk | High risk | Low risk | Unclear risk |
| Siler 2015 (NCT01957163) | Unclear risk | Unclear risk | Low risk | Unclear risk | Low risk | Low risk | Low risk |
| Siler 2015 (NCT02119286) | Low risk | Unclear risk | Low risk | Unclear risk | Low risk | Low risk | Low risk |
| Siler 2016 | Low risk | Low risk | Low risk | Unclear risk | Low risk | Low risk | Low risk |
| Siler 2016 (NCT01772134) | Low risk | Unclear risk | Low risk | Unclear risk | Low risk | Low risk | Low risk |
| Siler 2016 (NCT01772147) | Low risk | Unclear risk | Low risk | Unclear risk | Low risk | Low risk | Low risk |
| Siler 2017 | Unclear risk | Low risk | Low risk | Low risk | Low risk | Low risk | Low risk |
| Singh 2014 | Low risk | Low risk | Low risk | Low risk | High risk | Low risk | Unclear risk |
| Singh 2015 (NCT01822899) | Low risk | Low risk | Low risk | Unclear risk | Low risk | Low risk | High risk |
| Singh 2015 (NCT01964352) | Unclear risk | Unclear risk | Low risk | Unclear risk | Low risk | Low risk | Low risk |
| Singh 2015 (NCT02006732) | Unclear risk | Unclear risk | Low risk | Unclear risk | Low risk | Low risk | Low risk |
| Singh 2016 | Low risk | Unclear risk | Low risk | Low risk | Low risk | Unclear risk | Low risk |
| Sousa 2016 | Low risk | Unclear risk | Low risk | Unclear risk | Low risk | Low risk | Low risk |
| Stockley 2006 | Low risk | Low risk | Low risk | Low risk | High risk | Low risk | Low risk |
| Szafranski 2003 | Unclear risk | Unclear risk | Low risk | Unclear risk | High risk | High risk | Low risk |
| Tashkin 2008 (NCT00144339) | Low risk | Low risk | Low risk | Unclear risk | Unclear risk | Low risk | Low risk |
| Tashkin 2008 (NCT00206154) | Low risk | Unclear risk | Low risk | Unclear risk | Low risk | Low risk | Low risk |
| Tashkin 2009 | Low risk | Unclear risk | Low risk | Unclear risk | Low risk | Unclear risk | Low risk |
| Tashkin 2012 | Low risk | Low risk | Low risk | Unclear risk | Unclear risk | Low risk | Low risk |
| Tonnel 2008 | Low risk | Unclear risk | Low risk | Unclear risk | Low risk | Low risk | Low risk |
| Trivedi 2014 | Low risk | Low risk | Low risk | Low risk | High risk | Low risk | Low risk |
| Trooster 2014 | Unclear risk | Unclear risk | Low risk | High risk | Low risk | Low risk | Low risk |
| Trooster 2016 | Unclear risk | Unclear risk | Low risk | Unclear risk | Unclear risk | Low risk | High risk |
| van der Valk 2002 | Low risk | Unclear risk | Low risk | Low risk | Low risk | Unclear risk | Low risk |
| van Grunsven 2003 | Unclear risk | Unclear risk | Unclear risk | Low risk | Low risk | High risk | Low risk |
| Van Noord 2000 | Unclear risk | Unclear risk | Low risk | Unclear risk | Low risk | Unclear risk | Low risk |
| Verkindre 2006 | Unclear risk | Unclear risk | Low risk | Unclear risk | Unclear risk | Unclear risk | Low risk |
| Vestbo 1999 | Low risk | Low risk | Low risk | Unclear risk | High risk | Unclear risk | Low risk |
| Vestbo 2016 | Low risk | Low risk | Low risk | Low risk | Low risk | Low risk | Low risk |
| Vestbo 2017 | Low risk | Unclear risk | Low risk | Low risk | Low risk | Unclear risk | Low risk |
| Vincken 2014 | Low risk | Low risk | Low risk | Low risk | Low risk | Low risk | Unclear risk |
| Vogelmeier 2008 | Unclear risk | Unclear risk | High risk | High risk | Low risk | Unclear risk | High risk |
| Vogelmeier 2011 | Low risk | Low risk | Low risk | Low risk | Low risk | Low risk | Unclear risk |
| Vogelmeier 2013 | Low risk | Low risk | Low risk | Low risk | Low risk | Low risk | Unclear risk |
| Vogelmeier 2016 | Unclear risk | Unclear risk | Low risk | High risk | Low risk | Low risk | Unclear risk |
| Vogelmeier 2016 (Group B) | Unclear risk | Unclear risk | Low risk | Unclear risk | Unclear risk | Low risk | Low risk |
| Vogelmeier 2016 (Group C) | Unclear risk | Unclear risk | Low risk | Unclear risk | Unclear risk | Low risk | Low risk |
| Vogelmeier 2016 (Group D) | Unclear risk | Unclear risk | Low risk | Unclear risk | Unclear risk | Low risk | Low risk |
| Voshaar 2008 | Unclear risk | Unclear risk | Low risk | Unclear risk | Low risk | Low risk | Low risk |
| Wadbo 2002 | Unclear risk | Unclear risk | Low risk | Unclear risk | High risk | Unclear risk | Low risk |
| Wang 2015 | Unclear risk | Unclear risk | Low risk | Low risk | Low risk | Low risk | Low risk |
| Wedzicha 2008 | Low risk | Low risk | Low risk | Unclear risk | Low risk | Low risk | Unclear risk |
| Wedzicha 2013 | Low risk | Low risk | Low risk | Low risk | Unclear risk | Low risk | Low risk |
| Wedzicha 2014 | Unclear risk | Unclear risk | Low risk | Unclear risk | Low risk | Unclear risk | Low risk |
| Wedzicha 2016 | Low risk | Low risk | Low risk | Low risk | Low risk | Low risk | Low risk |
| Welte 2009 | Low risk | Unclear risk | Low risk | Unclear risk | Low risk | Low risk | Low risk |
| Wise 2000 | Unclear risk | Unclear risk | Low risk | Low risk | Unclear risk | Unclear risk | Unclear risk |
| Wise 2019 | Low risk | Low risk | Low risk | Low risk | Low risk | Low risk | Low risk |
| Yao 2014 | Unclear risk | Unclear risk | Unclear risk | Unclear risk | Low risk | Low risk | Low risk |
| Zhao 2018 | Low risk | Unclear risk | Low risk | Low risk | Low risk | Low risk | Low risk |
| Zheng 2007 | Low risk | Unclear risk | Low risk | Unclear risk | Low risk | Low risk | Unclear risk |
| Zheng 2015 (NCT01376245) | Low risk | Low risk | Low risk | Unclear risk | Low risk | Low risk | Low risk |
| Zheng 2015 (NCT01636713) | Low risk | Low risk | Low risk | Low risk | Unclear risk | Low risk | Low risk |
| Zhong 2012 | Low risk | Unclear risk | Low risk | Unclear risk | Low risk | Low risk | Low risk |
| Zhong 2015 | Low risk | Low risk | Low risk | Low risk | Low risk | Low risk | Low risk |
| Zhou 2017 | Low risk | Unclear risk | Low risk | Unclear risk | High risk | Low risk | Low risk |
| ZuWallack 2014 (NCT01694771) | Unclear risk | Unclear risk | Low risk | Unclear risk | Low risk | Low risk | High risk |
| ZuWallack 2014 (NCT01696058) | Unclear risk | Unclear risk | Low risk | Unclear risk | Low risk | Low risk | Low risk |
| Unpublished (Asai 2013) | Unclear risk | Unclear risk | High risk | High risk | Unclear risk | Unclear risk | Low risk |
| Unpublished (Clerisme-Beaty 2014) | Unclear risk | Unclear risk | Unclear risk | Unclear risk | Low risk | Low risk | Unclear risk |
| Unpublished (Garcia 2007) | Unclear risk | Unclear risk | Low risk | Unclear risk | Low risk | Unclear risk | Low risk |
| Unpublished (408DP-03) | Unclear risk | Unclear risk | Low risk | Unclear risk | Low risk | Unclear risk | Unclear risk |
| Unpublished (FCO30002) | Unclear risk | Unclear risk | Low risk | Unclear risk | Low risk | Low risk | Unclear risk |
| Unpublished (SCO100470) | Unclear risk | Unclear risk | Low risk | Unclear risk | Low risk | Low risk | Low risk |
| Unpublished (SCO100540) | Unclear risk | Unclear risk | Low risk | Unclear risk | Low risk | Low risk | Low risk |
| Unpublished (SCO30005) | Unclear risk | Unclear risk | Low risk | Unclear risk | Low risk | Low risk | Low risk |
| Unpublished (SCO40034) | Unclear risk | Unclear risk | Low risk | Unclear risk | Low risk | Low risk | Low risk |
| Unpublished (SCO40041) | Unclear risk | Unclear risk | Low risk | Unclear risk | High risk | Low risk | Low risk |
| Unpublished (SFCT01) | Unclear risk | Unclear risk | Low risk | Unclear risk | High risk | Low risk | Low risk |
| Unpublished (SLGA4004) | Unclear risk | Unclear risk | Unclear risk | Unclear risk | High risk | Unclear risk | High risk |
| Unpublished (SLMF4010) | Low risk | Unclear risk | Low risk | Low risk | High risk | High risk | Unclear risk |
| Unpublished (SMS40298) | Unclear risk | Unclear risk | Unclear risk | Unclear risk | Low risk | Unclear risk | Unclear risk |
