# Supplementary material for: Comparisons of exacerbations and mortality among regular inhaled therapies for patients with stable chronic obstructive pulmonary disease: Systematic review and Bayesian network meta-analysis
Source: PLoS Med. 2019 Nov 15;16(11):e1002958. doi: 10.1371/journal.pmed.1002958 (PMC6857849; doi:10.1371/journal.pmed.1002958)
Supplement: S2 Table — (DOCX) [file pmed.1002958.s006.docx]

**S2 Table. Assessment of publication bias in the included studies**

| Direct comparison | P-value of Egger test | Estimated OR from Trim-and-fill method | Fail-safe N method |
| --- | --- | --- | --- |
| Total exacerbation |  |  |  |
| Placebo vs. LAMA/LABA | 0.525 | - | - |
| Placebo vs. ICS/LABA | 0.113 | - | - |
| Placebo vs. LAMA | 0.093 | - | - |
| Placebo vs. LABA | 0.015 | 1.125 [1.067-1.185] | Fail-safe N: 654 (k=67) |
| Placebo vs. ICS | 0.423 | - | - |
| ICS/LAMA/LABA vs. LAMA/LABA | 0.921 | - | - |
| ICS/LAMA/LABA vs. ICS/LABA | 0.251 | - | - |
| ICS/LAMA/LABA vs. LAMA | 0.249 | - | - |
| LAMA/LABA vs. ICS/LABA | 0.257 | - | - |
| LAMA/LABA vs. LAMA | 0.249 | - | - |
| LAMA/LABA vs. LABA | 0.235 | - | - |
| ICS/LABA vs. LAMA | 0.313 | - | - |
| ICS/LABA vs. LABA | 0.081 | - | - |
| ICS/LABA vs. ICS | 0.937 | - | - |
| LAMA vs. LABA | 0.035 | 0.900 [0.841-0.964] | Fail-safe N: 8 (k=28) |
| LABA vs. ICS | 0.098 | - | - |
| Moderate to severe exacerbation |  |  |  |
| Placebo vs. LAMA/LABA | 0.068 | - | - |
| Placebo vs. ICS/LABA | 0.340 | - | - |
| Placebo vs. LAMA | 0.589 | - | - |
| Placebo vs. LABA | 0.185 | - | - |
| Placebo vs. ICS | 0.738 | - | - |
| ICS/LAMA/LABA vs. LAMA/LABA | 0.676 | - | - |
| ICS/LAMA/LABA vs. ICS/LABA | 0.690 | - | - |
| LAMA/LABA vs. ICS/LABA | 0.121 | - | - |
| LAMA/LABA vs. LAMA | 0.074 | - | - |
| LAMA/LABA vs. LABA | 0.845 | - | - |
| ICS/LABA vs. LAMA | 0.860 | - | - |
| ICS/LABA vs. LABA | 0.174 | - | - |
| ICS/LABA vs. ICS | 0.539 | - | - |
| LAMA vs. LABA | 0.169 | - | - |
| LABA vs. ICS | 0.862 | - | - |
| All-cause mortality |  |  |  |
| Placebo vs. LAMA/LABA | 0.619 | - | - |
| Placebo vs. ICS/LABA | 0.756 | - | - |
| Placebo vs. LAMA | 0.811 | - | - |
| Placebo vs. LABA | 0.659 | - | - |
| Placebo vs. ICS | 0.886 | - | - |
| ICS/LAMA/LABA vs. LAMA/LABA | 0.159 | - | - |
| ICS/LAMA/LABA vs. ICS/LABA | 0.311 | - | - |
| ICS/LAMA/LABA vs. LAMA | 0.724 | - | - |
| LAMA/LABA vs. ICS/LABA | 0.725 | - | - |
| LAMA/LABA vs. LAMA | 0.558 | - | - |
| LAMA/LABA vs. LABA | 0.521 | - | - |
| ICS/LABA vs. LAMA | 0.954 | - | - |
| ICS/LABA vs. LABA | 0.899 | - | - |
| ICS/LABA vs. ICS | 0.574 | - | - |
| LAMA vs. LABA | 0.572 | - | - |
| LABA vs. ICS | 0.125 | - | - |
| Cardiovascular disease-related mortality |  |  |  |
| Placebo vs. LAMA/LABA | 0.251 | - | - |
| Placebo vs. ICS/LABA | 0.380 | - | - |
| Placebo vs. LAMA | 0.545 | - | - |
| Placebo vs. LABA | 0.846 | - | - |
| Placebo vs. ICS | 0.895 | - | - |
| ICS/LAMA/LABA vs. LAMA/LABA | 0.211 | - | - |
| ICS/LAMA/LABA vs. ICS/LABA | 0.737 | - | - |
| LAMA/LABA vs. ICS/LABA | 0.987 | - | - |
| LAMA/LABA vs. LAMA | 0.847 | - | - |
| LAMA/LABA vs. LABA | 0.581 | - | - |
| ICS/LABA vs. LAMA | 0.947 | - | - |
| ICS/LABA vs. LABA | 0.067 | - | - |
| ICS/LABA vs. ICS | 0.574 | - | - |
| LAMA vs. LABA | 0.326 | - | - |
| LABA vs. ICS | 0.947 | - | - |
| Major adverse cardiac events |  |  |  |
| Placebo vs. LAMA/LABA | 0.968 | - | - |
| Placebo vs. LAMA | 0.571 | - | - |
| Placebo vs. LABA | 0.085 | - | - |
| ICS/LAMA/LABA vs. ICS/LABA | 0.806 | - | - |
| LAMA/LABA vs. ICS/LABA | 0.832 | - | - |
| LAMA/LABA vs. LAMA | 0.650 | - | - |
| LAMA/LABA vs. LABA | 0.921 | - | - |
| LAMA vs. LABA | 0.134 | - | - |
| Pneumonia |  |  |  |
| Placebo vs. LAMA/LABA | 0.291 | - | - |
| Placebo vs. ICS/LABA | 0.133 | - | - |
| Placebo vs. LAMA | 0.703 | - | - |
| Placebo vs. LABA | 0.286 | - | - |
| Placebo vs. ICS | 0.151 | - | - |
| ICS/LAMA/LABA vs. LAMA/LABA | 0.278 | - | - |
| ICS/LAMA/LABA vs. ICS/LABA | 0.759 | - | - |
| ICS/LAMA/LABA vs. LAMA | 0.596 | - | - |
| LAMA/LABA vs. ICS/LABA | 0.261 | - | - |
| LAMA/LABA vs. LAMA | 0.536 | - | - |
| LAMA/LABA vs. LABA | 0.228 | - | - |
| ICS/LABA vs. LAMA | 0.923 | - | - |
| ICS/LABA vs. LABA | 0.159 | - | - |
| ICS/LABA vs. ICS | 0.598 | - | - |
| LAMA vs. LABA | 0.142 | - | - |
| LABA vs. ICS | 0.163 | - | - |

ICS: inhaled corticosteroid, LABA: long-acting beta-agonist, LAMA: long-acting muscarinic antagonist, OR: odds ratio
