# Supplementary material for: Comparisons of exacerbations and mortality among regular inhaled therapies for patients with stable chronic obstructive pulmonary disease: Systematic review and Bayesian network meta-analysis
Source: PLoS Med. 2019 Nov 15;16(11):e1002958. doi: 10.1371/journal.pmed.1002958 (PMC6857849; doi:10.1371/journal.pmed.1002958)
Supplement: S3 Table — CrI, credible interval; ICS, inhaled corticosteroid; LABA, long-acting beta-agonist; LAMA, long-acting muscarinic antagonist; MACE, major adverse cardiovascular event; NMA, network meta-analysis; OR, odds ratio. (DOCX) [file pmed.1002958.s007.docx]

**S3 Table. Results of Bayesian network meta-analyses and direct meta-analysis of total exacerbations, moderate to severe exacerbations, all-cause mortality, cardiovascular disease-related mortality, major adverse cardiac events, and pneumonia according to the drug classes**

| Treatment | Comparator | Network meta-analysis estimate OR | | Posterior Probability (OR>1) | Direct meta-analysis estimate OR | | Inconsistency estimate | | |
| --- | --- | --- | --- | --- | --- | --- | --- | --- | --- |
|  |  | **Posterior median** | **95% CrIs** |  | **OR** | **95% CIs** | **Mean** | **Sd** | **P-value** |
| Total exacerbation | | | | | | | | | |
| ICS/LAMA/LABA | Placebo | 0.57 | 0.5-0.64 | <0.001 | - | - | - | - | - |
| LAMA/LABA | Placebo | 0.7 | 0.65-0.76 | <0.001 | 0.71 | 0.62-0.82 | 0.03 | 0.09 | 0.65 |
| ICS/LABA | Placebo | 0.74 | 0.68-0.81 | <0.001 | 0.80 | 0.72-0.90 | 0.09 | 0.08 | 0.87 |
| LAMA | Placebo | 0.77 | 0.73-0.81 | <0.001 | 0.77 | 0.73-0.82 | 0.02 | 0.07 | 0.64 |
| LABA | Placebo | 0.86 | 0.81-0.9 | <0.001 | 0.88 | 0.84-0.92 | -0.10 | 0.06 | 0.05 |
| ICS | Placebo | 0.84 | 0.76-0.91 | <0.001 | 0.87 | 0.78-0.97 | 0.15 | 0.14 | 0.85 |
| LAMA/LABA | ICS/LAMA/LABA | 1.24 | 1.1-1.41 | 1.000 | 1.13 | 1.04-1.22 | -0.17 | 0.12 | 0.08 |
| ICS/LABA | ICS/LAMA/LABA | 1.31 | 1.16-1.47 | 1.000 | 1.42 | 1.17-1.72 | 0.16 | 0.11 | 0.91 |
| LAMA | ICS/LAMA/LABA | 1.35 | 1.2-1.53 | 1.000 | 1.40 | 1.08-1.82 | -0.02 | 0.13 | 0.43 |
| LABA | ICS/LAMA/LABA | 1.51 | 1.34-1.72 | 1.000 | - | - | - | - | - |
| ICS | ICS/LAMA/LABA | 1.48 | 1.28-1.7 | 1.000 | - | - | - | - | - |
| ICS/LABA | LAMA/LABA | 1.06 | 0.96-1.16 | 0.880 | 1.15 | 0.97-1.35 | 0.12 | 0.10 | 0.90 |
| LAMA | LAMA/LABA | 1.09 | 1.02-1.18 | 0.993 | 1.05 | 1.00-1.11 | 0.00 | 0.07 | 0.52 |
| LABA | LAMA/LABA | 1.22 | 1.13-1.32 | 1.000 | 1.15 | 1.05-1.25 | -0.16 | 0.08 | 0.02 |
| ICS | LAMA/LABA | 1.19 | 1.07-1.33 | 0.999 | - | - | - | - | - |
| LAMA | ICS/LABA | 1.03 | 0.95-1.13 | 0.768 | 1.56 | 0.87-2.79 | 0.23 | 0.16 | 0.92 |
| LABA | ICS/LABA | 1.15 | 1.06-1.25 | 1.000 | 1.29 | 1.16-1.44 | 0.19 | 0.07 | 1.00 |
| ICS | ICS/LABA | 1.13 | 1.02-1.25 | 0.989 | 1.14 | 1.06-1.24 | 0.00 | 0.11 | 0.52 |
| LABA | LAMA | 1.12 | 1.05-1.19 | 0.999 | 1.10 | 1.03-1.17 | -0.09 | 0.07 | 0.10 |
| ICS | LAMA | 1.09 | 0.99-1.2 | 0.958 | - | - | - | - | - |
| ICS | LABA | 0.98 | 0.89-1.07 | 0.328 | 0.95 | 0.86-1.05 | 0.01 | 0.10 | 0.53 |
| Moderate to severe exacerbation | | | | | | | | | |
| ICS/LAMA/LABA | Placebo | 0.56 | 0.47-0.68 | <0.001 | - | - | - | - | - |
| LAMA/LABA | Placebo | 0.69 | 0.6-0.79 | <0.001 | 0.69 | 0.37-1.15 | 0.04 | 0.22 | 0.58 |
| ICS/LABA | Placebo | 0.71 | 0.61-0.82 | <0.001 | 0.80 | 0.62-1.02 | 0.18 | 0.15 | 0.88 |
| LAMA | Placebo | 0.73 | 0.66-0.81 | <0.001 | 0.73 | 0.66-0.81 | -0.03 | 0.15 | 0.43 |
| LABA | Placebo | 0.85 | 0.76-0.95 | 0.003 | 0.90 | 0.84-0.97 | -0.10 | 0.13 | 0.20 |
| ICS | Placebo | 0.87 | 0.74-1.02 | 0.039 | 0.92 | 0.78-1.10 | 0.10 | 0.22 | 0.68 |
| LAMA/LABA | ICS/LAMA/LABA | 1.22 | 1.05-1.44 | 0.995 | 1.15 | 1.05-1.25 | -0.15 | 0.21 | 0.23 |
| ICS/LABA | ICS/LAMA/LABA | 1.27 | 1.07-1.48 | 0.996 | 1.37 | 1.08-1.74 | 0.22 | 0.20 | 0.87 |
| LAMA | ICS/LAMA/LABA | 1.3 | 1.1-1.56 | 0.999 | 1.17 | 0.99-1.38 | -0.20 | 0.31 | 0.26 |
| LABA | ICS/LAMA/LABA | 1.51 | 1.27-1.82 | 1.000 | - | - | - | - | - |
| ICS | ICS/LAMA/LABA | 1.54 | 1.25-1.91 | 1.000 | - | - | - | - | - |
| ICS/LABA | LAMA/LABA | 1.04 | 0.9-1.18 | 0.697 | 1.08 | 0.89-1.31 | 0.09 | 0.16 | 0.72 |
| LAMA | LAMA/LABA | 1.06 | 0.95-1.2 | 0.851 | 1.04 | 0.96-1.13 | 0.04 | 0.15 | 0.62 |
| LABA | LAMA/LABA | 1.24 | 1.09-1.41 | 0.999 | 1.16 | 1.05-1.30 | -0.20 | 0.15 | 0.08 |
| ICS | LAMA/LABA | 1.26 | 1.05-1.51 | 0.993 | - | - | - | - | - |
| LAMA | ICS/LABA | 1.03 | 0.9-1.19 | 0.648 | 1.25 | 0.59-2.68 | 0.11 | 0.24 | 0.68 |
| LABA | ICS/LABA | 1.2 | 1.05-1.37 | 0.997 | 1.43 | 1.18-1.74 | 0.25 | 0.13 | 0.97 |
| ICS | ICS/LABA | 1.22 | 1.04-1.44 | 0.990 | 1.24 | 1.07-1.44 | 0.24 | 0.25 | 0.83 |
| LABA | LAMA | 1.17 | 1.05-1.29 | 0.997 | 1.12 | 1.03-1.22 | -0.16 | 0.14 | 0.12 |
| ICS | LAMA | 1.19 | 1-1.4 | 0.978 | - | - | - | - | - |
| ICS | LABA | 1.02 | 0.87-1.19 | 0.701 | 1.00 | 0.91-1.09 | 0.28 | 0.22 | 0.90 |
| All-cause mortality | | | | | | | | | |
| ICS/LAMA/LABA | Placebo | 0.74 | 0.59-0.93 | 0.004 | - | - | - | - | - |
| LAMA/LABA | Placebo | 0.98 | 0.82-1.18 | 0.395 | 1.23 | 0.65-2.31 | 0.11 | 0.25 | 0.68 |
| ICS/LABA | Placebo | 0.86 | 0.76-0.98 | 0.015 | 0.85 | 0.75-0.97 | -0.02 | 0.13 | 0.45 |
| LAMA | Placebo | 0.98 | 0.88-1.12 | 0.400 | 0.95 | 0.85-1.06 | -0.04 | 0.13 | 0.39 |
| LABA | Placebo | 0.94 | 0.83-1.07 | 0.163 | 0.92 | 0.82-1.04 | 0.00 | 0.12 | 0.49 |
| ICS | Placebo | 0.95 | 0.82-1.09 | 0.242 | 0.96 | 0.85-1.08 | 0.47 | 0.40 | 0.87 |
| LAMA/LABA | ICS/LAMA/LABA | 1.32 | 1.05-1.66 | 0.991 | 1.25 | 0.95-1.64 | -0.01 | 0.20 | 0.48 |
| ICS/LABA | ICS/LAMA/LABA | 1.16 | 0.95-1.42 | 0.925 | 1.14 | 0.91-1.41 | -0.02 | 0.18 | 0.45 |
| LAMA | ICS/LAMA/LABA | 1.33 | 1.07-1.67 | 0.995 | 1.41 | 0.88-2.24 | 0.05 | 0.26 | 0.58 |
| LABA | ICS/LAMA/LABA | 1.27 | 1.02-1.58 | 0.983 | - | - | - | - | - |
| ICS | ICS/LAMA/LABA | 1.29 | 1.01-1.63 | 0.978 | - | - | - | - | - |
| ICS/LABA | LAMA/LABA | 0.88 | 0.73-1.05 | 0.079 | 0.86 | 0.66-1.12 | -0.09 | 0.17 | 0.30 |
| LAMA | LAMA/LABA | 1.01 | 0.86-1.19 | 0.545 | 1.05 | 0.87-1.27 | 0.06 | 0.15 | 0.65 |
| LABA | LAMA/LABA | 0.96 | 0.8-1.16 | 0.350 | 1.02 | 0.67-1.56 | 0.01 | 0.21 | 0.51 |
| ICS | LAMA/LABA | 0.97 | 0.79-1.2 | 0.407 | - | - | - | - | - |
| LAMA | ICS/LABA | 1.15 | 0.99-1.34 | 0.967 | 1.93 | 1.10-3.37 | 0.62 | 0.31 | 0.98 |
| LABA | ICS/LABA | 1.1 | 0.97-1.24 | 0.930 | 1.08 | 0.96-1.22 | -0.11 | 0.14 | 0.22 |
| ICS | ICS/LABA | 1.11 | 0.94-1.28 | 0.914 | 1.14 | 1.00-1.30 | -0.04 | 0.20 | 0.42 |
| LABA | LAMA | 0.95 | 0.82-1.1 | 0.257 | 1.07 | 0.85-1.36 | 0.16 | 0.14 | 0.87 |
| ICS | LAMA | 0.97 | 0.8-1.14 | 0.345 | - | - | - | - | - |
| ICS | LABA | 1.01 | 0.87-1.17 | 0.571 | 1.02 | 0.90-1.16 | 0.00 | 0.19 | 0.50 |
| Cardiovascular disease-related mortality | | | | | | | | | |
| ICS/LAMA/LABA | Placebo | 0.68 | 0.36-1.26 | 0.104 | - | - | - | - | - |
| LAMA/LABA | Placebo | 1.05 | 0.66-1.67 | 0.585 | 1.06 | 0.40-2.82 | 0.37 | 0.65 | 0.72 |
| ICS/LABA | Placebo | 0.81 | 0.57-1.07 | 0.064 | 0.85 | 0.69-1.04 | 0.32 | 0.33 | 0.84 |
| LAMA | Placebo | 1.13 | 0.83-1.56 | 0.785 | 1.06 | 0.79-1.43 | -0.23 | 0.36 | 0.26 |
| LABA | Placebo | 0.91 | 0.69-1.38 | 0.274 | 0.84 | 0.68-1.02 | -0.45 | 0.41 | 0.14 |
| ICS | Placebo | 0.85 | 0.64-1.19 | 0.143 | 0.82 | 0.67-1.00 | -0.14 | 99.64 | 0.50 |
| LAMA/LABA | ICS/LAMA/LABA | 1.54 | 0.84-2.91 | 0.925 | 1.39 | 0.79-2.43 | -0.28 | 0.54 | 0.30 |
| ICS/LABA | ICS/LAMA/LABA | 1.19 | 0.67-2.09 | 0.727 | 1.26 | 0.78-2.03 | 0.24 | 0.52 | 0.67 |
| LAMA | ICS/LAMA/LABA | 1.66 | 0.87-3.29 | 0.938 | - | - | - | - | - |
| LABA | ICS/LAMA/LABA | 1.35 | 0.73-2.76 | 0.826 | - | - | - | - | - |
| ICS | ICS/LAMA/LABA | 1.25 | 0.67-2.49 | 0.760 | - | - | - | - | - |
| ICS/LABA | LAMA/LABA | 0.77 | 0.49-1.17 | 0.109 | 0.87 | 0.55-1.38 | 0.27 | 0.44 | 0.73 |
| LAMA | LAMA/LABA | 1.08 | 0.67-1.74 | 0.621 | 0.91 | 0.50-1.67 | -0.39 | 0.43 | 0.17 |
| LABA | LAMA/LABA | 0.87 | 0.54-1.5 | 0.292 | 1.27 | 0.51-3.22 | 0.63 | 0.61 | 0.85 |
| ICS | LAMA/LABA | 0.81 | 0.49-1.38 | 0.204 | - | - | - | - | - |
| LAMA | ICS/LABA | 1.4 | 0.98-2.11 | 0.967 | 2.17 | 1.02-4.64 | 0.68 | 0.49 | 0.92 |
| LABA | ICS/LABA | 1.13 | 0.84-1.8 | 0.780 | 1.02 | 0.83-1.27 | -0.24 | 0.39 | 0.26 |
| ICS | ICS/LABA | 1.05 | 0.78-1.58 | 0.618 | 0.94 | 0.76-1.17 | -0.38 | 0.43 | 0.19 |
| LABA | LAMA | 0.81 | 0.56-1.26 | 0.150 | 1.13 | 0.57-2.25 | 0.56 | 0.42 | 0.92 |
| ICS | LAMA | 0.75 | 0.5-1.16 | 0.085 | - | - | - | - | - |
| ICS | LABA | 0.93 | 0.63-1.29 | 0.314 | 0.96 | 0.77-1.20 | -0.21 | 0.43 | 0.30 |
| Major adverse cardiac events | | | | | | | | | |
| ICS/LAMA/LABA | Placebo | 0.68 | 0.44-1.12 | 0.058 | - | - | - | - | - |
| LAMA/LABA | Placebo | 0.84 | 0.59-1.26 | 0.176 | 1.53 | 0.69-3.36 | 0.27 | 0.34 | 0.41 |
| ICS/LABA | Placebo | 0.68 | 0.41-1.14 | 0.066 | - | - | - | - | - |
| LAMA | Placebo | 0.84 | 0.65-1.14 | 0.120 | 0.80 | 0.63-1.02 | -0.36 | 0.31 | 0.12 |
| LABA | Placebo | 0.67 | 0.44-1.02 | 0.031 | 0.71 | 0.44-1.13 | 0.24 | 0.42 | 0.70 |
| ICS | Placebo | 0.33 | 0.01-2.15 | 0.143 | - | - | - | - | - |
| LAMA/LABA | ICS/LAMA/LABA | 1.23 | 0.86-1.76 | 0.874 | 1.18 | 0.79-1.77 | -0.14 | 0.37 | 0.35 |
| ICS/LABA | ICS/LAMA/LABA | 0.99 | 0.67-1.43 | 0.470 | 0.68 | 0.68-1.55 | 0.13 | 0.41 | 0.63 |
| LAMA | ICS/LAMA/LABA | 1.24 | 0.82-1.81 | 0.856 | 1.28 | 0.73-2.25 | 0.06 | 0.44 | 0.56 |
| LABA | ICS/LAMA/LABA | 0.98 | 0.58-1.61 | 0.463 | - | - | - | - | - |
| ICS | ICS/LAMA/LABA | 0.48 | 0.01-3.01 | 0.249 | - | - | - | - | - |
| ICS/LABA | LAMA/LABA | 0.81 | 0.54-1.19 | 0.142 | 0.90 | 0.54-1.51 | 0.28 | 0.41 | 0.75 |
| LAMA | LAMA/LABA | 1.01 | 0.74-1.34 | 0.528 | 1.07 | 0.81-1.41 | 0.33 | 0.29 | 0.89 |
| LABA | LAMA/LABA | 0.8 | 0.51-1.2 | 0.143 | 0.80 | 0.43-1.49 | -0.09 | 0.41 | 0.41 |
| ICS | LAMA/LABA | 0.4 | 0.01-2.47 | 0.188 | - | - | - | - | - |
| LAMA | ICS/LABA | 1.25 | 0.79-1.96 | 0.835 | - | - | - | - | - |
| LABA | ICS/LABA | 0.99 | 0.58-1.68 | 0.486 | 1.52 | 0.55-4.21 | 0.52 | 0.64 | 0.79 |
| ICS | ICS/LABA | 0.49 | 0.02-2.99 | 0.250 | 0.79 | 0.10-6.21 | -0.61 | 99.80 | 0.50 |
| LABA | LAMA | 0.79 | 0.52-1.19 | 0.124 | 0.87 | 0.47-1.60 | 0.32 | 0.40 | 0.79 |
| ICS | LAMA | 0.39 | 0.01-2.5 | 0.184 | - | - | - | - | - |
| ICS | LABA | 0.5 | 0.02-3.16 | 0.256 | 0.52 | 0.06-4.33 | -1.42 | 100.1 | 0.50 |
| Pneumonia | | | | | | | | | |
| ICS/LAMA/LABA | Placebo | 1.58 | 1.26-2 | 1.000 | - | - |  |  |  |
| LAMA/LABA | Placebo | 1.05 | 0.88-1.27 | 0.714 | 0.93 | 0.62-1.40 | 0.09 | 0.19 | 0.68 |
| ICS/LABA | Placebo | 1.59 | 1.36-1.91 | 1.000 | 1.35 | 0.97-1.86 | -0.29 | 0.13 | 0.02 |
| LAMA | Placebo | 0.98 | 0.85-1.13 | 0.380 | 1.01 | 0.91-1.13 | 0.12 | 0.13 | 0.82 |
| LABA | Placebo | 1.01 | 0.87-1.18 | 0.534 | 0.93 | 0.80-1.07 | 0.06 | 0.12 | 0.70 |
| ICS | Placebo | 1.39 | 1.15-1.7 | 0.999 | 1.45 | 1.07-1.96 | 0.25 | 0.43 | 0.71 |
| LAMA/LABA | ICS/LAMA/LABA | 0.66 | 0.54-0.83 | <0.001 | 0.65 | 0.53-0.80 | 0.11 | 0.20 | 0.70 |
| ICS/LABA | ICS/LAMA/LABA | 1.01 | 0.84-1.24 | 0.527 | 0.93 | 0.81-1.07 | -0.20 | 0.19 | 0.15 |
| LAMA | ICS/LAMA/LABA | 0.62 | 0.5-0.77 | <0.001 | 0.76 | 0.47-1.27 | 0.23 | 0.30 | 0.79 |
| LABA | ICS/LAMA/LABA | 0.64 | 0.51-0.8 | <0.001 | - | - | - | - | - |
| ICS | ICS/LAMA/LABA | 0.88 | 0.68-1.15 | 0.159 | - | - | - | - | - |
| ICS/LABA | LAMA/LABA | 1.52 | 1.29-1.79 | 1.000 | 1.61 | 1.33-1.95 | 0.15 | 0.17 | 0.81 |
| LAMA | LAMA/LABA | 0.93 | 0.79-1.08 | 0.173 | 0.96 | 0.83-1.11 | 0.02 | 0.14 | 0.55 |
| LABA | LAMA/LABA | 0.96 | 0.81-1.13 | 0.304 | 0.85 | 0.65-1.11 | -0.20 | 0.17 | 0.11 |
| ICS | LAMA/LABA | 1.32 | 1.05-1.65 | 0.991 | - | - | - | - | - |
| LAMA | ICS/LABA | 0.61 | 0.52-0.72 | <0.001 | 0.46 | 0.28-0.73 | -0.36 | 0.29 | 0.11 |
| LABA | ICS/LABA | 0.63 | 0.55-0.72 | <0.001 | 0.66 | 0.57-0.76 | -0.01 | 0.14 | 0.47 |
| ICS | ICS/LABA | 0.87 | 0.72-1.05 | 0.072 | 0.91 | 0.79-1.04 | 0.07 | 0.26 | 0.61 |
| LABA | LAMA | 1.03 | 0.89-1.2 | 0.649 | 1.13 | 0.95-1.36 | -0.05 | 0.15 | 0.38 |
| ICS | LAMA | 1.42 | 1.15-1.76 | 0.999 | - | - | - | - | - |
| ICS | LABA | 1.38 | 1.15-1.67 | 0.999 | 1.37 | 1.17-1.59 | 0.02 | 0.26 | 0.53 |

CrI: credible interval, ICS: inhaled corticosteroid, LABA: long-acting beta-agonist, LAMA: long-acting muscarinic antagonist, NMA: network meta-analysis, OR: odds ratio, Sd: standard deviation

^a^ Effects of ICS on MACE compared to other drug classes or placebo were not analyzed because there were no studies involving ICS.
