# Supplementary material for: Comparisons of exacerbations and mortality among regular inhaled therapies for patients with stable chronic obstructive pulmonary disease: Systematic review and Bayesian network meta-analysis
Source: PLoS Med. 2019 Nov 15;16(11):e1002958. doi: 10.1371/journal.pmed.1002958 (PMC6857849; doi:10.1371/journal.pmed.1002958)
Supplement: S4 Table — Median OR and 95% CrI were calculated as a row to column ratio. CAT, chronic obstructive pulmonary disease assessment test; CrI, credible interval; FEV1, forced expiratory volume in 1 second; ICS, inhaled corticosteroid; LABA, long-acting beta-agonist; LAMA, long-acting muscarinic antagonist; mMRC, modified medical research council; OR, odds ratio; SUCRA, surface under the cumulative ranking curve. (DOCX) [file pmed.1002958.s008.docx]

**S4 Table. Sensitivity analyses of the drug classes to evaluate the effectiveness for reducing total exacerbations**

|  | Placebo | ICS/LAMA/LABA | LAMA/LABA | ICS/LABA | LAMA | LABA | ICS |
| --- | --- | --- | --- | --- | --- | --- | --- |
| Post-bronchodilator FEV1 ≤60% (40 studies, 58,010 patients) | | | | | | | |
| SUCRA | 0.041 | 0.99 | 0.754 | 0.598 | 0.498 | 0.148 | 0.471 |
| Rank | 7 | 1 | 2 | 3 | 4 | 6 | 5 |
| Comparison, median OR with 95% CrI |  |  |  |  |  |  |  |
| Placebo | 1 |  |  |  |  |  |  |
| ICS/LAMA/LABA | 0.49 (0.36-0.64) | 1 |  |  |  |  |  |
| LAMA/LABA | 0.64 (0.46-0.87) | 1.3 (0.95-1.86) | 1 |  |  |  |  |
| ICS/LABA | 0.72 (0.57-0.89) | 1.47 (1.12-1.94) | 1.13 (0.81-1.53) | 1 |  |  |  |
| LAMA | 0.75 (0.64-0.88) | 1.54 (1.2-2.05) | 1.17 (0.89-1.62) | 1.05 (0.85-1.32) | 1 |  |  |
| LABA | 0.93 (0.76-1.13) | 1.9 (1.42-2.62) | 1.45 (1.05-2.04) | 1.29 (1.06-1.6) | 1.23 (1-1.5) | 1 |  |
| ICS | 0.77 (0.58-1) | 1.57 (1.11-2.26) | 1.2 (0.82-1.78) | 1.07 (0.82-1.4) | 1.02 (0.76-1.34) | 0.83 (0.64-1.05) | 1 |
| Post-bronchodilator FEV1 ≤50% (23 studies, 27,952 patients) | | | | | | | |
| SUCRA | 0.05 | 0.953 | 0.733 | 0.564 | 0.564 | 0.18 | 0.456 |
| Rank | 7 | 1 | 2 | 3 | 4 | 6 | 5 |
| Comparison, median OR with 95% CrI |  |  |  |  |  |  |  |
| Placebo | 1 |  |  |  |  |  |  |
| ICS/LAMA/LABA | 0.35 (0.17-0.63) | 1 |  |  |  |  |  |
| LAMA/LABA | 0.45 (0.18-1.02) | 1.27 (0.66-2.54) | 1 |  |  |  |  |
| ICS/LABA | 0.55 (0.33-0.89) | 1.59 (1.04-2.56) | 1.24 (0.6-2.69) | 1 |  |  |  |
| LAMA | 0.55 (0.28-0.99) | 1.56 (1.06-2.36) | 1.22 (0.64-2.44) | 0.98 (0.62-1.52) | 1 |  |  |
| LABA | 0.82 (0.5-1.28) | 2.35 (1.44-4.11) | 1.83 (0.86-4.2) | 1.48 (1.06-2.08) | 1.5 (0.94-2.54) | 1 |  |
| ICS | 0.62 (0.33-1.16) | 1.78 (0.91-3.73) | 1.38 (0.57-3.64) | 1.12 (0.65-1.99) | 1.13 (0.58-2.34) | 0.76 (0.43-1.36) | 1 |
| Total exacerbation ≥1 in the past year (38 studies, 69,741 patients) | | | | | | | |
| SUCRA | 0.078 | 0.992 | 0.726 | 0.62 | 0.487 | 0.116 | 0.48 |
| Rank | 7 | 1 | 2 | 3 | 4 | 6 | 5 |
| Comparison, median OR with 95% CrI |  |  |  |  |  |  |  |
| Placebo | 1 |  |  |  |  |  |  |
| ICS/LAMA/LABA | 0.54 (0.4-0.72) | 1 |  |  |  |  |  |
| LAMA/LABA | 0.68 (0.49-0.93) | 1.25 (0.99-1.65) | 1 |  |  |  |  |
| ICS/LABA | 0.72 (0.57-0.93) | 1.35 (1.08-1.68) | 1.07 (0.82-1.38) | 1 |  |  |  |
| LAMA | 0.77 (0.59-1) | 1.43 (1.14-1.85) | 1.14 (0.89-1.49) | 1.07 (0.85-1.36) | 1 |  |  |
| LABA | 0.97 (0.78-1.21) | 1.8 (1.4-2.35) | 1.43 (1.07-1.91) | 1.34 (1.12-1.62) | 1.25 (1-1.58) | 1 |  |
| ICS | 0.78 (0.6-1.03) | 1.46 (1.04-2.04) | 1.16 (0.8-1.65) | 1.08 (0.82-1.43) | 1.01 (0.73-1.4) | 0.81 (0.62-1.05) | 1 |
| Total exacerbation ≥2 or severe exacerbation ≥1 in the past year (4 studies, 4,250 patients) | | | | | | | |
| SUCRA | 0.241 | 0.740 | - | 0.681 | - | 0.253 | 0.588 |
| Rank | 5 | 1 | - | 2 | - | 4 | 3 |
| Comparison, median OR with 95% CrI |  |  |  |  |  |  |  |
| Placebo | 1 |  |  |  |  |  |  |
| ICS/LAMA/LABA | 0.22 (0.00-9.44) | 1 |  |  |  |  |  |
| LAMA/LABA | - | - | - |  |  |  |  |
| ICS/LABA | 0.32 (0.03-2.83) | 1.44 (0.07-29.38) | - | 1 |  |  |  |
| LAMA | - | - | - | - | - |  |  |
| LABA | 0.93 (0.16-7.06) | 4.54 (0.12-170.20) | - | 2.47 (0.16-36.63) | - | 1 |  |
| ICS | 0.40 (0.02-6.52) | 1.83 (0.03-121.10) | - | 0.79 (0.05-12.88) | - | 3.15 (0.43-21.94) | 1 |
| mMRC scale ≥2 or CAT score ≥10 (44 studies, 69,189 patients) | | | | | | | |
| SUCRA | 0.109 | 0.989 | 0.826 | 0.54 | 0.64 | 0.281 | 0.114 |
| Rank | 7 | 1 | 2 | 4 | 3 | 5 | 6 |
| Comparison, median OR with 95% CrI |  |  |  |  |  |  |  |
| Placebo | 1 |  |  |  |  |  |  |
| ICS/LAMA/LABA | 0.68 (0.57-0.78) | 1 |  |  |  |  |  |
| LAMA/LABA | 0.74 (0.64-0.84) | 1.09 (0.97-1.23) | 1 |  |  |  |  |
| ICS/LABA | 0.84 (0.75-0.93) | 1.24 (1.1-1.43) | 1.14 (1.02-1.28) | 1 |  |  |  |
| LAMA | 0.8 (0.7-0.91) | 1.19 (1.04-1.38) | 1.09 (0.96-1.25) | 0.96 (0.84-1.08) | 1 |  |  |
| LABA | 0.95 (0.85-1.05) | 1.4 (1.22-1.64) | 1.28 (1.14-1.47) | 1.13 (1.02-1.25) | 1.18 (1.03-1.35) | 1 |  |
| ICS | 1 (0.88-1.14) | 1.48 (1.26-1.77) | 1.36 (1.17-1.59) | 1.2 (1.05-1.35) | 1.25 (1.06-1.47) | 1.06 (0.93-1.2) | 1 |
| Study duration of ≥24 weeks (133 studies, 185,571 patients) | | | | | | | |
| SUCRA | <0.001 | 0.998 | 0.826 | 0.591 | 0.578 | 0.225 | 0.282 |
| Rank | 7 | 1 | 2 | 3 | 4 | 6 | 5 |
| Comparison, median OR with 95% CrI |  |  |  |  |  |  |  |
| Placebo | 1 |  |  |  |  |  |  |
| ICS/LAMA/LABA | 0.62 (0.54-0.7) | 1 |  |  |  |  |  |
| LAMA/LABA | 0.71 (0.65-0.77) | 1.15 (1.02-1.3) | 1 |  |  |  |  |
| ICS/LABA | 0.77 (0.71-0.84) | 1.26 (1.11-1.42) | 1.09 (0.99-1.2) | 1 |  |  |  |
| LAMA | 0.78 (0.73-0.82) | 1.26 (1.12-1.43) | 1.09 (1.01-1.19) | 1 (0.92-1.1) | 1 |  |  |
| LABA | 0.88 (0.82-0.93) | 1.43 (1.26-1.62) | 1.24 (1.14-1.35) | 1.14 (1.05-1.24) | 1.13 (1.05-1.21) | 1 |  |
| ICS | 0.86 (0.79-0.94) | 1.4 (1.21-1.62) | 1.22 (1.08-1.36) | 1.11 (1.01-1.23) | 1.11 (1-1.23) | 0.98 (0.89-1.08) | 1 |
| Study duration of ≥48 weeks (74 studies, 130,853 patients) | | | | | | | |
| SUCRA | 0.001 | 0.998 | 0.774 | 0.55 | 0.638 | 0.197 | 0.342 |
| Rank | 7 | 1 | 2 | 4 | 3 | 6 | 5 |
| Comparison, median OR with 95% CrI |  |  |  |  |  |  |  |
| Placebo | 1 |  |  |  |  |  |  |
| ICS/LAMA/LABA | 0.65 (0.57-0.75) | 1 |  |  |  |  |  |
| LAMA/LABA | 0.77 (0.69-0.85) | 1.17 (1.04-1.34) | 1 |  |  |  |  |
| ICS/LABA | 0.81 (0.75-0.88) | 1.25 (1.09-1.42) | 1.06 (0.95-1.18) | 1 |  |  |  |
| LAMA | 0.79 (0.74-0.85) | 1.22 (1.07-1.39) | 1.04 (0.95-1.13) | 0.98 (0.9-1.07) | 1 |  |  |
| LABA | 0.91 (0.85-0.97) | 1.39 (1.22-1.59) | 1.19 (1.08-1.3) | 1.12 (1.04-1.2) | 1.14 (1.06-1.23) | 1 |  |
| ICS | 0.87 (0.78-0.95) | 1.33 (1.13-1.55) | 1.13 (0.99-1.28) | 1.07 (0.95-1.18) | 1.09 (0.97-1.22) | 0.96 (0.86-1.05) | 1 |

CrI: credible interval, CAT: chronic obstructive pulmonary disease assessment test, FEV1: forced expiratory volume in 1 second, ICS: inhaled corticosteroid, LABA: long-acting beta-agonist, LAMA: long-acting muscarinic antagonist, mMRC: modified medical research council, OR: odds ratio, SUCRA: surface under the cumulative ranking curve

Median odds ratio and 95% credible interval were calculated as a row to column ratio.
