# Supplementary material for: Comparisons of exacerbations and mortality among regular inhaled therapies for patients with stable chronic obstructive pulmonary disease: Systematic review and Bayesian network meta-analysis
Source: PLoS Med. 2019 Nov 15;16(11):e1002958. doi: 10.1371/journal.pmed.1002958 (PMC6857849; doi:10.1371/journal.pmed.1002958)
Supplement: S5 Table — CrI, credible interval; FEV1, forced expiratory volume in 1 second; mMRC, modified medical research council. (DOCX) [file pmed.1002958.s009.docx]

**S5 Table. Network meta-regression analysis evaluating the relationship between the covariates and total exacerbations**

|  | Regression coefficient (beta), median | 95% CrI | P(beta<0) |
| --- | --- | --- | --- |
| Post-bronchodilator FEV1% of predicted (%) | -0.03 | -0.05, -0.003 | 0.990 |
| Total exacerbation ≥1 in the past year (%) | -0.01 | -0.03, 0.01 | 0.871 |
| Total exacerbation ≥2 or severe exacerbation ≥1 in the past year (%) | -0.01 | -0.05, 0.03 | 0.747 |
| Serum eosinophil (%) | 0.26 | -3.70, 4.17 | 0.454 |
| mMRC scale | -0.29 | -1.96, 1.16 | 0.660 |
| Reversibility (%) | -0.01 | -0.05, 0.02 | 0.782 |

CrI: credible interval, FEV1: forced expiratory volume in 1 second, mMRC: modified medical research council
