# Supplementary material for: Comparisons of exacerbations and mortality among regular inhaled therapies for patients with stable chronic obstructive pulmonary disease: Systematic review and Bayesian network meta-analysis
Source: PLoS Med. 2019 Nov 15;16(11):e1002958. doi: 10.1371/journal.pmed.1002958 (PMC6857849; doi:10.1371/journal.pmed.1002958)
Supplement: S6 Table — Median OR and 95% CrI were calculated as a row to column ratio. CAT, chronic obstructive pulmonary disease assessment test; CrI, credible interval; FEV1, forced expiratory volume in 1 second; ICS, inhaled corticosteroid; LABA, long-acting beta-agonist; LAMA, long-acting muscarinic antagonist; mMRC, modified medical research council; NMA, network meta-analysis; OR, odds ratio; SUCRA, surface under the cumulative ranking curve. (DOCX) [file pmed.1002958.s010.docx]

**S6 Table. Network meta-analysis adjusted by the predicted post-bronchodilator FEV1% to evaluate effectiveness in reducing total exacerbations**

|  | Placebo | ICS/LAMA/LABA | LAMA/LABA | ICS/LABA | LAMA | LABA | ICS |
| --- | --- | --- | --- | --- | --- | --- | --- |
| Post-bronchodilator FEV1% of predicted (%) (161 studies, 192,373 patients) | | | | | | | |
| SUCRA | <0.001 | 1.000 | 0.809 | 0.66 | 0.517 | 0.23 | 0.284 |
| Rank | 7 | 1 | 2 | 3 | 4 | 6 | 5 |
| Comparison, median OR with 95% CrI |  |  |  |  |  |  |  |
| Placebo | 1 |  |  |  |  |  |  |
| ICS/LAMA/LABA | 0.58 (0.51-0.66) | 1 |  |  |  |  |  |
| LAMA/LABA | 0.69 (0.63-0.75) | 1.19 (1.06-1.36) | 1 |  |  |  |  |
| ICS/LABA | 0.73 (0.66-0.8) | 1.26 (1.12-1.42) | 1.06 (0.95-1.16) | 1 |  |  |  |
| LAMA | 0.77 (0.72-0.82) | 1.32 (1.17-1.5) | 1.11 (1.02-1.21) | 1.05 (0.95-1.16) | 1 |  |  |
| LABA | 0.86 (0.8-0.91) | 1.47 (1.3-1.68) | 1.23 (1.13-1.35) | 1.17 (1.07-1.28) | 1.11 (1.03-1.2) | 1 |  |
| ICS | 0.84 (0.75-0.93) | 1.44 (1.24-1.69) | 1.21 (1.07-1.38) | 1.15 (1.02-1.29) | 1.09 (0.97-1.23) | 0.98 (0.88-1.09) | 1 |

CrI: credible interval, CAT: chronic obstructive pulmonary disease assessment test, FEV1: forced expiratory volume in 1 second, ICS: inhaled corticosteroid, LABA: long-acting beta-agonist, LAMA: long-acting muscarinic antagonist, mMRC: modified medical research council, OR: odds ratio, SUCRA: surface under the cumulative ranking curve

Median odds ratio and 95% credible interval were calculated as a row to column ratio.
