# Supplementary material for: Comparisons of exacerbations and mortality among regular inhaled therapies for patients with stable chronic obstructive pulmonary disease: Systematic review and Bayesian network meta-analysis
Source: PLoS Med. 2019 Nov 15;16(11):e1002958. doi: 10.1371/journal.pmed.1002958 (PMC6857849; doi:10.1371/journal.pmed.1002958)
Supplement: S7 Table — Median OR and 95% CrI were calculated as a row to column ratio. CAT, chronic obstructive pulmonary disease assessment test; CrI, credible interval; FEV1, forced expiratory volume in 1 second; ICS, inhaled corticosteroid; LABA, long-acting beta-agonist; LAMA, long-acting muscarinic antagonist; mMRC, modified medical research council; OR, odds ratio; SUCRA, surface under the cumulative ranking curve. (DOCX) [file pmed.1002958.s011.docx]

**S7 Table. Sensitivity analyses of the drug classes to evaluate their effectiveness in reducing moderate to severe exacerbations**

|  | Placebo | ICS/LAMA/LABA | LAMA/LABA | ICS/LABA | LAMA | LABA | ICS |
| --- | --- | --- | --- | --- | --- | --- | --- |
| Post-bronhodilator FEV1 ≤60% (18 studies, 33,704 patients) | | | | | | | |
| SUCRA | 0.192 | 0.86 | 0.68 | 0.528 | 0.624 | 0.178 | 0.438 |
| Rank | 6 | 1 | 2 | 4 | 3 | 7 | 5 |
| Comparison, median OR with 95% CrI |  |  |  |  |  |  |  |
| Placebo | 1 |  |  |  |  |  |  |
| ICS/LAMA/LABA | 0.46 (0.12-1.32) | 1 |  |  |  |  |  |
| LAMA/LABA | 0.55 (0.15-1.52) | 1.19 (0.64-2.34) | 1 |  |  |  |  |
| ICS/LABA | 0.63 (0.18-1.71) | 1.38 (0.74-2.34) | 1.15 (0.59-2.03) | 1 |  |  |  |
| LAMA | 0.59 (0.18-1.44) | 1.26 (0.7-2.44) | 1.05 (0.63-1.91) | 0.92 (0.55-1.71) | 1 |  |  |
| LABA | 0.92 (0.28-2.67) | 2.01 (0.96-4.34) | 1.68 (0.8-3.71) | 1.48 (0.83-2.71) | 1.57 (0.83-3.17) | 1 |  |
| ICS | 0.71 (0.21-2.14) | 1.57 (0.54-4.42) | 1.32 (0.46-3.7) | 1.16 (0.44-2.96) | 1.25 (0.46-3.19) | 0.79 (0.33-1.63) | 1 |
| Post-bronchodilator FEV1 ≤50% (12 studies, 20,654 patients) | | | | | | | |
| SUCRA | - | 0.783 | 0.581 | 0.492 | 0.549 | 0.095 | - |
| Rank | - | 1 | 2 | 4 | 3 | 5 | - |
| Comparison, median OR with 95% CrI |  |  |  |  |  |  |  |
| Placebo | - |  |  |  |  |  |  |
| ICS/LAMA/LABA | - | 1 |  |  |  |  |  |
| LAMA/LABA | - | 1.20 (0.38-4.23) | 1 |  |  |  |  |
| ICS/LABA | - | 1.37 (0.53-3.24) | 1.13 (0.28-4.20) | 1 |  |  |  |
| LAMA | - | 1.26 (0.49-3.43) | 1.05 (0.32-3.57) | 0.93 (0.38-2.50) | 1 |  |  |
| LABA | - | 2.27 (0.78-7.05) | 1.87 (0.45-8.24) | 1.69 (0.81-3.59) | 1.76 (0.66-5.25) | 1 |  |
| ICS | - | - | - | - | - | - | - |
| Total exacerbation ≥1 in the past year (21 studies, 53,886 patients) | | | | | | | |
| SUCRA | 0.086 | 0.873 | 0.628 | 0.524 | 0.535 | 0.166 | 0.687 |
| Rank | 7 | 1 | 3 | 5 | 4 | 6 | 2 |
| Comparison, median OR with 95% CrI |  |  |  |  |  |  |  |
| Placebo | 1 |  |  |  |  |  |  |
| ICS/LAMA/LABA | 0.48 (0.23-0.91) | 1 |  |  |  |  |  |
| LAMA/LABA | 0.58 (0.28-1.1) | 1.2 (0.85-1.77) | 1 |  |  |  |  |
| ICS/LABA | 0.61 (0.31-1.16) | 1.3 (0.93-1.76) | 1.08 (0.72-1.53) | 1 |  |  |  |
| LAMA | 0.62 (0.33-1.11) | 1.29 (0.86-2.03) | 1.06 (0.74-1.64) | 1 (0.67-1.54) | 1 |  |  |
| LABA | 0.85 (0.47-1.58) | 1.8 (1.18-2.91) | 1.49 (0.97-2.45) | 1.4 (0.95-2.09) | 1.38 (0.97-2.15) | 1 |  |
| ICS | 0.5 (0.18-1.38) | 1.05 (0.32-3.66) | 0.87 (0.26-3) | 0.81 (0.24-2.82) | 0.82 (0.25-2.69) | 0.58 (0.18-1.91) | 1 |
| Total exacerbation ≥2 or severe exacerbation ≥1 in the past year (3 studies, 3,438 patients) | | | | | | | |
| SUCRA | 0.176 | 0.849 | - | 0.734 | - | 0.240 | - |
| Rank | 4 | 1 | - | 2 | - | 3 | - |
| Comparison, median OR with 95% CrI |  |  |  |  |  |  |  |
| Placebo | 1 |  |  |  |  |  |  |
| ICS/LAMA/LABA | 0.05 (0.00-7.61) | 1 |  |  |  |  |  |
| LAMA/LABA | - | - | - |  |  |  |  |
| ICS/LABA | 0.08 (0.00-4.22) | 1.41 (0.09-24.85) | - | 1 |  |  |  |
| LAMA | - | - | - | - | - |  |  |
| LABA | 0.82 (0.05-14.46) | 15.05 (0.27-907.4) | - | 10.72 (0.7-165.9) | - | 1 |  |
| ICS | - | - | - | - | - | - | - |
| mMRC scale ≥2 or CAT score ≥10 (17 studies, 47,648 patients) | | | | | | | |
| SUCRA | 0.099 | 0.971 | 0.778 | 0.553 | 0.688 | 0.239 | 0.172 |
| Rank | 7 | 1 | 2 | 4 | 3 | 5 | 6 |
| Comparison, median OR with 95% CrI |  |  |  |  |  |  |  |
| Placebo | 1 |  |  |  |  |  |  |
| ICS/LAMA/LABA | 0.62 (0.48-0.79) | 1 |  |  |  |  |  |
| LAMA/LABA | 0.68 (0.54-0.87) | 1.11 (0.94-1.3) | 1 |  |  |  |  |
| ICS/LABA | 0.77 (0.66-0.92) | 1.24 (1.05-1.52) | 1.13 (0.96-1.34) | 1 |  |  |  |
| LAMA | 0.72 (0.55-0.95) | 1.16 (0.94-1.46) | 1.05 (0.82-1.34) | 0.93 (0.74-1.16) | 1 |  |  |
| LABA | 0.94 (0.8-1.12) | 1.52 (1.24-1.91) | 1.38 (1.11-1.7) | 1.22 (1.06-1.39) | 1.31 (1.03-1.68) | 1 |  |
| ICS | 0.97 (0.82-1.17) | 1.56 (1.25-2.02) | 1.41 (1.13-1.78) | 1.25 (1.07-1.48) | 1.35 (1.04-1.77) | 1.03 (0.88-1.22) | 1 |
| Study duration of ≥24 weeks (62 studies, 112,435 patients) | | | | | | | |
| SUCRA | 0.01 | 0.998 | 0.794 | 0.601 | 0.598 | 0.273 | 0.227 |
| Rank | 7 | 1 | 2 | 3 | 4 | 5 | 6 |
| Comparison, median OR with 95% CrI |  |  |  |  |  |  |  |
| Placebo | 1 |  |  |  |  |  |  |
| ICS/LAMA/LABA | 0.57 (0.47-0.68) | 1 |  |  |  |  |  |
| LAMA/LABA | 0.69 (0.59-0.79) | 1.2 (1.03-1.41) | 1 |  |  |  |  |
| ICS/LABA | 0.74 (0.64-0.85) | 1.29 (1.1-1.51) | 1.08 (0.94-1.23) | 1 |  |  |  |
| LAMA | 0.74 (0.67-0.82) | 1.29 (1.1-1.54) | 1.07 (0.96-1.22) | 1 (0.88-1.15) | 1 |  |  |
| LABA | 0.86 (0.77-0.96) | 1.5 (1.27-1.8) | 1.25 (1.1-1.44) | 1.17 (1.02-1.34) | 1.17 (1.04-1.3) | 1 |  |
| ICS | 0.88 (0.75-1.03) | 1.54 (1.25-1.89) | 1.28 (1.07-1.54) | 1.19 (1.01-1.4) | 1.19 (1.01-1.41) | 1.02 (0.87-1.2) | 1 |
| Study duration of ≥48 weeks (33 studies, 86,684 patients) | | | | | | | |
| SUCRA | 0.046 | 0.995 | 0.769 | 0.536 | 0.68 | 0.288 | 0.185 |
| Rank | 7 | 1 | 2 | 4 | 3 | 5 | 6 |
| Comparison, median OR with 95% CrI |  |  |  |  |  |  |  |
| Placebo | 1 |  |  |  |  |  |  |
| ICS/LAMA/LABA | 0.63 (0.53-0.75) | 1 |  |  |  |  |  |
| LAMA/LABA | 0.74 (0.64-0.85) | 1.17 (1.01-1.37) | 1 |  |  |  |  |
| ICS/LABA | 0.8 (0.7-0.93) | 1.27 (1.09-1.49) | 1.09 (0.95-1.24) | 1 |  |  |  |
| LAMA | 0.76 (0.68-0.85) | 1.2 (1.03-1.41) | 1.03 (0.92-1.15) | 0.95 (0.84-1.07) | 1 |  |  |
| LABA | 0.89 (0.79-1.01) | 1.42 (1.2-1.67) | 1.21 (1.07-1.36) | 1.11 (0.99-1.25) | 1.18 (1.06-1.3) | 1 |  |
| ICS | 0.94 (0.78-1.13) | 1.49 (1.19-1.87) | 1.27 (1.04-1.56) | 1.17 (0.98-1.41) | 1.24 (1.02-1.49) | 1.05 (0.87-1.26) | 1 |

CrI: credible interval, CAT: chronic obstructive pulmonary disease assessment test, FEV1: forced expiratory volume in 1 second, ICS: inhaled corticosteroid, LABA: long-acting beta-agonist, LAMA: long-acting muscarinic antagonist, mMRC: modified medical research council, OR: odds ratio, SUCRA: surface under the cumulative ranking curve

Median odds ratio and 95% credible interval were calculated as a row to column ratio.
