# Supplementary material for: Comparisons of exacerbations and mortality among regular inhaled therapies for patients with stable chronic obstructive pulmonary disease: Systematic review and Bayesian network meta-analysis
Source: PLoS Med. 2019 Nov 15;16(11):e1002958. doi: 10.1371/journal.pmed.1002958 (PMC6857849; doi:10.1371/journal.pmed.1002958)
Supplement: S8 Table — CrI, credible interval; FEV1, forced expiratory volume in 1 second; mMRC, modified medical research council. (DOCX) [file pmed.1002958.s012.docx]

**S8 Table. Network meta-regression analysis evaluating the relationship between the covariates and moderate to severe exacerbations**

|  | Regression coefficient (beta), median | 95% CrI | P(beta<0) |
| --- | --- | --- | --- |
| Post-bronchodilator FEV1% of predicted (%) | -0.001 | -0.04, 0.04 | 0.491 |
| Total exacerbation ≥1 in the past year (%) | -0.01 | -0.03, 0.02 | 0.727 |
| Total exacerbation ≥2 or severe exacerbation ≥1 in the past year (%) | -0.004 | -0.05, 0.06 | 0.551 |
| Serum eosinophil (%)^a^ | 0.26 | -3.70, 4.17 | 0.454 |
| mMRC scale | -0.27 | -3.46, 2.55 | 0.572 |
| Reversibility (%) | 0.02 | -0.05, 0.09 | 0.242 |

CrI: credible interval, FEV1: forced expiratory volume in 1 second, mMRC: modified medical research council
