# Supplementary material for: Comparisons of exacerbations and mortality among regular inhaled therapies for patients with stable chronic obstructive pulmonary disease: Systematic review and Bayesian network meta-analysis
Source: PLoS Med. 2019 Nov 15;16(11):e1002958. doi: 10.1371/journal.pmed.1002958 (PMC6857849; doi:10.1371/journal.pmed.1002958)
Supplement: S9 Table — Median OR and 95% CrI were calculated as a row to column ratio. CAT, chronic obstructive pulmonary disease assessment test; CrI, credible interval; FEV1, forced expiratory volume in 1 second; ICS, inhaled corticosteroid; LABA, long-acting beta-agonist; LAMA, long-acting muscarinic antagonist; mMRC, modified medical research council; OR, odds ratio; SUCRA, surface under the cumulative ranking curve. (DOCX) [file pmed.1002958.s013.docx]

**S9 Table. Sensitivity analyses of the drug classes to evaluate the effectiveness for reducing all-cause mortality**

|  | Placebo | ICS/LAMA/LABA | LAMA/LABA | ICS/LABA | LAMA | LABA | ICS |
| --- | --- | --- | --- | --- | --- | --- | --- |
| Post-bronchodilator FEV1 ≤60% (38 studies, 57,816 patients) | | | | | | | |
| SUCRA | 0.418 | 0.892 | 0.345 | 0.761 | 0.059 | 0.601 | 0.423 |
| Rank | 5 | 1 | 6 | 2 | 7 | 3 | 4 |
| Comparison, median OR with 95% CrI |  |  |  |  |  |  |  |
| Placebo | 1 |  |  |  |  |  |  |
| ICS/LAMA/LABA | 0.77 (0.53-1.12) | 1 |  |  |  |  |  |
| LAMA/LABA | 1.05 (0.74-1.47) | 1.37 (0.93-2) | 1 |  |  |  |  |
| ICS/LABA | 0.86 (0.69-1.12) | 1.13 (0.81-1.58) | 0.82 (0.59-1.16) | 1 |  |  |  |
| LAMA | 1.22 (0.95-1.56) | 1.58 (1.12-2.24) | 1.16 (0.89-1.52) | 1.41 (1.07-1.83) | 1 |  |  |
| LABA | 0.92 (0.73-1.2) | 1.21 (0.83-1.78) | 0.88 (0.62-1.28) | 1.07 (0.86-1.33) | 0.76 (0.58-1.02) | 1 |  |
| ICS | 1 (0.68-1.29) | 1.3 (0.8-1.95) | 0.95 (0.59-1.4) | 1.17 (0.77-1.49) | 0.82 (0.54-1.12) | 1.09 (0.73-1.38) | 1 |
| Post-bronchodilator FEV1 ≤50% (23 studies, 28,319 patients) | | | | | | | |
| SUCRA | 0.514 | 0.604 | 0.213 | 0.478 | 0.103 | 0.447 | 0.748 |
| Rank | 3 | 2 | 6 | 4 | 7 | 5 | 1 |
| Comparison, median OR with 95% CrI |  |  |  |  |  |  |  |
| Placebo | 1 |  |  |  |  |  |  |
| ICS/LAMA/LABA | 0.96 (0.44-2.13) | 1 |  |  |  |  |  |
| LAMA/LABA | 1.4 (0.56-3.61) | 1.46 (0.81-2.63) | 1 |  |  |  |  |
| ICS/LABA | 1.05 (0.54-2.13) | 1.1 (0.72-1.68) | 0.75 (0.39-1.47) | 1 |  |  |  |
| LAMA | 1.52 (0.71-3.42) | 1.59 (1.02-2.47) | 1.09 (0.62-1.94) | 1.45 (0.92-2.26) | 1 |  |  |
| LABA | 1.08 (0.56-2.11) | 1.13 (0.68-1.84) | 0.77 (0.39-1.54) | 1.02 (0.73-1.42) | 0.71 (0.43-1.14) | 1 |  |
| ICS | 0.74 (0.3-1.71) | 0.76 (0.31-1.79) | 0.52 (0.19-1.39) | 0.69 (0.31-1.47) | 0.48 (0.19-1.12) | 0.68 (0.3-1.43) | 1 |
| Total exacerbation ≥1 in the past year (36 studies, 68,824 patients) | | | | | | | |
| SUCRA | 0.457 | 0.836 | 0.333 | 0.524 | 0.248 | 0.336 | 0.766 |
| Rank | 4 | 1 | 6 | 3 | 7 | 5 | 2 |
| Comparison, median OR with 95% CrI |  |  |  |  |  |  |  |
| Placebo | 1 |  |  |  |  |  |  |
| ICS/LAMA/LABA | 0.82 (0.5-1.38) | 1 |  |  |  |  |  |
| LAMA/LABA | 1.06 (0.64-1.77) | 1.28 (0.98-1.68) | 1 |  |  |  |  |
| ICS/LABA | 0.97 (0.61-1.56) | 1.18 (0.93-1.52) | 0.92 (0.71-1.2) | 1 |  |  |  |
| LAMA | 1.1 (0.68-1.79) | 1.33 (1-1.79) | 1.04 (0.81-1.32) | 1.13 (0.85-1.49) | 1 |  |  |
| LABA | 1.05 (0.68-1.67) | 1.28 (0.93-1.75) | 1 (0.73-1.34) | 1.08 (0.85-1.36) | 0.96 (0.72-1.25) | 1 |  |
| ICS | 0.79 (0.4-1.49) | 0.95 (0.48-1.8) | 0.74 (0.37-1.4) | 0.8 (0.42-1.46) | 0.71 (0.36-1.34) | 0.75 (0.39-1.35) | 1 |
| Total exacerbation ≥2 or severe exacerbation ≥1 in the past year (4 studies, 4,250 patients) | | | | | | | |
| SUCRA | 0.302 | 0.702 | - | 0.532 | - | 0.367 | 0.596 |
| Rank | 5 | 1 | - | 3 | - | 4 | 2 |
| Comparison, median OR with 95% CrI |  |  |  |  |  |  |  |
| Placebo | 1 |  |  |  |  |  |  |
| ICS/LAMA/LABA | 0.44 (0.03-7.21) | 1 |  |  |  |  |  |
| LAMA/LABA | - | - | - |  |  |  |  |
| ICS/LABA | 0.70 (0.14-3.66) | 1.56 (0.17-14.92) | - | 1 |  |  |  |
| LAMA | - | - | - | - | - |  |  |
| LABA | 0.90 (0.24-3.52) | 2.04 (0.15-27.87) | - | 1.28 (0.35-4.75) | - | 1 |  |
| ICS | 0.61 (0.08-4.43) | 1.37 (0.07-27.73) | - | 0.88 (0.11-6.55) | - | 0.69 (0.10-4.71) | 1 |
| mMRC scale ≥2 or CAT score ≥10 (42 studies, 68,540 patients) | | | | | | | |
| SUCRA | 0.37 | 0.902 | 0.492 | 0.669 | 0.072 | 0.344 | 0.64 |
| Rank | 5 | 1 | 4 | 2 | 7 | 6 | 3 |
| Comparison, median OR with 95% CrI |  |  |  |  |  |  |  |
| Placebo | 1 |  |  |  |  |  |  |
| ICS/LAMA/LABA | 0.75 (0.49-1.13) | 1 |  |  |  |  |  |
| LAMA/LABA | 0.95 (0.63-1.38) | 1.26 (0.9-1.75) | 1 |  |  |  |  |
| ICS/LABA | 0.88 (0.67-1.19) | 1.17 (0.87-1.66) | 0.93 (0.71-1.28) | 1 |  |  |  |
| LAMA | 1.3 (0.83-2.02) | 1.72 (1.14-2.63) | 1.37 (0.88-2.14) | 1.47 (0.98-2.17) | 1 |  |  |
| LABA | 1.01 (0.77-1.46) | 1.34 (0.92-2.14) | 1.07 (0.75-1.66) | 1.14 (0.89-1.58) | 0.78 (0.51-1.27) | 1 |  |
| ICS | 0.89 (0.61-1.23) | 1.18 (0.75-1.84) | 0.94 (0.61-1.44) | 1.01 (0.69-1.37) | 0.69 (0.42-1.11) | 0.89 (0.58-1.18) | 1 |
| Study duration of ≥24 weeks (124 studies, 184,663 patients) | | | | | | | |
| SUCRA | 0.172 | 0.955 | 0.428 | 0.801 | 0.297 | 0.448 | 0.398 |
| Rank | 7 | 1 | 4 | 2 | 6 | 3 | 5 |
| Comparison, median OR with 95% CrI |  |  |  |  |  |  |  |
| Placebo | 1 |  |  |  |  |  |  |
| ICS/LAMA/LABA | 0.76 (0.61-0.96) | 1 |  |  |  |  |  |
| LAMA/LABA | 0.94 (0.79-1.14) | 1.24 (0.99-1.56) | 1 |  |  |  |  |
| ICS/LABA | 0.85 (0.75-0.98) | 1.12 (0.91-1.38) | 0.9 (0.75-1.09) | 1 |  |  |  |
| LAMA | 0.97 (0.87-1.11) | 1.28 (1.02-1.61) | 1.03 (0.87-1.22) | 1.14 (0.98-1.33) | 1 |  |  |
| LABA | 0.94 (0.83-1.07) | 1.24 (0.98-1.56) | 1 (0.82-1.21) | 1.1 (0.97-1.25) | 0.97 (0.84-1.12) | 1 |  |
| ICS | 0.95 (0.82-1.09) | 1.25 (0.97-1.59) | 1.01 (0.81-1.24) | 1.12 (0.95-1.29) | 0.98 (0.81-1.15) | 1.01 (0.86-1.17) | 1 |
| Study duration of ≥48 weeks (70 studies, 131,602 patients) | | | | | | | |
| SUCRA | 0.195 | 0.966 | 0.409 | 0.809 | 0.27 | 0.468 | 0.384 |
| Rank | 7 | 1 | 4 | 2 | 6 | 3 | 5 |
| Comparison, median OR with 95% CrI |  |  |  |  |  |  |  |
| Placebo | 1 |  |  |  |  |  |  |
| ICS/LAMA/LABA | 0.74 (0.58-0.94) | 1 |  |  |  |  |  |
| LAMA/LABA | 0.95 (0.78-1.17) | 1.29 (1.01-1.64) | 1 |  |  |  |  |
| ICS/LABA | 0.84 (0.74-0.97) | 1.14 (0.92-1.42) | 0.89 (0.72-1.08) | 1 |  |  |  |
| LAMA | 0.98 (0.87-1.14) | 1.34 (1.06-1.69) | 1.04 (0.87-1.25) | 1.17 (1-1.38) | 1 |  |  |
| LABA | 0.94 (0.83-1.07) | 1.27 (1-1.61) | 0.99 (0.8-1.21) | 1.11 (0.98-1.28) | 0.95 (0.81-1.11) | 1 |  |
| ICS | 0.96 (0.83-1.1) | 1.3 (1-1.67) | 1.01 (0.8-1.26) | 1.14 (0.97-1.32) | 0.97 (0.8-1.15) | 1.02 (0.87-1.18) | 1 |

CrI: credible interval, CAT: chronic obstructive pulmonary disease assessment test, FEV1: forced expiratory volume in 1 second, ICS: inhaled corticosteroid, LABA: long-acting beta-agonist, LAMA: long-acting muscarinic antagonist, mMRC: modified medical research council, OR: odds ratio, SUCRA: surface under the cumulative ranking curve

Median odds ratio and 95% credible interval were calculated as a row to column ratio.
