# Supplementary material for: Comparisons of exacerbations and mortality among regular inhaled therapies for patients with stable chronic obstructive pulmonary disease: Systematic review and Bayesian network meta-analysis
Source: PLoS Med. 2019 Nov 15;16(11):e1002958. doi: 10.1371/journal.pmed.1002958 (PMC6857849; doi:10.1371/journal.pmed.1002958)
Supplement: S10 Table — CrI, credible interval; FEV1, forced expiratory volume in 1 second; mMRC, modified medical research council. (DOCX) [file pmed.1002958.s014.docx]

**S10 Table. Network meta-regression analysis evaluating the relationship between the covariates and all-cause mortality**

|  | Regression coefficient (beta), median | 95% CrI | P(beta<0) |
| --- | --- | --- | --- |
| Post-bronchodilator FEV1% of predicted (%) | -0.12 | -0.16, -0.09 | 1.000 |
| Total exacerbation ≥1 in the past year (%) | -0.04 | -0.07, -0.01 | 0.999 |
| Total exacerbation ≥2 or severe exacerbation ≥1 in the past year (%) | -0.04 | -0.11, 0.03 | 0.851 |
| Serum eosinophil (%) | -1.16 | -4.80, 2.62 | 0.735 |
| mMRC scale | -2.57 | -4.62, -0.53 | 0.993 |
| Reversibility (%) | -0.16 | -0.26, -0.08 | 1.000 |

CrI: credible interval, FEV1: forced expiratory volume in 1 second, mMRC: modified medical research council
