# Supplementary material for: Comparisons of exacerbations and mortality among regular inhaled therapies for patients with stable chronic obstructive pulmonary disease: Systematic review and Bayesian network meta-analysis
Source: PLoS Med. 2019 Nov 15;16(11):e1002958. doi: 10.1371/journal.pmed.1002958 (PMC6857849; doi:10.1371/journal.pmed.1002958)
Supplement: S11 Table — Median OR and 95% CrI were calculated as a row to column ratio. CAT, chronic obstructive pulmonary disease assessment test; CrI, credible interval; FEV1, forced expiratory volume in 1 second; ICS, inhaled corticosteroid; LABA, long-acting beta-agonist; LAMA, long-acting muscarinic antagonist; mMRC, modified medical research council; NMA, network meta-analysis; OR, odds ratio; SUCRA, surface under the cumulative ranking curve. (DOCX) [file pmed.1002958.s015.docx]

**S11 Table. Network meta-analysis adjusted by covariates evaluating effectiveness in reducing all-cause mortality**

|  | Placebo | ICS/LAMA/LABA | LAMA/LABA | ICS/LABA | LAMA | LABA | ICS |
| --- | --- | --- | --- | --- | --- | --- | --- |
| Post- bronchodilator FEV1% of predicted (%) (149 studies, 188,721 patients) | | | | | | | |
| SUCRA | 0.244 | 0.958 | 0.407 | 0.777 | 0.235 | 0.539 | 0.34 |
| Rank | 6 | 1 | 4 | 2 | 7 | 3 | 5 |
| Comparison, median OR with 95% CrI |  |  |  |  |  |  |  |
| Placebo | 1 |  |  |  |  |  |  |
| ICS/LAMA/LABA | 0.76 (0.59-0.98) | 1 |  |  |  |  |  |
| LAMA/LABA | 0.96 (0.79-1.18) | 1.27 (1-1.62) | 1 |  |  |  |  |
| ICS/LABA | 0.87 (0.76-1.01) | 1.15 (0.91-1.45) | 0.9 (0.75-1.1) | 1 |  |  |  |
| LAMA | 1 (0.88-1.17) | 1.32 (1.04-1.69) | 1.04 (0.88-1.25) | 1.15 (0.98-1.37) | 1 |  |  |
| LABA | 0.93 (0.82-1.07) | 1.23 (0.96-1.58) | 0.97 (0.79-1.19) | 1.07 (0.93-1.23) | 0.93 (0.79-1.08) | 1 |  |
| ICS | 0.98 (0.84-1.14) | 1.29 (0.98-1.68) | 1.02 (0.81-1.27) | 1.12 (0.95-1.32) | 0.98 (0.8-1.17) | 1.05 (0.89-1.22) | 1 |
| Total exacerbation in the past year (%) (75 studies, 120,883 patients) | | | | | | | |
| SUCRA | 0.134 | 0.973 | 0.446 | 0.688 | 0.203 | 0.359 | 0.697 |
| Rank | 7 | 1 | 4 | 3 | 6 | 5 | 2 |
| Comparison, median OR with 95% CrI |  |  |  |  |  |  |  |
| Placebo | 1 |  |  |  |  |  |  |
| ICS/LAMA/LABA | 0.66 (0.49-0.87) | 1 |  |  |  |  |  |
| LAMA/LABA | 0.89 (0.69-1.15) | 1.36 (1.05-1.77) | 1 |  |  |  |  |
| ICS/LABA | 0.82 (0.68-0.99) | 1.25 (0.98-1.61) | 0.92 (0.74-1.15) | 1 |  |  |  |
| LAMA | 0.97 (0.8-1.19) | 1.48 (1.14-1.96) | 1.09 (0.89-1.34) | 1.19 (0.97-1.44) | 1 |  |  |
| LABA | 0.92 (0.77-1.1) | 1.4 (1.06-1.86) | 1.03 (0.81-1.31) | 1.12 (0.95-1.31) | 0.95 (0.77-1.15) | 1 |  |
| ICS | 0.81 (0.63-1.01) | 1.23 (0.89-1.7) | 0.91 (0.67-1.21) | 0.99 (0.78-1.23) | 0.83 (0.63-1.08) | 0.88 (0.69-1.09) | 1 |
| mMRC scale (17 studies, 18,500 patients) | | | | | | | |
| SUCRA | 0.43 | 0.668 | 0.525 | 0.419 | 0.775 | 0.233 | 0.451 |
| Rank | 5 | 2 | 3 | 6 | 1 | 7 | 4 |
| Comparison, median OR with 95% CrI |  |  |  |  |  |  |  |
| Placebo | 1 |  |  |  |  |  |  |
| ICS/LAMA/LABA | 0.7 (0.14-3.7) | 1 |  |  |  |  |  |
| LAMA/LABA | 0.88 (0.27-3.16) | 1.25 (0.23-7.44) | 1 |  |  |  |  |
| ICS/LABA | 1.01 (0.42-2.57) | 1.46 (0.35-5.76) | 1.16 (0.37-3.37) | 1 |  |  |  |
| LAMA | 0.55 (0.14-2.03) | 0.79 (0.11-5.2) | 0.62 (0.13-2.48) | 0.54 (0.12-2.15) | 1 |  |  |
| LABA | 1.29 (0.5-3.41) | 1.87 (0.36-9.03) | 1.48 (0.47-4.14) | 1.28 (0.57-2.75) | 2.38 (0.6-10.28) | 1 |  |
| ICS | 0.99 (0.21-4.15) | 1.42 (0.18-9.1) | 1.13 (0.18-5.5) | 0.98 (0.22-3.62) | 1.81 (0.26-11.77) | 0.77 (0.17-3) | 1 |
| Reversibility (%) (114 studies, 138,116 patients) | | | | | | | |
| SUCRA | 0.164 | 0.855 | 0.553 | 0.726 | 0.262 | 0.473 | 0.466 |
| Rank | 7 | 1 | 3 | 2 | 6 | 4 | 5 |
| Comparison, median OR with 95% CrI |  |  |  |  |  |  |  |
| Placebo | 1 |  |  |  |  |  |  |
| ICS/LAMA/LABA | 0.75 (0.52-1.09) | 1 |  |  |  |  |  |
| LAMA/LABA | 0.89 (0.69-1.16) | 1.19 (0.81-1.73) | 1 |  |  |  |  |
| ICS/LABA | 0.85 (0.72-1.02) | 1.14 (0.78-1.65) | 0.96 (0.73-1.25) | 1 |  |  |  |
| LAMA | 0.98 (0.83-1.21) | 1.31 (0.92-1.88) | 1.1 (0.86-1.42) | 1.15 (0.93-1.46) | 1 |  |  |
| LABA | 0.92 (0.77-1.07) | 1.23 (0.82-1.8) | 1.03 (0.77-1.35) | 1.08 (0.89-1.26) | 0.94 (0.72-1.16) | 1 |  |
| ICS | 0.92 (0.77-1.09) | 1.23 (0.82-1.82) | 1.03 (0.77-1.38) | 1.08 (0.89-1.29) | 0.94 (0.72-1.18) | 1 (0.84-1.22) | 1 |

CrI: credible interval, CAT: chronic obstructive pulmonary disease assessment test, FEV1: forced expiratory volume in 1 second, ICS: inhaled corticosteroid, LABA: long-acting beta-agonist, LAMA: long-acting muscarinic antagonist, mMRC: modified medical research council, OR: odds ratio, SUCRA: surface under the cumulative ranking curve

Median odds ratio and 95% credible interval were calculated as a row to column ratio.
